# Associations Between Breast Cancer Survivorship and Adverse Mental Health Outcomes: A Systematic Review

**DOI:** 10.1093/jnci/djy177

**Published:** 2018-11-07

**Authors:** Helena Carreira, Rachael Williams, Martin Müller, Rhea Harewood, Susannah Stanway, Krishnan Bhaskaran

**Affiliations:** 1 Department of Non-Communicable Disease Epidemiology, Faculty of Epidemiology and Population Health, London School of Hygiene & Tropical Medicine, London, UK; 2 Clinical Practice Research Datalink (CPRD), Medicines and Healthcare Products Regulatory Agency, London, UK; 3 Department of Emergency Medicine, Inselspital, Bern University Hospital, University of Bern, Bern, Switzerland; 4 Institute of Health Economics and Clinical Epidemiology, University Hospital of Cologne, Cologne, Germany; 5 Department of Medicine, The Royal Marsden NHS Foundation Trust, London and Surrey, UK

## Abstract

**Background:**

We aimed to systematically review the evidence on adverse mental health outcomes in breast cancer survivors (≥1 year) compared with women with no history of cancer.

**Methods:**

Studies were identified by searching MEDLINE, PsycINFO, the Cumulative Index to Nursing and Allied Health Literature, and the Social Sciences Citation Index, and through backward citation tracking. Two researchers selected the studies, extracted data, and assessed the risk of bias.

**Results:**

Sixty studies were included. Of 38 studies of depression, 33 observed more depression in breast cancer survivors; this was statistically significant in 19 studies overall, including six of seven where depression was ascertained clinically, three of four studies of antidepressants, and 13 of 31 that quantified depressive symptoms. Of 21 studies of anxiety, 17 observed more anxiety in breast cancer survivors, statistically significant in 11 studies overall, including two of four with clinical/prescription-based outcomes, and in eight of 17 of anxiety symptoms. Breast cancer survivors also had statistically significantly increased symptoms/frequency of neurocognitive dysfunction (18 of 24 studies), sexual dysfunctions (5 of 6 studies), sleep disturbance (5 of 5 studies), stress-related disorders/PTSD (2 of 3 studies), suicide (2 of 2 studies), somatisation (2 of 2 studies), and bipolar and obsessive-compulsive disorders (1 of 1 study each). Studies were heterogeneous in terms of participants’ characteristics, time since diagnosis, ascertainment of outcomes, and measures reported. Approximately one-half of the studies were at high risk of selection bias and confounding by socio-economic status.

**Conclusions:**

There is compelling evidence of an increased risk of anxiety, depression and suicide, and neurocognitive and sexual dysfunctions in breast cancer survivors compared with women with no prior cancer. This information can be used to support evidence-based prevention and management strategies. Further population-based and longitudinal research would help to better characterize these associations.

Women with a history of breast cancer are the largest group of cancer survivors in high-income countries (1). In the United States alone, more than 2.9 million women were estimated in 2012 to be living with a previous diagnosis of breast cancer (2). By 2022, this number is estimated to approach 4 million (2). Similarly, in the United Kingdom, the number of women living beyond breast cancer is expected to surpass 1.5 million during the next 20 years (3).

A diagnosis of breast cancer is often overwhelmingly distressing (4). Women frequently experience some combination of anger, anxiety, despair, helplessness, fear of death, and suicidal thoughts (5,6). Clinically relevant symptoms of anxiety and/or depression are common during the treatment period (7,8), when acute treatment side effects may restrict daily activities (9). High prevalence of depressive symptoms and anxiety have also been observed during survivorship (10,11), with one study finding depressive symptoms persisting for at least two years after diagnosis in one in five women (12). Other adverse mental health outcomes, such as sleep disturbance, have also been reported both during cancer treatment and afterwards (13). A substantial proportion of the breast cancer survivors experience long-term iatrogenic effects of treatment, including fatigue, persistent pain, lymphedema, vasomotor symptoms, and infertility, all of which may negatively affect quality of life and mental health (14). Other important psychological challenges in the long term can include difficulties in re-adapting to professional, social, and intimate relationships and coping with the uncertainty about the future (15).

To our knowledge, no systematic review to date has summarized the evidence from studies comparing breast cancer survivors with a noncancer control group for a broad spectrum of adverse mental health outcomes. Therefore, the aim of this study was to identify and summarize the studies that have quantitatively compared mental health outcomes in breast cancer survivors (≥1 year) vs women who did not have cancer; we also assessed the quality of the evidence on this topic by applying objective quality assessment criteria.

## Methods

This review was registered in the International Prospective Register of Systematic Reviews (PROSPERO 2017: CRD42017056946) and followed the a priori methods outlined in the protocol (published elsewhere [16]). Results were reported in accordance with the guidance of the Preferred Reporting Items for Systematic review and Meta-Analysis (17).

### Outcomes

The predefined outcomes of interest were anxiety disorders; bipolar and related disorders; disruptive, impulse control, and conduct disorders; feeding and eating disorders; mood disorders; neurocognitive disorders; neurotic disorders; personality disorders; schizophrenia spectrum and other psychotic disorders; sexual dysfunctions of psychological nature; sleep wake disorders; somatoform disorders; substance-related disorders (including alcoholism); and trauma- and stressor-related disorders. We also considered eligible the studies providing data on self-injurious behavior (including self-harm, suicide, and suicidal ideation). These categories were selected after systematically reviewing those listed in the Diagnostic and Statistical Manual of Mental Disorders, 5th edition (18) and in the ICD-10 Classification of Mental and Behavioural Disorder (19) to exclude conditions with usual onset during childhood or with strong genetic component (eg Huntington’s disease). The comprehensive list of outcomes was aimed at exploring what evidence was available on the topic without making strong assumptions as to whether the stress induced by the breast cancer diagnosis and treatment could trigger the condition. The outcomes of interest were disorders clinically diagnosed, but we also considered symptomatology evaluated with psychometric instruments.

### Data Sources and Identification

Potentially eligible studies were identified in four databases: MEDLINE, PsycINFO, the Cumulative Index to Nursing and Allied Health Literature, and the Social Sciences Citation Index. A search expression tailored for each database was created including terms for the exposure (breast cancer), outcomes (the predefined mental disorders), and comparators (eg, risk) (full MEDLINE search string provided in the Appendix [Supplementary Table 1, available online]). Results retrieved from the inception of the databases up to November 1, 2017 were considered for this study. Two authors screened the list of references by applying the same inclusion and exclusion criteria to determine each study’s eligibility. The bibliographic references of eligible studies were manually screened to detect additional studies.

### Study Eligibility

We considered as eligible observational studies that provided original data comparing the prevalence, incidence, or odds/hazard of at least one of the predefined lists of mental health outcomes (see above), clinically diagnosed or their symptomatology assessed through validated instruments, between adult female breast cancer survivors and a comparison group of women with no prior cancer. Female breast cancer survivors were defined as women with a history of breast cancer or in situ tumor for one year or longer. Studies with patients diagnosed with breast carcinomas in situ were included because despite of their excellent prognosis (20), they receive similar treatment to invasive breast cancers (21), and patients often experience substantial psychological distress both during and after the treatment period (22,23). Studies with no control group but reporting standardized incidence ratios were also eligible if the standardization was against a general female population. Studies that used psychometric instruments that had been altered from the standard/validated version were excluded, except where the alteration was limited to omission of questions that would not apply to the population under study. Studies including women who were institutionalized, under active treatment for breast cancer (excluding endocrine therapy), or who were specifically selected based on distressing psychological and/or physical symptoms were excluded. Studies evaluating the effect of further screening or diagnostic tests for cancer on the mental health of breast cancer survivors were excluded. There was no restriction in the language of study publication.

The eligibility of individual studies was assessed by two reviewers (HC and MM, or HC and RH) who independently applied the predefined inclusion/exclusion criteria. Initial agreement between reviewers in the assessment of abstracts was 92.5% for HC/MM and 81.3% for HC/RH (Cohen’s kappa [κ] = 0.51 and 0.32, respectively), and initial agreement in the full-text assessment was 95.9% (κ = 0.69) and 90.6% (κ = 0.54), respectively. All discordant assessments were discussed and successfully resolved.

### Data Extraction

We systematically abstracted data on the characteristics of the study and study samples. We extracted quantitative data on the frequency (incidence or prevalence) or severity (mean scores) of adverse mental health outcomes for each participant’s group or for the comparison between groups (eg, relative risk, hazard ratio, odds ratio), as available, and the results of any hypothesis testing reported in the original studies. Prevalences from studies involving psychometric instruments were based on the cut-offs defined by the authors of the original studies. When two or more studies reported data on the same study population, we extracted data from the study with largest sample size, or if equal, the one providing more detailed outcome information. Data were extracted independently by two investigators (HC and MM, or HC and RH) and discrepancies were resolved.

### Risk of Bias in Individual Studies

The risk of bias in the included studies was assessed by two reviewers who independently evaluated domains previously identified as important in observational studies (24). The domains were: participants’ selection, outcome assessment, temporality (breast cancer diagnosed prior to the onset of the mental health outcome), control for confounding by age and socio-economic status, statistical methods, handling of missing data, and disclosure of conflicts of interest. Within each domain, the studies were rated as having a high, low, or unclear risk of bias; some criteria were not applicable to all studies. Supplementary Table 2 (available online) provides the criteria used for each category and domain.

### Statistical Methods

Tables, graphs, and descriptive text were used to summarize study characteristics and results stratified by mental health outcome and method used to define outcomes (ie, clinical diagnosis, drug prescription, or symptoms). When sufficient information was provided in the original studies, we calculated the prevalence ratio for each outcome (25) if this was not directly reported in the paper. If prevalence data were provided by severity categories, we computed prevalence ratios for the comparison of mild to severe symptoms of the outcome between the two groups; this was the most common dichotomization in the studies that did not provide results by severity. The 95% confidence intervals (CIs) for derived prevalence ratios were estimated using the delta method (25). *P* values for the comparison of mean scores from psychometric instruments between breast cancer survivors and women who did not have cancer were estimated with the independent samples *t* test; all tests were two-sided. To ensure comparability of the results across studies, we applied a type-1 error rate (α) of .05 when summarizing statistical significance even if studies themselves had provided results using a different statistical significance level. A quantitative synthesis of the results (ie, meta-analysis), as planned in the study protocol (16), was not possible due to the heterogeneity of the eligible studies in the clinical characteristics of the cancer survivors, time elapsed since breast cancer diagnosis, and instruments used to evaluate symptoms of mental health disorders.

## Results

### Characteristics of Included Studies

**Figure 1.**
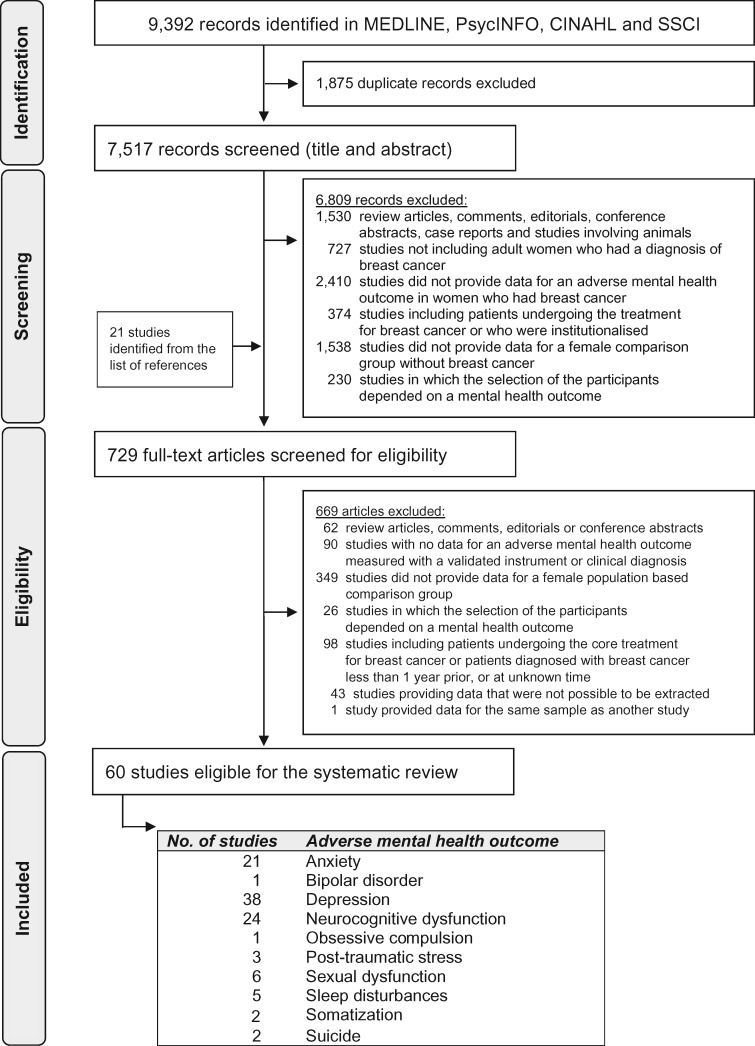
Systematic review flowchart. CINAHL = Cumulative Index to Nursing and Allied Health Literature; SSCI = Social Sciences Citation Index.

Of the 7517 individual publications identified, 729 studies were eligible for full-text evaluation, and 60 (26–85) were ultimately included (Figure 1). The most commonly evaluated outcomes were anxiety (n = 21 studies), depression (n = 38), neurocognitive dysfunction (n = 24), and sexual dysfunction (n = 6) (Table 1). Schairer et al. (41) estimated the risk of suicide in more than 720 000 women diagnosed with breast cancer in 1953 to 2001, using data from 16 population-based cancer registries in Scandinavia and the United States; thus, only two studies were eligible for suicide, because smaller studies with overlapping data were excluded. The studies were heterogeneous in study design, participants’ characteristics, and methods involved to assess outcomes. A total 38 of 60 studies (63.3%) included small, nonprobabilistic samples of breast cancer survivors. Mental health outcomes were most commonly evaluated with psychometric instruments (50/60 studies = 83.3%), followed by clinical diagnoses registered in electronic healthcare databases (10/60 = 16.7%).

**Table 1.** Summary of the main characteristics of the eligible studies (N = 60)

Study characteristic	Studies, n (%)
Type of study	
Cohort	22 (36.7)
Cross-sectional	38 (63.3)
Type of population	
Population-based	10 (16.7)
Convenience samples recruited at health institutions	43 (71.7)
Randomly selected	3 (5.0)
Convenience samples recruited from the community	7 (11.7)
Randomly selected	0 (0.0)
Characteristics of the women with history of breast cancer	
Mean/median age	
≤49 y	16 (26.7)
50–69 y	41 (68.3)
≥70 y	3 (5.0)
Mean/median time since diagnosis*	
∼1 y	12 (20.0)
>1 and ≤5 y	26 (43.3)
>5 and ≤10 y	17 (28.3)
>10 y	5 (8.3)
Sample size†	
<50	18 (30.0)
50–100	20 (33.3)
101–1000	14 (23.3)
>1000	8 (13.3)
Stage at diagnosis inclusion criteria	
In situ only	1 (1.7)
In situ and nonmetastatic invasive	6 (10.0)
In situ and invasive all stages	3 (5.0)
Invasive, nonmetastatic	30 (50.0)
Invasive, all stages	20 (33.3)
Treatment-related inclusion criteria	
Breast-conserving surgery	1 (1.7)
Mastectomy	5 (8.3)
Breast reconstruction	2 (3.3)
Chemotherapy	13 (21.7)
No chemotherapy	1 (1.7)
Hormone therapy	3 (5.0)
Radiotherapy	2 (3.3)
Immunotherapy	0 (0.0)
All treatments	33 (55.0)
Disease progression related inclusion criteria	
Only patients who did not have recurrence or relapse	15 (25.0)
Only patients who were tumor free at recruitment	12 (20.0)
Patients with disease recurrence included‡	19 (31.7)
Unclear	14 (23.3)
Adverse mental health outcome§	
Anxiety	21 (35.0)
Bipolar disorder	1 (1.7)
Depression	38 (63.3)
Neurocognitive dysfunction	24 (40.0)
Obsessive compulsion	1 (1.7)
Sexual dysfunction	6 (10.0)
Sleep disturbances	5 (8.3)
Stress-related / posttraumatic stress	3 (5.0)
Somatization	2 (3.3)
Suicide	2 (3.3)
Adverse mental health outcome assessment§	
Clinical diagnosis	10 (16.7)
Pharmacological treatment‖	5 (8.3)
Psychometric instruments	50 (83.3)

* Or mean/median time since treatment completion, as reported in the original studies.

† Refers to patients included in analysis.

‡ Includes studies that explicitly stated the inclusion of patients with recurrence, and longitudinal studies including newly diagnosed patients and that did not report exclusions related to recurrence/relapse during follow-up.

§ Studies may have provided data for more than one outcome and may have assessed one outcome by more than one method.

‖ Includes self-reported medication intake.

### Findings for Specific Mental Health Outcomes

**Figure 2.**
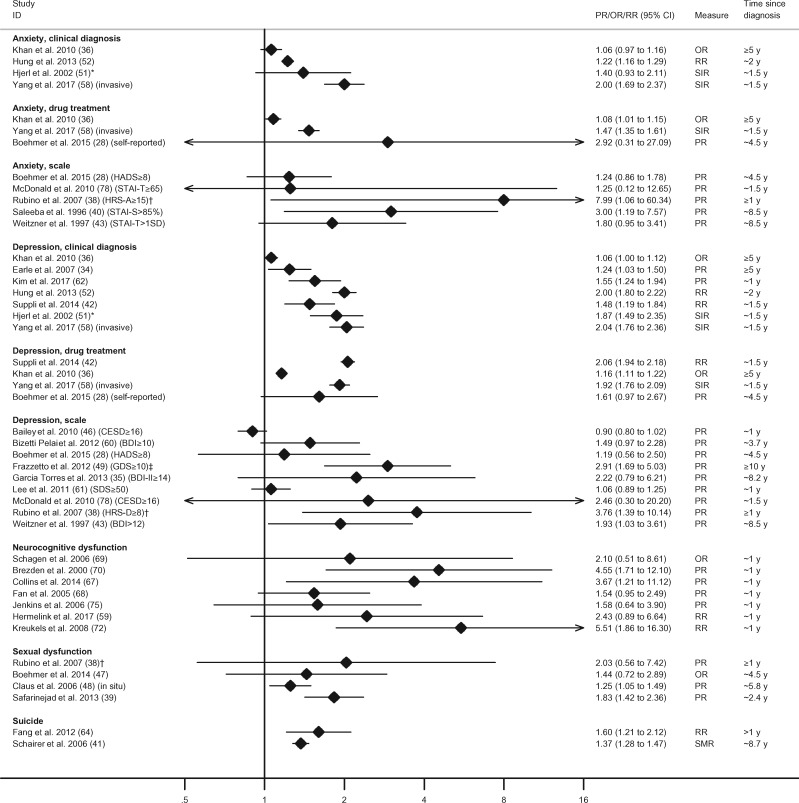
Associations between breast cancer history and anxiety, depression, neurocognitive and sexual dysfunctions, and suicide. We considered that anxiolytics were being taken to treat anxiety and antidepressants to treat depression. Time since diagnosis refers to the mean/median time elapsed since the breast cancer diagnosis or completion of initial course of treatment, as reported in the original studies, for the sample of cancer survivors. When this information was not reported in the original studies, we presented the lower limit of survivorship time reported in the inclusion criteria of the study. The minimum, mean/median, and maximum follow-up of longitudinal studies are reported in the Supplementary Appendix (available online). *The original study provided relative risk estimates stratified by area of residence (urban/rural). The combined estimate presented in the forest plot was computed with inverse-variance-weighted meta-analysis methods using the command “metan” in Stata v14. BDI(-II) = Beck Depression Inventory(-II); CESD = The Center for Epidemiologic Studies, Depression Scale; GDS = Geriatric Depression Scale; HADS = Hospital Anxiety and Depression Scale; HRS-A = Hamilton Rating Scale for Anxiety; HRS-D = Hamilton Rating Scale for Depression; OR = odds ratio; PR = prevalence ratio; RR = relative risk; SD = standard deviation; SDS = Self-rating Depression Scale; SIR = standardized incidence ratio; SMR = standardized mortality ratio; STAI-S = State-Trait Anxiety Inventory (state anxiety subscale); STAI-T = State-Trait Anxiety Inventory (trait anxiety subscale). †Women who have had breast reconstruction after mastectomy. ‡Refers to a group of women who had breast cancer recurrence 10 years after the first diagnosis.

**Figure 3.**
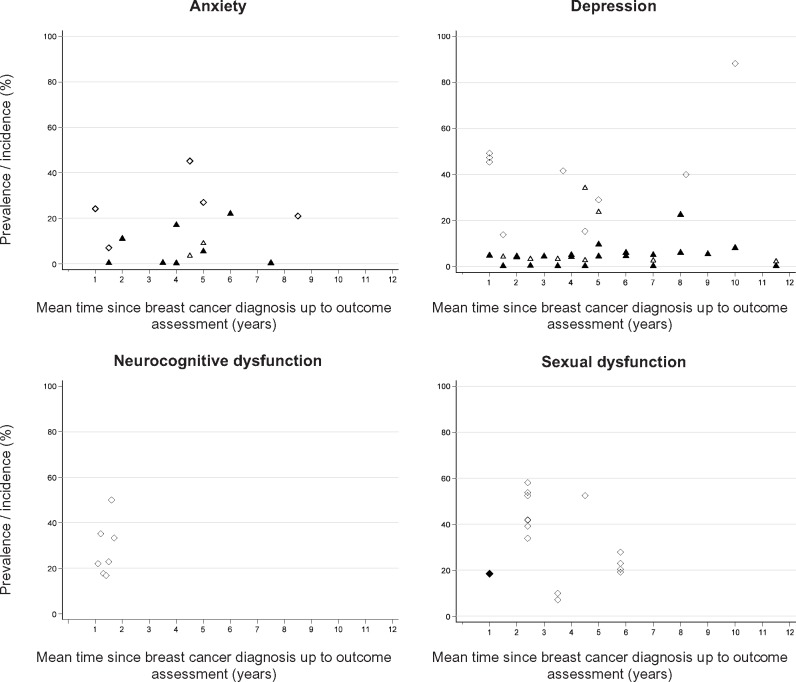
Absolute frequency of anxiety, depression, and neurocognitive and sexual dysfunctions reported in the original studies for breast cancer survivors. Estimates for cognitive and sexual dysfunctions refer to the prevalence of women impaired for the condition or specific domains, as reported in the original studies. EHR = electronic health records. **Black triangle** = cumulative incidence, diagnoses in EHR; **white triangle** = cumulative incidence, drug treatment; **white diamond** = prevalence, psychometric instruments; **black diamond** = prevalence, psychiatric interview.

Table 2 provides an overview of the directions of association reported for all studies/outcomes and statistical significance of the between-group comparisons. Figure 2 summarizes the relative measures of effect for the most commonly studied outcomes in the studies where these were available. Figure 3 shows the prevalence (for cross-sectional analyses) or cumulative incidence (for follow-up analyses) of outcomes in the samples of breast cancer survivors included in the original studies.

**Table 2.** Summary of the results of the studies included in the systematic review and studies’ quality assessment*

Study	Sample size (BCS/controls)	Anxiety			Bipolar	Depression			Neuro-cognitive dysfunction†	Obsessive compulsion	Sexual dysfunction†	Sleep disturbance†	Somatization	Stress-related disorders /PTSD	Suicide	Studies risk of bias assessment‡						
		Diag	Drug	Symp		Diag	Drug	Symp								Sel	Inf	Temp	Conf	Stat	Miss	COI
Aerts, 2014 (45)	114/149	—	—	—	—	—	—	H§	—	—	—	—	—	—	—	Y	?	Y	Y	N	?	N
Ahles, 2010 (77)	110/39	—	—	—	—	—	—	—	H§	—	—	—	—	—	—	Y	N	N	N	N	?	N
Amir, 2002 (26)	39/39	—	—	H§	—	—	—	H§	—	H§	—	—	H§	—	—	?	?	Y	N	N	?	Y
Ancoli-Israel, 2014 (27)	44/35	—	—	—	—	—	—	H§	—	—	—	H§	—	—	—	?	?	N	N	N	?	N
Bailey, 2010 (46)	515/496	—	—	—	—	—	—	L	—	—	—	—	—	—	—	?	?	Y	Y	N	?	N
Bizetti Pelai, 2012 (60)	89/43	—	—	—	—	—	—	H	—	—	—	—	—	—	—	?	?	Y	Y	—	?	Y
Boehmer, 2015 (28)	85/85	—	H	H	—	—	H	H	—	—	—	—	—	—	—	Y	?‖/Y¶	Y	Y	N	?	N
Boehmer, 2014 (47)	85/85	—	—	—	—	—	—	—	—	—	H§	—	—	—	—	Y	?	Y	Y	N	?	N
Boele, 2015 (29)	63/44	—	—	H	—	—	—	H	H§	—	—	—	—	—	—	Y	?	N	N	N	?	N
Brezden, 2000 (70)	40/36	—	—	—	—	—	—	—	H§	—	—	—	—	—	—	Y	?	Y	N	N	?	Y
Broeckel, 2002 (30)	58/61	—	—	—	—	—	—	H§	—	—	H§	—	—	—	—	Y	?	Y	Y	N	?	Y
Calvio, 2010 (31)	122/113	—	—	H§	—	—	—	H§	H§	—	—	—	—	—	—	Y	?	Y	N	N	?	N
Castellon, 2004 (74)	53/19	—	—	L§, #	—	—	—	L	H§	—	—	—	—	—	—	Y	?	Y	?	N	?	N
Claus, 2006 (48) (in situ)	795/702	—	—	—	—	—	—	H§	—	—	H§	—	—	—	—	Y	?	Y	N	N	?	N
Cohen, 2011 (32)	56/66	—	—	H§	—	—	—	H	—	—	—	—	H§	—	—	Y	?	Y	N	N	?	Y
Collins, 2014 (67)	54/54	—	—	—	—	—	—	—	H§	—	—	—	—	—	—	Y	N	N	N	N	?	N
Conroy, 2013 (83)	24/23	—	—	L	—	—	—	L	H§	—	—	—	—	—	—	?	?	Y	N	N	?	N
Dahl, 2011 (33)	337/1685	—	—	H§	—	—	—	L§	—	—	—	H§	—	—	—	?	?	Y	N	N	?	N
Earle, 2007 (34)	463/3108	—	—	—	—	H§	—	—	—	—	—	—	—	—	—	N	N	Y	N	—	—	Y
El Rafihi-Ferreira, 2011 (63)	50/50	—	—	—	—	—	—	—	—	—	—	H§	—	—	—	?	?	Y	Y	—	?	Y
Ernst, 2002 (85)	16/33	—	—	—	—	—	—	—	H	—	—	—	—	—	—	Y	?	Y	Y	N	?	N
Fan, 2005 (68)	91/83	—	—	—	—	—	—	—	H§	—	—	—	—	—	—	?	N	N	N	N	?	N
Fang, 2012 (64)	74 977/ND	—	—	—	—	—	—	—	—	—	—	—	—	—	H§	N	N	N	N	N	—	N
Frazzetto, 2012 (49) (R)	32/35	—	—	—	—	—	—	H§,**	—	—	—	—	—	—	—	?	?	Y	Y	—	?	N
Garcia-Torres, 2013 (35)	22/22	—	—	L	—	—	—	H	—	—	—	—	—	—	—	Y	?	Y	Y	N	?	Y
Gurevich, 2004 (50)	66/69	—	—	—	—	—	—	—	—	—	—	—	—	H§	—	Y	?	Y	Y	N	?	Y
Hermelink, 2017 (59)	56/150	—	—	—	—	—	—	H§	H††	—	—	—	—	—	—	?	?‖**/**N	Y	?	N	N	Y
Hjerl, 2002 (51)	60 431‡‡/ND	H	—	—	—	H§	—	—	—	—	—	—	—	—	—	N	N	N	Y	N	—	Y
Hung, 2013 (52)	26 629‡‡/26 629‡‡	H§	—	—	H§	H§	—	—	—	—	—	—	—	—	—	N	N	N	Y	N	—	N
Inagaki, 2007 (84)	105/55	—	—	—	—	—	—	—	H	—	—	—	—	—	—	Y	N	Y	Y	N	?	N
Jenkins, 2006 (75)	128/49	—	—	—	—	—	—	—	H	—	—	—	—	—	—	Y	N	N	N	N	N	N
Khan, 2010 (36)	16 938/67 649	H	H§	—	—	H	H§	—	—	—	—	—	—	—	—	N	N	N	N	N	—	N
Kim, 2017 (62)	2130/8520	—	—	—	—	H§	—	—	—	—	—	—	—	—	—	N	N	N	N	—	—	N
Klein, 2011 (53)	652/1188	—	—	H§	—	—	—	—	—	—	—	—	—	—	—	Y	?	Y	N	N	?	N
Koppelmans, 2012 (71)	196/1509	—	—	—	—	—	—	L§	H§	—	—	—	—	—	—	N	?	Y	N	N	?	N
Kreukels, 2008 (72)	63/60	—	—	H§	—	—	—	H§	H§	—	—	—	—	—	—	?	?‖**/**N	Y	Y	N	?	N
Kesler, 2013 (80)	42/35	—	—	—	—	—	—	H	H§	—	—	—	—	—	—	Y	?	Y	?	N	?	N
Lejbak, 2010 (82)	28/37	—	—	—	—	—	—	—	H§	—	—	—	—	—	—	Y	?	Y	?	N	N	Y
Lee, 2011 (61)	206/496	—	—	—	—	—	—	H	—	—	—	—	—	—	—	N	?	Y	N	N	?	N
McDonald, 2010 (78)	29/18	—	—	H	—	—	—	H	—	—	—	—	—	—	—	?	?	N	Y	N	?	N
Miao, 2016 (79)	23/26	—	—	H	—	—	—	H	H§	—	—	—	—	—	—	?	?	Y	Y	N	?	N
Min, 2010 (54)	52/104	—	—	—	—	—	—	H§	—	—	—	—	—	—	—	Y	?	Y	Y	—	?	Y
Myers, 2015 (66)	156/46	—	—	—	—	—	—	—	H§	—	—	—	—	—	—	Y	?	Y	?	N	N	N
Nguyen, 2013 (86)	57/30	—	—	—	—	—	—	H	H§	—	—	—	—	—	—	Y	?	Y	N	N	?	N
Otte, 2010 (37)	246/246	—	—	—	—	—	—	H§	—	—	—	H§	—	—	—	Y	?	Y	?	N	?	N
Phillips, 2012 (81)	129/184	—	—	—	—	—	—	—	H§	—	—	—	—		—	?	N	Y	N	N	?	N
Root, 2015 (65)	113/37	—	—	L	—	—	—	H	H§	—	—	—	—	—	—	?	?	Y	N	N	?	N
Rubino, 2007 (38)	33/33	—	—	H§	—	—	—	H§	—	—	H	—	—	—	—	?	N	Y	Y	N	?	Y
Safarinejad, 2013 (39)	186/204	—	—	—	—	—	—	—	—	—	H§	—	—	—	—	?	?	Y	N	N	?	N
Saleeba, 1996 (40)	52/88	—	—	H§	—	—	—	—	—	—	—	—	—	—	—	Y	?	Y	N	N	?	Y
Schairer, 2006 (41)	723 810/ND	—	—	—	—	—	—	—	—	—	—	—	—	—	H§	N	N	N	Y	N	—	N
Schagen, 2006 (69)	57/60	—	—	—	—	—	—	—	H	—	—	—	—	—	—	Y	N	N	Y	N	?	N
Silverman, 2007 (73)	24/10	—	—	—	—	—	—	—	H	—	—	—	—	—	—	?	?	Y	Y	N	?	N
Suppli, 2014 (42)	44 494‡‡/2M‡‡	—	—	—	—	H§	H§	—	—	—	—	—	—	—	—	N	N	N	Y	N	—	N
Vazquez-Ortiz, 2010 (55)	30/30	—	—	—	—	—	—	—	—	—	H§	—	—	—	—	?	?	Y	Y	N	?	Y
Voigt, 2016 (76)	150/56	—	—	—	—	—	—	—	—	—	—	—	—	H§§	—	?	?	N	N	N	N	N
Von Ah, 2012 (57)	62/78	—	—	—	—	—	—	H	—	—	—	H§	—	—	—	Y	?	Y	N	N	?	N
Von Ah, 2009 (56)	52/52	—	—	—	—	—	—	H	H§	—	—	—	—	—	—	Y	?	Y	N	N	?	N
Weitzner, 1997 (43)	60/93	—	—	H	—	—	—	H§	—	—	—	—	—	—	—	Y	?	Y	N	N	?	Y
Yang, 2017 (58) (Inv)	40 849/452 507	H§	H§	—	—	H§	H§	—	—	—	—	—	—	H§	—	N	N	N	Y	N	—	Y

* When a cohort study provided estimates for more than one point in time, we presented all data in the supplementary tables but described the estimate for only the first time point after the first anniversary of cancer diagnosis. When a study provided data for invasive and in situ tumors, we presented the results for the invasive tumors only. When a study provided data on the prevalence of subjects considered impaired as well as the mean scores of the psychometric instrument, we showed the result of the hypothesis test for the comparison of the prevalences. BCS = breast cancer survivors; COI = conflict of interest; Conf = confounding (by age and SES); Diag = clinical diagnoses; Drug = based on relevant drug prescriptions; H = study observed higher prevalence, risk, or severity of outcomes in breast cancer survivors compared with women who did not have cancer; Inf = information bias; Inv = invasive tumors; L = study observed lower prevalence, risk, or severity of outcomes in breast cancer survivors compared with women who did not have cancer; M = million; Miss = missing data; N = low risk of bias; R = recurrence; Sel = selection bias; Stat = statistical methods; Symp = based on symptoms; Temp = temporality of events; Y = high risk of bias; ? = unclear risk of bias.

† The risk of neurocognitive impairment, sexual dysfunction, and sleep disturbance was considered increased when the study reported statistically significant impairments in one or more domains of neurocognitive or sexual function, or sleep, respectively. When subjective and objective measures of neurocognitive function were provided, we considered the results of the objective measures.

‡ Risk of bias assessment was not applicable for the domain of statistical methods for studies where no results for formal statistical comparisons between the two groups were provided; risk of bias assessment was also not applicable for the missing data domain if the study was based on electronic health records.

§ Statistically significant (*P* < .05).

‖ For anxiety or depression outcomes.

¶ Self-reported anxiolytics and antidepressants intake.

# Breast cancer survivors reported lower symptoms for state anxiety compared with controls (*P* = .01). No statistically significant between-group differences were observed for trait anxiety (*P* = .08).

** Severe depression and mild to severe depression were increased in breast cancer survivors (*P* < .05). Mild depression did not differ between groups (*P* ≥ .05).

†† There was no strong statistical evidence of increased risk of cognitive dysfunction in women who had breast cancer compared with the healthy control group. However, strong statistical evidence for an increased frequency of cognitive dysfunction among breast cancer patients was found when considering the mean composite scores at one year (*P* < .05); the comparison remained statistically significant after correcting for multiple testing.

‡‡ Number of women at baseline; number of women at 1 year after diagnosis not reported. Suppli et al: 44 494 breast cancer survivors and 1 997 669 for depression analysis; the corresponding figures for the antidepressant analysis were 35 286 (exposed to breast cancer) and 1 860 552 (background population).

§§ There was no strong statistical evidence of an increased risk of PTSD in breast cancer survivors compared with women without cancer. However, the mean number of PTSD symptoms in breast cancer survivors was statistically significantly higher from the mean number of symptoms in the control group (*P* < .001).

### Anxiety

Twenty-one eligible studies reported data for anxiety (Table 1; Supplementary Table 3, available online). Of 21 studies, 17 (81.0%) observed increased anxiety in the breast cancer survivor group compared with the noncancer group; the difference was statistically significant in 11 of 21 (52.4%) studies (Table 2).

Four longitudinal, population-based studies evaluated anxiety with clinical diagnoses (n = 2) or clinical diagnoses and anxiolytics prescription (n = 2); all used electronic health records data and pointed towards an increased risk in breast cancer survivors, but this was supported by strong statistical evidence in two studies only (Figure 2). The relative risk estimates in the four studies of clinically assessed anxiety varied between 1.06 (95% CI = 0.97 to 1.16) and 2.00 (95% CI = 1.69 to 2.37). The two studies that reported on anxiolytics prescription reported an 8% (95% CI = 1% to 15%) and 47% (95% CI = 35% to 61%) increase in breast cancer survivors compared with women who did not have cancer (Figure 2).

Seventeen studies investigated symptoms of anxiety using scales (Table 2). There was strong statistical evidence of increased symptoms of anxiety in eight of 17 studies, including in the six of 12 studies that focused on comparing mean scores between groups, and in two of five studies that reported prevalence of scoring above a clinically relevant threshold. For all of the latter, observed prevalence was higher in cancer survivors but confidence intervals were generally wide (Figure 2).

Prevalences of anxiety were generally less than 20% when electronic health records or anxiolytics were studied and in the range of 20% to 50% when scales were used (Figure 3). Determinants of clinically assessed anxiety were provided in one study. Clinically diagnosed anxiety in breast cancer survivors tended to decrease over time since diagnosis (58) and was independently associated with younger age and presence of comorbidities at diagnosis, having less favorable tumor characteristics, and receiving chemotherapy (58).

### Depression and Suicide

Thirty-eight studies provided data on depression (Table 1; Supplementary Table 4, available online), and 33 of 38 (86.8%) described more depression in the breast cancer survivor group compared with women who did not have cancer, with 19 fo 38 (50.0%) reporting statistical evidence of increased depression (Table 2).

Of seven studies that analyzed depression based on clinical diagnoses, six found strong evidence of an elevated risk among breast cancer survivors, with relative risk estimates ranging from 1.06 (95% CI = 1.00 to 1.12) to 2.04 (95% CI = 1.76 to 2.36) (Figure 2). All four studies defining depression by antidepressant use found higher use in breast cancer survivors, though for one smaller study the confidence interval was wide and overlapped the null; relative risk estimates ranged between 1.16 (95% CI = 1.11 to 1.22) and 2.06 (95% CI = 1.94 to 2.18).

Of 31 studies that evaluated depressive symptoms with scales, 13 reported strong statistical evidence of higher severity of depressive symptoms among women who had breast cancer (Table 2); among these, eight of nine studies that focused on the prevalence of scoring above a clinically relevant threshold found higher prevalence in breast cancer survivors, but this was statistically significant in only three studies and most estimates again had wide confidence intervals (Figure 2).

The prevalence of depression in breast cancer survivors was highest when evaluated with self-reported instruments (with most estimates >30%) and lower for clinically diagnosed depression (most estimates <10%; Figure 3). Determinants of depression clinically assessed in breast cancer survivors were seldom reported. Independent predictors of clinically diagnosed depression included younger age, having comorbidities at diagnosis and less favorable tumor characteristics (42,58), living alone, and having lower levels of education (42).

Two studies of suicide found breast cancer survivors to have 37% (95% CI = 28% to 47%) to 60% (95% CI = 21% and 112%) higher risk than women in the comparison group (Figure 2).

### Neurocognitive Dysfunction

Twenty-four studies evaluated domains of neurocognitive function (Table 1; Supplementary Table 5, available online). All studies described that breast cancer survivors performed worse than noncancer controls for one or more domains of neurocognitive function (Table 2); this was supported by strong statistical evidence in 18 of 24 (75.0%) studies. When prevalence estimates were provided, all seven studies showed point estimates tending towards an increased neurocognitive dysfunction in breast cancer survivors compared with control subjects, even though this was supported by strong statistical evidence in only three instances; prevalence ratio estimates varied between 1.54 (95% CI = 0.95 to 2.49) and 5.51 (95% CI = 1.86 to 16.30) (Figure 2).

Of the 24 studies of neurocognitive dysfunction, 21 investigated the effect of being exposed to chemotherapy vs no chemotherapy; these studies consistently showed increased risk of neurocognitive impairments in breast cancer survivors exposed to chemotherapy. Three studies evaluated the effect of being exposed to hormone therapy in chemotherapy-naïve patients (29,82,85); two found strong evidence of increased neurocognitive dysfunction among breast cancer survivors exposed to hormone therapy. In most studies, neurocognitive impairments were described to affect 20% to 40% of women one year postdiagnosis (Figure 3).

### Sexual Dysfunction

Six studies, all involving convenience samples, reported data for sexual dysfunction (Table 1). Five of these reported impairments in one or more domains of sexual function (Table 2). All studies for which prevalence ratios were available showed increased dysfunction in breast cancer survivors, with relative risk estimates between 1.25 (95% CI = 1.05 to 1.49) and 2.03 (95% CI = 0.56 to 7.42) (Figure 2), but the width of the confidence intervals did not exclude the probability of this being due to chance in two studies. The prevalence of reported impaired sexual function overall or for specific domains was generally in the range of 20% to 60% (Figure 3). Safarinejad et al. (39) reported that women who had radiotherapy, chemotherapy, and hormone therapy had four to six times higher odds of disorder for all domains, compared with women who did not have cancer (39) (Supplementary Table 6, available online).

### Other Outcomes: Bipolar Disorder, Obsessive-Compulsive Problems, Stress-Related and Posttraumatic Stress, Sleep Disturbance, and Somatization

Other outcomes were infrequently studied, but five of five studies of sleep disturbance found a statistically significantly higher prevalence in breast cancer survivors, as did two of three studies of stress-related disorders, two of two studies of somatization, and the single studies identified with bipolar disorder and obsessive-compulsive outcomes (Table 2).

### Quality of the Studies

**Figure 4.**
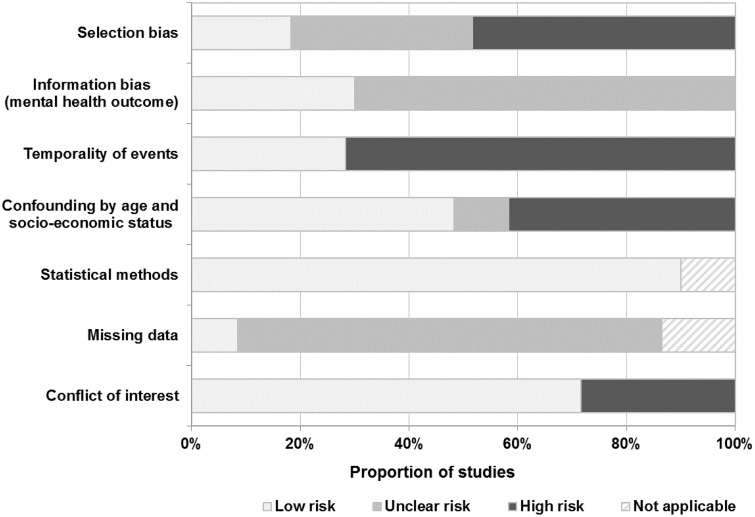
Summary of the risk of bias in the studies included in the systematic review. The risk of bias in statistical methods was considered not applicable when formal statistical comparisons between the two groups were not presented in the original study. Missing data criteria were not applicable for studies involving electronic health records.

Approximately 50% of the studies were rated at high risk of selection bias, mostly because of the nonprobabilistic recruitment of participants (eg, fliers and advertisements [28,31,44,47,56,57]) and the low proportion of women who accepted to participate in the studies (30,45,50,53,54) (Figure 4). In most studies (>70%), the risk of information bias was unclear, and the cross-sectional design precluded the unequivocal assertion that the onset of the mental disorder was posterior to the breast cancer diagnosis. Approximately 40% of studies reported results likely to have been affected by confounding by age and socio-economic status, and strategies to handle missing data were seldom reported. Individual study ratings are provided in Table 2.

## Discussion

Anxiety, depression, neurocognitive dysfunction, sexual dysfunction, and suicide appear to be more common in breast cancer survivors compared with noncancer groups. Scarcer data were available for other adverse mental health outcomes, but they were also reported as increased among breast cancer survivors. Common limitations of the current available evidence include use of nonprobabilistic samples, cross-sectional study designs making temporality of events difficult to assess, lack of power, and lack of consideration for important confounders such as socio-economic status.

Strengths of this review include the extensive search of multiple databases, the duplicated screening of the references and data extraction, and the systematic evaluation of the quality of the studies. The restriction to studies involving nonhospitalized samples and the inclusion of studies with in situ tumors allowed for a more generalizable characterization of the long-term burden of mental disorders in women in the community who have had breast cancer. We aimed to reduce the potential for information bias in the outcomes by considering only studies in which outcomes were assessed clinically or with validated instruments. However, this review also has limitations. Studies that reported mood assessments as secondary outcomes may not have been identified in the searches of electronic publication databases if the mental health outcome was not mentioned in the title, abstract, keywords, or indexing terms. This problem should have been minimized by our use of the four largest and most relevant databases in this field, supplemented by manual searches of all reference lists to further reduce the chances of major studies being missed. The comparability of clinically diagnosed outcomes over time may be limited by the changes in the diagnostic criteria, especially in cases such as sexual dysfunction where the criteria became narrower over time (87). We defined explicit criteria to evaluate the risk of bias in the studies, but our assessment may have been affected by the quality of the reporting of the original studies. We considered that confounding by age and socio-economic status had been accounted for when the studies matched participants for these factors, even though we acknowledge that matching per se may not completely remove the confounding effect (88).

The population-based studies included in this review consistently described more depression and anxiety in breast cancer survivors compared with the general population when these outcomes were clinically assessed. The group of breast cancer patients who receive a psychiatric diagnosis or who contact clinical services in relation to their mental health are likely to represent the most severe cases only; these patients are likely to benefit from medical treatment. Studies using receipt of antidepressants and anxiolytics prescriptions to define depression and anxiety, respectively, are likely to capture the specific group of patients who were thought to benefit from pharmacological intervention, which is only a subset of all patients with anxiety and depression. The indication of these drugs was not explored in any of the original studies, and misclassification of the outcome may have occurred because some of these drugs have other indications and are routinely used to manage vasomotor symptoms secondary to breast cancer treatments (89,90). In addition, we cannot rule out that patients with breast cancer history may have been more likely to be diagnosed with a mental health outcome due to increased contact with the health services compared with participants who did not have cancer.

The results from the original studies involving self-assessment scales, especially to assess symptoms of anxiety and depression, need to be interpreted with caution. These were often small, low-powered, cross-sectional studies using nonprobabilistic samples. Several of the original studies excluded women with psychiatric conditions and relied on voluntary participation. This may have resulted in an overrepresentation of psychologically healthier women, because diseased people are less likely to volunteer to participate in epidemiological studies (91,92); it is unclear if this would be differential between breast cancer survivors and control groups. The clinical profile of the patents included in these studies may also have been more favorable, because 45% of the studies included only patients with no recurrence and who were disease free at recruitment. In addition, misclassification of the outcome may have occurred, because these scales are screening tools and not suitable to establish definitive diagnoses. For example, the Hospital Anxiety and Depression Scale had only 50% sensitivity as a screening test for major depressive disorder in breast cancer survivors compared with the Structured Clinical Interview for the Diagnostic and Statistical Manual of Mental Disorders (93). Despite these limitations, scales are widely used in psychiatric epidemiology and in psycho-oncology research, and their results in this review are helpful to show the consistency of the results across methods of assessment.

For all methods of outcome definition, selective reporting in the original studies cannot be ruled out. Information on missing data was rarely well reported, and there was limited adjustment for potentially important confounders such as age and socio-economic status; residual confounding is still likely to be present in the studies that adjusted for education only.

Clinically relevant symptoms of anxiety and stress-related/adjustment disorders are common shortly after diagnosis (94), which is an expected response to a stressor that may be perceived as life-threatening and considering the uncertainty about the future that women may feel at this point (95). Declining trajectories of anxiety suggest that most women adjust to the diagnosis over time (96), but clinically relevant symptoms may persist in subgroups of women. Evidence on long-term trajectories of outcomes is scarce and needs to be further explored. Reported determinants of anxiety included younger age at diagnosis and having comorbidities; this is consistent with literature reporting that young breast cancer survivors have specific concerns, for example, fertility issues for women who want more children or weight gain during and after treatments (10). The increased symptoms of posttraumatic stress is consistent with a meta-analysis reporting that 10% of breast cancer survivors have posttraumatic stress disorder (97). Results for somatic and obsessive-compulsive symptoms must be interpreted with caution because they come from a small number of studies.

The increased frequency of depression in breast cancer survivors is plausible considering that many report unmet needs in several domains that affect quality of life (98), including impact on relationships, lifestyle changes induced by the cancer, lack of psychological support, and difficulties obtaining understandable information about the physical long-term effects of the treatments (99–101). Risk factors for depression in breast cancer patients appear to be similar to those for the general female population, including less social support and lower socio-economic status (46). Suicide almost always occurs among people suffering from a mental health disorder, most often depression (102,103). The increased risk of suicide in breast cancer survivors is likely to be underestimated, because suicide is often classified under other causes of death, and this may happen more often in women who have had cancer.

Neurocognitive dysfunction, also known as chemo-fog, has been linked to the neurotoxic effects of chemotherapy (104). Other determinants of neurocognitive dysfunction recently postulated include posttraumatic stress disorder (59) and exposure to hormone therapy due the effects of estrogen deprivation in the neuronal structures (82). Impairments for one or more domains of neurocognitive function (eg, memory [65,83] and processing speed [77,81]) were often described, but the methodological heterogeneity of the studies (105) as well as the challenge to measure neurocognitive function (106) hamper comparisons, and it is currently debatable which specific domains are impaired.

The narrow inclusion/exclusion criteria in some eligible studies of sexual dysfunction preclude generalizability to the general population of breast cancer survivors. For example, Safarinejad et al. (39) excluded women who did not attempt sexual intercourse weekly and Boehmer et al. (47) included only in lesbian or bisexual women. The aetiology of sexual dysfunction in women with a history of breast cancer is thought to be multifactorial. Vaginal dryness is a common iatrogenic effect of hormone therapy or chemotherapy-induced ovarian failure and may lead to dyspareunia (14). However, impaired sexual function, compared with healthy women, has also been reported in women treated with surgery only (48), indicating that factors other than the physical ones may be involved. Indeed, the distress in partnered relationships (107–110), body image concerns (111,112), depressive feelings (113), younger age at diagnosis (113), and presence of comorbidities (114) have all been reported amongst the most important determinants of female sexual dysfunction.

Mitchell et al. (115) systematically reviewed studies providing data for depression and anxiety in survivors from several types of cancer (>2 years since diagnosis) and in healthy subjects. The results indicated that anxiety, but not depression, may be increased among cancer survivors (115). This conclusion arose from the meta-analysis of nine studies that provided data for anxiety and included patients diagnosed with breast, colorectal, prostate, testicular, and cervical cancers or Hodgkin’s lymphomas as well as patients diagnosed with cancers during adolescence and young adulthood. It is currently unknown if, and how, the risk of anxiety and depression varies by cancer type, and thus we cannot directly compare our results. Other systematic reviews on the topic assessed the prevalence of anxiety and depressive symptoms in cancer survivors (11,116–118), including studies without a comparison group. Maass et al. (11) described a higher frequency of depressive symptoms among breast cancer survivors (>1 year since diagnosis) compared with normative data found in the literature. The results for cognitive dysfunction are in accordance with those reported by Jim et al. (119), who found small but increased cognitive deficits in breast cancer survivors treated with chemotherapy compared with noncancer and cancer controls.

Several studies have reported no differences in most domains of health-related quality of life (HRQoL) between long-term breast cancer survivors and women in the general population (120–122). The interpretation of our results in the context of the literature for HRQoL is not straightforward, and the apparent difference is likely to be explained by the combination of several factors, including the differential participation of psychologically healthier women in HRQoL surveys and positive effects of surviving breast cancer. Patients with adverse mental health outcomes, especially those with the most severe categories, may be less likely to participate in HRQoL surveys. This contrasts with the studies in this review that included women with a clinical diagnosis and/or treated for a mental health disorder and were thus likely to capture the most severe cases. In addition, long-term breast cancer survivors report changes in several aspects of their lives, but not all of them are negative. Women in the survivorship period have described feeling improved empathy, closer relationships, and a greater appreciation for life (123). This phenomenon of heightened well-being after a stressful event—known as posttraumatic stress growth—has been described to affect up to 60% of breast cancer survivors (124). Quality of life reflects how women perceive their current status, and the occurrence of posttraumatic growth may offset some of the negative feelings associated with breast cancer (125). In addition, studies of HRQoL often reported mean scores of overall and domain-specific measures of HRQoL; subgroups that have a different trajectory of symptoms can be hard to disentangle based on standard analyses.

This study has several implications for clinical practice. It is important to raise awareness amongst health care professionals acting at various levels of the health care system of the increased risk of mental health symptoms among breast cancer survivors, in particular anxiety, depression, and neurocognitive and sexual dysfunctions. Screening for mental health disorders in some or all of the breast cancer survivor population may be warranted. Predictors of distress among breast cancer survivors include having perceived functioning limitations, fatigue, younger age, lower socioeconomic status, and psychiatric history, and modifiable factors such as vasomotor symptoms, pain, less social support, physical activity, and cigarette smoking (126). As such, screening for anxiety and depression may be especially relevant for younger patients, and all those within the first few years of survivorship, with co-morbidities, living alone, or diagnosed with more advanced disease; patients with depression should be assessed for suicidal ideation. Patients who experienced treatment-induced menopause are likely to benefit from being asked about their sexual function, because they may avoid this topic with their clinicians; patients who received chemotherapy may also benefit from assessment for clinically significant cognitive impairments. Psychosocial support and routine monitoring of patient-reported outcomes during survivorship care are likely to help reduce the burden of these conditions. Differentiated psychological services are becoming the norm in specialized breast cancer clinics; however, only a fraction of the breast cancer survivors are followed-up in these settings (127). The holistic approach to the patients’ unmet needs also requires equipping health care professionals with evidence-based information on the optimal management strategies. For example, treatment for sexual dysfunction may require not only management of anxiety and depressive symptoms, but also vaginal dryness, which may be undertreated in women with history of estrogen-receptor positive breast cancer due to concerns over the effect of hormonal vaginal treatments (128) and unawareness of the recommendations for lubricants and moisturizers (129). Patients’ education on common changes post breast cancer, and the strategies available to manage these, may help women to better understand and cope with their disease, increase patients’ awareness of common symptomatology, and help to decrease the stigma associated with mental health disorders.

Our review also identified areas for further research. There is a pressing need for studies evaluating clinically diagnosed adverse mental health outcomes in samples of women likely to represent the cohort of survivors in the general population and with sufficient numbers to allow effects to be detected. Further research is particularly needed to better characterize the trajectories of mental health outcomes over time, particularly of anxiety, depression, and neurocognitive dysfunction. The long-term risk of sleep disorders needs clarification, because breast cancer treatments such as chemotherapy and steroids have been suggested to be associated with impaired sleep (130,131), possibly due to increased risk of vasomotor symptoms that affect the sleep quality and quantity (132). Evidence on the long-term effect of being diagnosed in situ vs invasive tumors and on having undergone breast reconstructive surgery is scarce despite the increasing numbers of ductal carcinoma in situ diagnoses and aesthetics surgeries performed. The role of systemic treatments other than chemotherapy on neurocognitive function also needs clarification, including the role of the different types of hormonal treatments (selective oestrogen receptors modulators vs aromatase inhibitors). Efforts should be made to employ standardized definitions of the outcomes, because the heterogeneity of diagnostic codes and psychometric instruments hampers comparability of results across studies. Further research is also needed on the performance of commonly used scales for anxiety and depression as screening tools for these conditions in breast cancer survivors. Studies should also consider that the incidence of mental health disorders after a breast cancer diagnosis may vary with age, socio-economic status, time, stage of disease, recurrence, type of treatment, and sequelae from cancer among other factors. The inclusion of a comparison group is essential to estimate the excess risk of the breast cancer survivorship.

In conclusion, women with a history of breast cancer appear to be at higher risk of a wide range of adverse mental health outcomes up to several years post diagnosis and treatment compared with women who did not have cancer. The evidence was particularly compelling for anxiety, depression and neurocognitive and sexual dysfunctions, and suicide, which were most often studied. However, there is a pressing need for more population-based research to better characterize the association between breast cancer history and mental health. Our results can be used to inform prevention and management strategies directed at tackling the burden of adverse mental health outcomes in breast cancer survivors.

## Funding

This work was supported by the Medical Research Council (MRC) and the Clinical Practice Research Datalink (CPRD) at the Medicines and Healthcare products Regulatory Agency (MHRA) (grant no. MR/M016234/1 to HC); the Bangerter Foundation and the Swiss Academy of Medical Sciences (grant no. TCR 14/17 to MM); and the Wellcome Trust and the Royal Society (grant no. 107731/Z/15/Z to KB).

## Notes

Department of Non-Communicable Disease Epidemiology, Faculty of Epidemiology and Population Health, London School of Hygiene & Tropical Medicine, London, UK (HC); Clinical Practice Research Datalink (CPRD), Medicines and Healthcare Products Regulatory Agency, London, UK (RW); Department of Emergency Medicine, Inselspital, Bern University Hospital, University of Bern, Bern, Switzerland (MM); Institute of Health Economics and Clinical Epidemiology, University Hospital of Cologne, Cologne, Germany (MM); Department of Non-Communicable Disease Epidemiology, Faculty of Epidemiology and Population Health, London School of Hygiene & Tropical Medicine, London, UK (RH); Department of Medicine, The Royal Marsden NHS Foundation Trust, London and Surrey, UK (SS); Department of Non-Communicable Disease Epidemiology, Faculty of Epidemiology and Population Health, London School of Hygiene & Tropical Medicine, London, UK (KB).The funders had no role in the design of the study; the collection, analysis, and interpretation of the data; the writing of the manuscript; and the decision to submit the manuscript for publication.

Conflict of interest: Ms Williams reports that CPRD has financial relationships with its clients, including the London School of Hygiene and Tropical Medicine, in relation to providing access to research data and services outside the submitted work. Dr Stanway reports personal fees from Roche, Clinigen, Eli Lilly, and Novartis not related to this work. Dr Bhaskaran reports grants from Wellcome Trust, the Royal Society, Medical Research Council, and British Heart Foundation outside the submitted work. The other authors in this report have no conflict of interest to disclose.

Acknowledgments: We thank Dr Maja Nikšić and Ms Ruoran Li for their help in interpreting the papers written in Serbian and Chinese, respectively.

## Supplementary Material

Supplementary Data
