# Supplementary material for: Associations Between Breast Cancer Survivorship and Adverse Mental Health Outcomes: A Systematic Review
Source: J Natl Cancer Inst. 2018 Nov 7;110(12):1311–27. doi: 10.1093/jnci/djy177 (PMC6292797; doi:10.1093/jnci/djy177)
Supplement: Supplementary Data [file djy177_supp.pdf]

Supplementary Materials

Supplementary Table 1. MEDLINE search expression in OVID®.

#	Search
1	exp Breast Neoplasms/
2	(breast and (cancer* or carcinoma* or tumo?r* or neoplas*)).mp. [mp=title, abstract, original title, name of substance word, subject heading word, keyword heading word, protocol supplementary concept word, rare disease supplementary concept word, unique identifier]
3	1 or 2
4	exp catatonia/ or exp depression/ or exp self-injurious behavior/ or exp anxiety/
5	mental disorders/ or exp anxiety disorders/ or exp "bipolar and related disorders"/ or exp "disruptive, impulse control, and conduct disorders"/ or exp dissociative disorders/ or "feeding and eating disorders"/ or anorexia nervosa/ or binge-eating disorder/ or bulimia nervosa/ or pica/ or exp mood disorders/ or exp motor disorders/ or neurocognitive disorders/ or amnesia/ or cognition disorders/ or auditory perceptual disorders/ or mild cognitive impairment/ or consciousness disorders/ or delirium/ or dementia/ or exp neurotic disorders/ or exp personality disorders/ or exp "schizophrenia spectrum and other psychotic disorders"/ or sexual dysfunctions, psychological/ or exp sleep wake disorders/ or exp somatoform disorders/ or exp substance-related disorders/ or exp "trauma and stressor related disorders"/
6	(depressi* or dysthymia or catatonia or self-injur* or self-injury or self-injurious or self-mutilation or "self mutilation" or suicid* or self-harm or "self harm" or "self injury" or anxious* or anxiety or (panic adj1 (disorder# or attack#)) or catastrophi* or (mental adj1 (disorder or disorders)) or phobia or phobic or neurotic or (compulsive adj1 disorder) or bipolar or neurotic or (personality adj1 disorder) or psychotic or psychosis or paranoid or delusional or (sexual adj1 (disorder or dysfunction or problem#)) or insomnias or (sleep adj1 (disorder or dysfunction or problem#)) or somatoform or (substance adj3 (disorder or problem#)) or stress ajd3 disorder or (adjustment adj3 disorder)).mp. [mp=title, abstract, original title, name of substance word, subject heading word, keyword heading word, protocol supplementary concept word, rare disease supplementary concept word, unique identifier]
7	4 or 5 or 6
8	(prevalence# or frequenc* or incidence# or risk or rate* or ratio or odds or epidemiolog* or percent* or outcomes or hazard).mp. [mp=title, abstract, original title, name of substance word, subject heading word, keyword heading word, protocol supplementary concept word, rare disease supplementary concept word, unique identifier]
9	3 and 7 and 8
10	Humans/
11	Animals/
12	10 and 11
13	11 not 12
14	9 not 13

Supplementary Table 2. Criteria used to judge the risk of bias in the systematic review studies.

Judgment	Selection bias	Outcome variable: information bias	Design-specific source of bias (temporality)	Confounding by age and socio-economic status	Statistical methods	Missing data	Conflict of interest
**Low risk of bias**	Describes the source and methods of selection of the participants<br>AND<br>Eligibility criteria given<br>AND<br>(Participants selected at random OR population-based study)<br>AND<br>Proportion of participation >50%<br>AND/OR<br>≤30% of attrition (for cohort studies with a pre-defined follow up time for the entire cohort)	Outcome assessed through one of the following:<br>Psychiatric interviews<br>OR<br>Evidence of having been prescribed anxiolytics (for anxiety) and antidepressants (for depression)<br>OR<br>Record of a diagnostic code for mental health (for studies including electronic health records)<br>OR<br>Country's official mortality registry data (for completed suicide)<br>OR<br>Objective data on the trajectories of cognitive function over time (for neurocognitive dysfunction)	The breast cancer diagnosis preceded the onset of the mental health outcome<br>OR<br>Diagnosis of the relevant outcome prior to the BC diagnosis taken into account by restriction, matching or in multivariate analysis	The study attempts to minimise confounding using one or more of the following:<br><br>Matching for age and for an indicator of socio-economic status (e.g. education, attending the same primary care practice, or small geographic area)<br>AND/OR<br>Multivariate analysis, reporting mean scores or association measures, adjusted for age and a socio-economic status indicator	Appropriate use of statistics for primary analysis of effect (specific to each study design and data)	≤15% of missing data (for studies with questionnaires), with or without multiple imputation methods for missing data<br>OR<br>>15% of missing data, with missing data imputed using multiple imputation methods	The study authors explicitly report the existence, or not, of conflicts of interests<br>OR<br>The study's funding source is acknowledged
**High risk of bias**	Participants not selected at random<br>OR<br>Proportion of participation ≤50%<br>OR<br>Women selected on the basis of a the relevant mental health outcome for this review<br>OR<br>>30% of attrition (for cohort studies with a pre-defined follow up time for the entire cohort)	Self-reported intake of anxiolytics (for anxiety) OR antidepressants (for depression)	Unclear whether the onset of the mental health outcome occurred before or after the breast cancer diagnosis<br>OR<br>Diagnoses of mental disorders before the onset of the BC not considered	The study only reports crude measures of frequency or association (e.g. univariate association, or mean scores of the instrument)<br>OR<br>(There are differences between the two the group of breast cancer survivors and the women in the comparison group for age OR for an indicator of socio-economic status)	Not appropriate use of statistics for primary analysis of effect	>15% of missing data (for studies using questionnaires), with missing data imputed with a measure of central tendency	The presence or absence of conflicts of interest is not reported and thus unknown<br>AND<br>No study's funding source is acknowledged
**Unclear risk of bias**	Unknown method of participants' recruitment<br>OR<br>Unknown exclusion criteria<br>OR<br>Unknown participation rate	Outcome assessed using self-reported scales	Not applicable	The study reports mean scores or measured of associations that were adjusted for an unclear or unknown list of potential confounders	Statistical methods not reported	Proportion of missing data not reported (for studies involving questionnaires)<br><br>Not applicable if the study uses data from diagnoses ascertained via electronic records, or if formal statistical comparisons between breast cancer survivors and women who did not have cancer could not be done.	Not applicable

Supplementary Table 3. Anxiety: main characteristics and results of the studies that compared the risk, prevalence or severity of anxiety (disorders or symptoms) between breast cancer survivors (>1 year) and women who did not have cancer.

**First author, year of publication**<br><br>**Country**	**Breast cancer survivors**				**Comparison group**	**Outcome assessment**	**Quantitative measure of the outcome**		**Relative risk estimate (RR, OR, SIR, PR)**	**P-value or 95% confidence interval**	**Notes**
	**Type of population and main characteristics**	**Stage at diagnosis (%)**	**Breast cancer treatments (%)**	**Time since diagnosis/ treatment in years: mean/ median (SD), range**	**Type of population and main characteristics**		**Breast cancer survivors**	**Comparison group**			
Electronic health records											
Hjerl et al., 2002 [1]<br><br>Denmark<br><br>(continues)	Population-based<br><br>All 60,431 women aged >15 years with a first primary invasive breast cancer registered in the national Cancer Registry in 1970-1993.	All	ND	4 (ND), 0-15<br><br>(Median cohort follow up: 4 years since diagnosis; range: 0 to 15)	Population-based<br><br>Danish female population aged >15 years.	EHR, first ever psychiatric admission, as registered in the Danish Psychiatric Central Registry<br><br>ICD-8 codes: 300.81 and 300.00-300.99, except 300.49	Cumulative incidence: 0.25%	Cumulative incidence: 0.20%	SIR= 1.3 *	95%CI: 1.1-1.5	Standardised incidence ratio estimated considering all follow up time since diagnosis.
									By age:<br>30-34: SIR= 1.93<br>35-39: SIR= 1.28<br>40-44: SIR= 0.91<br>45-49: SIR= 0.89<br>50-54: SIR= 1.24<br>55-59: SIR= 1.56 *<br>60-64: SIR= 1.18<br>65-69: SIR= 1.42<br>70-74: SIR= 1.98 *<br>75-79: SIR= 0.47<br>80-84: SIR= 1.27<br>85-89: SIR= 2.91<br>≥90: SIR= 8.74	By age:<br>95%CI:0.69-4.15<br>95%CI:0.58-2.38<br>95%CI:0.48-1.52<br>95%CI:0.54-1.37<br>95%CI:0.84-1.76<br>95%CI:1.04-2.22<br>95%CI:0.69-1.86<br>95%CI:0.81-2.26<br>95%CI:1.12-3.21<br>95%CI:0.08-1.46<br>95%CI:0.21-3.91<br>95%CI:0.17-12.8<br>95%CI:0.50-38.5	
									By calendar period:<br>1970-74: SIR=1.11<br>1975-79: SIR=1.15<br>1980-84: SIR=1.04<br>1985-89: SIR=1.80 *<br>1990-93: SIR=0.89	By calendar period:<br>95%CI:0.58-1.91<br>95%CI:0.78-1.61<br>95%CI:0.72-1.45<br>95%CI:1.37-2.31<br>95%CI:0.55-1.35	-
	Women aged >15 years with first invasive breast cancer registered in the national Cancer Registry in 1970-1993 and living outside Copenhagen city area (non-metropolitan).	All	ND	4 (ND), 0-15	Female population aged >15 years and living outside Copenhagen city area (non-metropolitan).	EHR, first ever psychiatric admission, as registered in the Danish Psychiatric Central Registry<br><br>ICD-8 codes: 300.81 and 300.00-300.99, except 300.49	-	-	SIR= 1.3 *	95%CI: 1.1-1.5	Standardised incidence ratio estimated considering all follow up time since diagnosis.
				1.5			-	-	SIR= 1.4	95%CI: 0.8-2.1	Approximate SIR values estimated from the graphics provided in the original study.
				2.5			-	-	SIR= 1.1	95%CI: 0.6-1.8	
				3.5			-	-	SIR= 1.6	95%CI: 0.9-2.5	
				4.5			-	-	SIR= 1.5	95%CI: 0.6-2.4	
				5.5			-	-	SIR= 0.7	95%CI: 0.3-1.6	
				6.5			-	-	SIR= 1.3	95%CI: 0.5-2.6	
				7.5			-	-	SIR= 1.2	95%CI: 0.4-2.5	
				8.5			-	-	SIR= 0.8	95%CI: 0.3-2.2	
				9.5			-	-	SIR= 0.7	95%CI: 0.2-2.1	
				10.5			-	-	SIR= 0.4	95%CI: 0.1-1.9	
				11.5			-	-	SIR= 1.0	95%CI: 0.3-2.9	
				12.5			-	-	SIR= 2.6	95%CI: 0.8-6.0	
				13.5			-	-	SIR= 0.5	95%CI: 0.1-2.1	

Hjerl et al., 2002 [1]<br><br>Denmark<br><br>(continued)	Women aged >15 years with first invasive breast cancer registered in the national Cancer Registry in 1970-1993 and living in Copenhagen city area (metropolitan).	All	ND	4 (ND), 0-15	Female population aged >15 years and living in Copenhagen city area (metropolitan).	EHR, first ever psychiatric admission, as registered in the Danish Psychiatric Central Registry<br><br>ICD-8 codes: 300.81 and 300.00-300.99, except 300.49	-	-	SIR= 1.1	95%CI: 0.8-1.6	Standardised incidence ratio estimated considering all follow up time since diagnosis.
				1.5			-	-	SIR= 1.4	95%CI: 0.5-2.5	Approximate values estimated from the graphics provided in the original study.
				2.5			-	-	SIR= 1.5	95%CI: 0.4-3.0	
				3.5			-	-	SIR= 0.7	95%CI: 0.2-2.2	
				5.0			-	-	SIR= 0.8	95%CI: 0.3-1.8	
				6.5			-	-	SIR= 1.3	95%CI: 0.4-4.0	
				7.5			-	-	SIR= 3.3	95%CI: 1.0-7.6	
				9.5			-	-	SIR= 0.5	95%CI: 0.1-1.8	
				13.0			-	-	SIR= 0.7	95%CI: 0.1-2.9	
Hung et al., 2013 [2]<br><br>Taiwan	Population-based<br><br>26,629 women with no prior mood disorder and cancer, with breast cancer registered in the National Health Insurance Database in 2000-2005.	All	ND	2.7 (ND), ND-7<br>(median follow up years for breast cancer survivors: 2.7; for matched cohort: 3.2)	Population-based<br>26,629 women randomly selected from 1 million women who did not have breast cancer registered in the same database, individually matched for age and Charlson comorbidity score (categories of matching not reported).	EHR, recorded in the Registry for Catastrophic Illness with an ICD-9-CM code for anxiety (300-300.3, 300.5, 300.7-300.9)	Incidence rate: 49.64 per 1,000 person-years<br><br>Cumulative incidence: 15%	Incidence rate: 40.82 per 1,000 person-years<br><br>Cumulative incidence: 14%	RR= 1.22 *	95%CI: 1.16-1.27	Includes patients diagnosed with breast cancer at <1yr.
				2			Cumulative incidence: 11%	Cumulative incidence: 9%	RR= 1.22 * †	95%CI: 1.16-1.29	Approximate cumulative incidence values estimated from the graphics provided in the original study.
				4			17%	15%	RR= 1.13 * †	95%CI: 1.09-1.18	
				6			22%	20%	RR= 1.10 * †	95%CI: 1.06-1.14	
Khan et al., 2010 [3]<br><br>United Kingdom	Population-based<br><br>16,938 women aged ≥30 with breast cancer registered in the UK General Practice Research Database.	All	ND	ND (ND), ≥5	Population-based<br><br>67,649 women who did not have breast or colorectal cancer at beginning of follow up; individually matched for age (± 1 year) and primary care practice (small area).	EHR, having primary care consultations for anxiety	Prevalence: 5.4%	Prevalence: 5.0%	OR= 1.06	95%CI: 0.97-1.16	Odds ratio adjusted for Charlson comorbidity score, previous history of anxiety and death.
						EHR, being prescribed an anxiolytic at least once	Prevalence: 9.0%	Prevalence: 7.7%	OR= 1.08 *	95%CI: 1.01-1.15	Odds ratio adjusted for Charlson comorbidity score, number of consultations, and death.
Yang et al., 2017 [4]<br><br>Sweden<br><br>(continues)	Population based<br><br>All 4,402 women diagnosed with an in situ breast cancer at the age of 20-80 years between 2001-2009	0	ND	4.7 (4.4), 0-10<br><br>(median (IQR) duration of follow up: 4.7 (4.4))	Population based<br><br>452,507 women randomly selected from the respondents to the 1990 census	EHR, ICD-10 diagnostic codes for anxiety (F40-F41) at in patient or outpatient hospital visits	Cumulative incidence: 0.9%	Cumulative incidence: 0.9%	SIR= 0.99<br>By age group:<br>20-44: SIR= 1.18<br>45-54: SIR= 0.97<br>55-64: SIR= 0.95<br>65-80: SIR= 0.91	95%CI: 0.73-1.34<br>By age group:<br>95%CI: 0.59-2.36<br>95%CI: 0.57-1.64<br>95%CI: 0.53-1.72<br>95%CI: 0.45-1.81	Standardised incidence ratio estimated considering all follow up time since diagnosis.
				0-0.5			<0.1%	0.1%	SIR= 0.53	95%CI: 0.13-2.12	Standardised incidence ratios were standardised by calendar period (1-year categories), age (5-year categories), and region of residence (North, Stockholm-Gotland, South, Southeast, Uppsala-Orebro, West).
				0.5-1			0.0%	0.0%	-	-	
				1-2			0.3%	0.2%	SIR= 1.62	95%CI: 0.92-2.85	
				2-5			0.4%	0.4%	SIR= 1.09	95%CI: 0.68-1.73	
				5-10			0.2%	0.2%	SIR= 0.90	95%CI: 0.47-1.74	

Yang et al., 2017 [4]<br><br>Sweden<br><br>(continued)	Population based<br><br>All 4,402 women diagnosed with an in situ breast cancer at the age of 20-80 years between 2001-2009	0	ND	4.7 (4.4), 0-10<br><br>(median (IQR) duration of follow up: 4.7 (4.4))	452,507 women randomly selected from the respondents to the 1990 census	EHR, being prescribed an anxiolytic (group N05B of the ATC classification system)	Cumulative incidence: 4.5%	Cumulative incidence: 2.8%	SIR= 1.64 *<br>By age group:<br>20-44: SIR= 1.52<br>45-54: SIR= 1.69 *<br>55-64: SIR= 1.57 *<br>65-80: SIR= 1.69 *	95%CI: 1.43-1.88<br>By age:<br>95%CI: 0.96-2.42<br>95%CI: 1.28-2.22<br>95%CI: 1.22-2.02<br>95%CI: 1.34-2.14	Standardised incidence ratio estimated considering all follow up time since diagnosis.<br><br>Standardised incidence ratios were standardised by calendar period (1-year categories), age (5-year categories), and region of residence (North, Stockholm-Gotland, South, Southeast, Uppsala-Orebro, West).
				0-0.5			-	-	SIR= 3.86 *	95%CI: 3.17-4.71	
				0.5-1			-	-	SIR= 0.93	95%CI: 0.61-1.41	
				1-2			-	-	SIR= 1.28	95%CI: 0.97-1.70	
				2-4.5			-	-	SIR= 0.91	95%CI: 0.64-1.28	
	Population based<br><br>All 40,849 women diagnosed with an invasive breast cancer at the age of 20-80 years between 2001-2009	I-IV	ND	4.5 (4.5), 0-10<br><br>(median (IQR) duration of follow up: 4.4 (4.5))	Population based<br><br>452,507 women randomly selected from the respondents to the 1990 census	EHR, ICD-10 diagnostic codes for anxiety (F40-F41) at in patient or outpatient hospital visits	Cumulative incidence: 1.4%	Cumulative incidence: 0.9%	SIR= 1.55 *<br>By age group:<br>20-44: SIR= 1.84 *<br>45-54: SIR= 1.56 *<br>55-64: SIR= 1.58 *<br>65-80: SIR= 1.31 *	95%CI: 1.43-1.68<br>By age group:<br>95%CI: 1.54-2.21<br>95%CI: 1.34-1.81<br>95%CI: 1.35-1.84<br>95%CI: 1.10-1.56	The following were significant predictors of increased anxiety among breast cancer survivors: younger age at diagnosis, presence of co-morbidities, having moderate and high histological grade, and having had chemotherapy.
				0-0.5			0.2%	0.1%	SIR= 2.53 *	95%CI: 2.05-3.13	
				0.5-1			0.2%	0.1%	SIR= 2.30 *	95%CI: 1.85-2.87	
				1-2			0.3%	0.2%	SIR= 2.00 *	95%CI: 1.69-2.38	
				2-5			0.4%	0.4%	SIR= 1.17 *	95%CI: 1.01-1.36	
				5-10			0.3%	0.2%	SIR= 1.18	95%CI: 0.97-1.42	
	Population based<br><br>All 40,849 women diagnosed with an invasive breast cancer at the age of 20-80 years between 2001-2009	I-IV	ND	4.5 (4.5), 0-10<br><br>(median (IQR) duration of follow up: 4.4 (4.5))	Population based<br><br>452,507 women randomly selected from the respondents to the 1990 census	EHR, being prescribed an anxiolytic (group N05B of the ATC classification system)	Cumulative incidence: 6.4%	Cumulative incidence: 2.5%	SIR= 2.52 *<br>By age group:<br>20-44: SIR= 3.96 *<br>45-54: SIR= 3.04 *<br>55-64: SIR= 2.50 *<br>65-80: SIR= 2.04 *	95%CI: 2.43-2.62<br>By age group:<br>95%CI: 3.56-4.40<br>95%CI: 2.81-3.30<br>95%CI: 2.33-2.68<br>95%CI: 1.91-2.17	
				0-0.5			-	-	SIR= 6.13 *	95%CI: 5.81-6.47	
				0.5-1			-	-	SIR= 1.90 *	95%CI: 1.72-2.10	
				1-2			-	-	SIR= 1.47 *	95%CI: 1.35-1.61	
				2-4.5			-	-	SIR= 1.38 *	95%CI: 1.26-1.52	
**Studies involving scales**											
Cohen et al., 2011 [5]<br><br>Israel	Convenience sample<br><br>56 married Israeli Arab breast cancer survivors, post treatment and free of disease recruited from one hospital.	I-III (ND%)	Srg, C: 48.2%<br>Srg, M: 51.8%<br>Srg, R: 12.5%<br>CT: 85.7%<br>RT: 85.7%<br>HT: 58.9%	4.8 (4.2), 1-17	Convenience sample<br>66 married and 'healthy' Arab women living in Israel, approached in community settings; individually matched for age and education (matching categories not reported).	BSI-18	BSI-18 mean score (SD):<br><br>2.7 (1.2)	BSI-18 mean score (SD):<br><br>2.2 (0.9)	-	P<0.05 *	Higher levels of anxiety associated with higher levels of depression, somatization and emotional distress in both groups (P<0.001).<br><br>Higher levels of anxiety associated with lower body image in breast cancer survivors only (P=0.05).

Boehmer et al., 2015 [6]<br><br>ND	Convenience sample<br><br>85 lesbian or bisexual breast cancer survivors post-active treatment recruited via advertisements, flyers, etc. (3.5% of whom had had cancer recurrence).	I-III (100%)	ND	4.5 (ND), 1-10	Convenience sample<br><br>85 lesbian or bisexual women with no history of cancer, not using hormone therapy, recruited via flyers, advertisements, etc.; individually matched for age (± 3 years) and partner status (partnered vs. unpartnered).	Anxiolytics intake (self-reported)	Prevalence: 3.5%	Prevalence: 1.2%	PR=2.92 †	95%CI: 0.31-27.1	Anxiety was more common in women taking any psycho pharmacological medication, compared to those who did not (OR=3.78, 95%CI: 1.76 to 8.09).
						HADS score ≥8	Prevalence: 45.2%	Prevalence: 36.5%	PR=1.24 †	95%CI: 0.86-1.78	
Calvio et al., 2010 [7]<br><br>United States	Convenience sample<br><br>122 breast cancer survivors, working full-time for ≥1 year, with computer and internet, recruited via advertisements and flyers.	I (36.9%)<br>II (44.3%)<br>III (17.2%)	Srg, ND: 96.7%<br>CT: 82.8%<br>RT: 73.0%<br>HT: 45.9%<br>IT: 13.1%	3.1 (2.4), 1-10	Convenience sample<br><br>113 women without cancer, working full-time for ≥1 year, with computer and internet, recruited via advertisements and flyers.	HADS	HADS mean score (SD):<br><br>7.8 (3.0)	HADS mean score (SD):<br><br>7.1 (2.6)	-	P<0.01 *	Higher HADS scores indicate more anxiety symptoms.<br><br>Mean scores adjusted for marital status (cohabitating with partner vs. single/not cohabitating), race (Caucasian vs. non-Caucasian), ethnicity (Hispanic vs. non-Hispanic), age (<40, 41-50, 51-65), income (0-39,000; 40-59,000; 60-79,000; 80-89,000; 80-99,000; ≥100,000), and menopausal status (currently going through, premenopausal, postmenopausal).
Dahl et al., 2011 [8]<br><br>Norway	Convenience sample<br><br>337 tumor free breast cancer survivors treated with radiotherapy during 1998 and 2002 in one hospital.	II (ND)<br>III (ND)	Srg, C: 24%<br>Srg, M: 76%<br>CT: 82%<br>RT: 100%<br>HT: 81%	3.9 (ND), 2.6-6.9	Convenience sample<br><br>1,685 women randomly selected from a population-based sample of women with no history of cancer who provided questionnaires with complete data; individually matched for age (± 5 years).	HADS	HADS mean score (SD):<br><br>6.3 (2.8)	HADS mean score (SD):<br><br>4.8 (3.7)	-	P<0.001 *	Higher HADS score indicates more anxiety symptoms.<br><br>Mean scores adjusted for level of education, on disability pension and menopausal status.<br><br>Higher scores of HADS for anxiety were associated with more insomnia symptoms in breast cancer survivors and in controls (p<0.001).
Miao et al., 2016 [9]<br><br>China	Convenience sample<br><br>23 patients with breast cancer who had been treated with chemotherapy at a local hospital.	I-III (100%)	CT: 100%	3 (0.3),	Convenience sample<br><br>26 age-matched healthy controls selected amongst patients relatives and local universities; matched for age (matching method not reported).	HRS-A	HRS-A mean score (SD):<br><br>4.96 (1.43)	HRS-A mean score (SD):<br><br>4.5 (1.22)	-	P=0.232	Higher HRS-A score indicates more anxiety symptoms.

Rubino et al., 2007 [10]<br>Italy	Convenience sample<br>33 consecutive patients who had had breast-reconstruction after mastectomy, in 2001-2002.	ND	Srg, M: 100%<br>Srg, R: 100%	ND (ND), >1	Convenience sample<br>33 'healthy' women, randomly selected amongst the personnel of the local university.	HRS-A, applied during psychiatric interview<br>Cut-off score: >14	Prevalence: 24.2%	Prevalence: 0.0%	PR=7.99 * †	95%CI: 1.06-60.34	PR calculated by the authors of the present study. For calculation purposes, it was assumed that one person in the non-cancer group had the outcome.
Boele et al., 2015 [11]<br>The Netherlands	Convenience sample<br>Post-menopausal breast cancer survivors with no diagnosis of psychiatric illness, not treated with adjuvant CT, selected from the medical records of the Cancer Institute.	ND	Srg, ND: 95%<br>CT: 0%<br>RT: 65%<br>HT: 100% / 0%	Exposure to HT: 3.2 (1.9), 1.5-7;<br>Unexposed to HT: 2.8 (0.3), 2.3-3.3.	Convenience sample<br>44 friends or family members of the women who had had breast cancer, with no history of breast cancer, matched for age and education (method of matching not reported).	HSCL-25	HSCL-25 mean score (SD):<br>HT: 11.17 (10.39)<br>No HT: 13.57 (11.74)	HSCL-25 mean score (SD):<br>9.92 (10.55)	-	P=0.30	Higher HSCL-25 score indicates more anxiety symptoms.<br>P adjusted for age and estimated premorbid IQ.<br>Women with higher anxiety levels had significantly lower processing speed evaluated as part of cognitive function.
Kreukels et al., 2008 [12]<br>The Netherlands	Convenience sample<br>63 women who had non-metastatic breast cancer, with no history of psychiatric diseases.	I-III (100%)	CT: 100%<br>HT: 40%	~ 1<br>(follow up at 12 months after CT)	Convenience sample<br>60 friends or family of the patients with the same age who never had cancer, matched for age (method of matching not reported).	HSCL-25	HSCL-25 mean score (SD):<br>16.3 (12.2)	HSCL-25 mean score (SD):<br>8.7 (7.9)	-	P<0.001 *	Higher HSCL-25 score indicates more anxiety symptoms.
Amir et al., 2002 [13]<br>Israel	Convenience sample<br>39 women free of cancer symptoms for ≥3 years and not under active treatment, identified through 2 hospitals.	I (46%)<br>II (46%)<br>III (8%)	Srg, C: 20%<br>Srg, M: 80%<br>CT: 66%<br>RT: 41%<br>HT: 46%	6.5 (ND), ≥5	Convenience sample<br>39 women who did not experience life-threatening disease, recruited by unknown methods, matched for age and education (method of matching not reported).	SCL-90	SCL-90 mean score (SD):<br>0.87 (0.96)	SCL-90 mean score (SD):<br>0.49 (0.35)	-	P<0.001 *	Higher SCL-90 scores indicate more anxiety symptoms.<br>Women who had breast cancer and reported PTSD symptoms had higher anxiety levels than those who did not report PTSD symptoms: 1.81 (1.23) vs. 0.67 (0.76), P<0.01.
Garcia-Torres et al., 2013 [14]<br>Spain	Convenience sample<br>22 breast cancer survivors, free of relapse, identified by staff of the local association against cancer.	ND	Srg, M: 100%<br>CT: 72.7%	8.2 (5.6), 1-21	Convenience sample<br>22 women with no history of cancer who volunteered with the same association against cancer.	ISRA (trait anxiety)	ISRA mean score (SD):<br>155.13 (71.51)	ISRA mean score (SD):<br>157.29 (82.45)	-	P=0.92	Correlation between anxiety and depression: r = 0.46, p<0.05.

Castellon et al., 2004 [15]<br><br>United States	Convenience sample<br><br>53 women who had breast cancer at or before the age of 50, with no evidence of disease or recurrence, and no history of psychiatric disorder.	0-II (100%)	CT: 34%<br>CT+HT: 34%	ND (ND), 2-5	Convenience sample<br><br>19 Healthy women recruited via fliers, newsletter articles and advertisements, or amongst the acquaintances of the hospital staff.	STAI<br>(trait anxiety)	STAI mean score (SD), by treatment<br><br>No CT:<br>31.9 (7.3)<br>CT:<br>33.1 (8.1)	STAI mean score (SD):<br><br>38.0 (9.3)	-	P=0.075	Higher STAI scores indicate more anxiety symptoms.
Weitzner et al., 1997 [16] ‡<br><br>United States	Convenience sample<br><br>60 women with age <70 years, education ≥6th grade, no history of psychiatric diagnoses,<br>>5 years disease-free, selected from those returning to the hospital for long-term follow up of cancer.	I (15%)<br>II (63%)<br>III (22%)	Srg, M: 100%	ND (ND), ≥5	Convenience sample<br><br>93 employees or volunteer workers at the same hospital with no personal or family history of breast cancer, age <70 years, education ≥6th grade, and no history of psychiatric diagnosis.	STAI<br>(mild to moderate trait anxiety)	Prevalence:<br>27%	Prevalence:<br>15%	PR=1.8 †	95%CI: 0.95-3.41	Cut-off to be identified as case defined as >1 standard deviation above the mean.
						STAI<br>(trait anxiety)	STAI mean score (SD):<br><br>35 (ND)	STAI mean score (SD):<br><br>33 (ND)	-	P<0.05 *	Adjusted for years of age and years of education.<br>Women with stage III breast cancer at diagnosis had more trait anxiety compared to the other breast cancer survivors (P<0.004).<br>Trait anxiety in breast cancer survivors was predictive of all domains of quality of life, except family functioning.
Root et al., 2015 [17]<br><br>United States	Convenience sample<br>113 women aged <70 years who had breast cancer, were post-menopausal at diagnosis, receiving HT at recruitment, with no recurrence, no neurological or psychiatric diagnoses and who did not report sleep disturbances.	I (58%)<br>II (0%)<br>III (33%)<br>IV (8%)	Srg, C: 75%<br>Srg, M: 32%<br>CT: 52%<br>RT: 78%<br>HT: 52%	4.2 (1.2)	Convenience sample<br>37 health women with no history of cancer or cancer treatment, post-menopausal, with no neurological or psychiatric diagnoses, matched for age and education (method of matching not reported).	STAI	STAI mean score (SD):<br><br>32.4 (8.6)	STAI mean score (SD):<br><br>33.1 (1.4)	-	P=0.62	Higher STAI scores indicate more anxiety symptoms.
Castellon et al., 2004 [15]<br><br>United States	Convenience sample<br><br>53 women who had breast cancer at or before the age of 50, with no evidence of disease or recurrence, and no history of psychiatric disorder.	0-II (100%)	CT: 34%<br>CT+HT: 34%	ND (ND), 2-5	Convenience sample<br><br>19 Healthy women recruited via fliers, newsletter articles and advertisements, or amongst the acquaintances of the hospital staff.	STAI<br>(state anxiety)	STAI mean score (SD), by treatment<br><br>No CT:<br>24.6 (3.6)<br>CT:<br>28.6 (8.8)	STAI mean score (SD):<br><br>33.2 (8.0)	-	P=0.01 *	Higher STAI scores indicate more anxiety symptoms.

Conroy et al., 2013 [18]<br><br>United States	Convenience sample<br><br>24 breast cancer survivors with history of non-metastatic disease and chemotherapy treated.	I (29%)<br>IIa (33%)<br>IIb (25%)<br>IIIa (8%)<br>IIIb (4%)	CT: 100%<br>RT: 79%	6.4 (2.1), 3.2-10.2	Convenience sample<br><br>23 healthy women matched for age and education (categories of matching not reported)	STAI<br>(state anxiety)	STAI mean score (SD):<br><br>30.2 (7.9)	STAI mean score (SD):<br><br>31.9 (9.1)	-	P>0.05	Higher STAI scores indicate more anxiety symptoms.
McDonald et al., 2010 [19]<br><br>ND	Convenience sample<br>29 female breast cancer patients without neurobehavioral risk factors including neurologic, medical, or psychiatric conditions, except history of depression or anxiety.	0 (14%)<br>I (35%)<br>II (48%)<br>IIIA (3%)	CT: 59%<br>RT: 69%	~1.5 (0.15)	Convenience sample<br>18 healthy controls 'demographically matched' (method of matching not reported).	STAI<br>(state anxiety)	STAI mean score (SD):<br><br>CT:<br>27.6 (8.8)<br>No CT:<br>28.3 (11.3)	STAI mean score (SD):<br><br>25.6 (7.2)	-	P>0.05	-
							Prevalence of anxiety: 7%	Prevalence of anxiety: 0%	PR= 1.25 †	95%CI: 0.12-12.65	Cut off for case: STAI-S T-score ≥65
Klein et al., 2011 [20]<br><br>France	Population based<br>652 breast cancer survivors >5 post active-treatment, randomly selected from 3 population-based cancer registries by year of diagnosis.	0-IV (ND)	Srg, C: 64.7%<br>Srg, M: 34.6%<br>CT: 45.8%<br>RT: 83.0%<br>HT: 68.0%	Diagnosed in:<br>2000: 5.6 (1.0), 5.0-5.9<br>1995: 10.3 (0.6), 10.0-10.9<br>1990: 15.6 (1.0), 15.0-15.9	Population based<br><br>1,188 women with no history of cancer randomly selected from the electoral rolls; individually matched by age (±10 years) and place of residence (area of the cancer registry, and urban/rural).	STAI<br>(state anxiety)	STAI<br>mean score (SD):<br>Diagnosed in:<br>2000: 34.4 (ND)<br>1995: 34.7 (ND)<br>1990: 33.2 (ND)	STAI<br>mean score (SD):<br><br>28.5 (ND)	-	P<0.001 *	Higher STAI scores indicate more anxiety symptoms.<br>Mean scores adjusted for age group, marital status, education, employment status, household monthly income comorbidities and hospitalization in the last 12 months.
Saleeba et al., 1996 [21] ‡<br><br>United States	52 women aged <70 years, education ≥6th grade, no history of psychiatric diagnoses, >5 years disease-free, selected from those under long-term follow up of breast cancer.	I (13%)<br>II (63%)<br>III (23%)	Srg, C: 0%<br>Srg, M: 100%	8.5 (ND), 5-18	88 women aged <70 years, with ≥6th grade of education, no history of psychiatric diagnoses and undergoing routine low risk breast cancer screening.	STAI<br>(mild to moderate state anxiety)	Prevalence: 21%	Prevalence: 7%	PR=3.0 * †	95%CI: 1.19-7.57	Cases defined as state anxiety scores above the 85th percentile for respective age group.
						STAI<br>(state anxiety)	STAI<br>mean score (SD):<br><br>33.08 (11.50)	STAI<br>mean score (SD):<br><br>31.82 (8.40)	-	P>0.05	Higher STAI scores indicate more anxiety symptoms.

ATC = Anatomic Therapeutic Chemical classification system; BSI-18 = Brief Symptom Inventory 18 [22]; CT = chemotherapy; EHR = electronic health records; HADS = Hospital Anxiety and Depression Scale [23]; HRS-A = Hamilton Rating Scale for Anxiety [24]; HSCL-25 = The Hopkins Symptom Checklist-25 [25]; HT = hormone therapy; ICD-8 = The International Classification of Diseases, Eight Revision; ICD-9-CM = The International Classification of Diseases, Ninth Revision, Clinical Modification; ICD-10 = The International

Statistical Classification of Diseases and Related Health Problems, Tenth Revision; IQR = interquartile range; ISRA = Inventory of Situations and Responses to Anxiety [26]; IT = immunotherapy; ND = not defined; OR = odds ratio; PR = prevalence ratio; RR = relative risk; RT = radiotherapy; SCL-90 = Anxiety subscale of the Symptoms Checklist-90 [27]; SD = standard deviation; SIR = standardised incidence ratio; Srg, C = Breast conserving surgery; Srg, ND = Surgery, not further specified; Srg, M = Mastectomy; Srg, R = Breast reconstructive surgery; STAI = State-Trait Anxiety Inventory [28]; yrs = years; 95%CI = 95% confidence interval.

* There was some statistical evidence (P<0.05) for a different prevalence, risk or severity of anxiety between breast cancer survivors and women who did not have cancer.

† Prevalence ratio calculated by the authors of the present study.

‡ The two studies provided results for different components of anxiety (trait and state) based on the same sample of patients.

Supplementary Table 4. Depression: main characteristics and results of the studies that evaluated the risk of depression, or the prevalence or severity of depressive symptoms, in breast cancer survivors (>1 year) and women who did not have cancer.

First author, year of publication<br><br>Country	Breast cancer survivors				Comparison group	Outcome assessment	Quantitative measure of the outcome		Relative risk estimate<br>(RR, OR, SIR, PR)	P-value or 95% confidence interval	Notes
	Type of population and main characteristics	Stage at diagnosis (%)	Breast cancer treatments (%)	Time since diagnosis/ treatment in years: mean/ median (SD), range	Type of population and main characteristics		Breast cancer survivors	Comparison group			
Electronic health records											
Suppli et al.,<br>2014 [29]<br><br>Denmark<br><br>(continues)	Population-based<br><br>All 44,494 women born in 1920-1981 and living in Denmark, who had breast cancer diagnosed in 1998-2011, without history of other cancers or major psychiatric disorder.	All	ND	5 (ND), 0-15	Population-based<br><br>1,997,669 women born in 1920-1981 and living in Denmark, without history of cancer or major psychiatric disorder	EHR, first hospital contact (in- or outpatient) for unipolar depression, as registered in the Danish Psychiatric Central Registry.<br>ICD-8 codes: 296.09, 296.29; ICD-10 codes: F32-33.9	Incidence rate: 215 per 100,000 person-years<br><br>Cumulative incidence: 1.1%	Incidence rate: 171 per 100,000 person-years<br><br>Cumulative incidence: 0.8%	All patients:<br>RR= 1.39 *	All patients:<br>95%CI: 1.27-1.52	Includes patients diagnosed with breast cancer at <1yr.
									By age:<br>30-39: RR= 0.78<br>40-49: RR= 1.56 *<br>50-59: RR= 1.35 *<br>60-69: RR= 1.41 *<br>70-79: RR= 1.25 *<br>≥80: RR= 1.56 *	By age:<br>95%CI: 0.39-1.55<br>95%CI: 1.23-1.96<br>95%CI: 1.11-1.63<br>95%CI: 1.16-1.71<br>95%CI: 1.03-1.51<br>95%CI: 1.25-1.93	RR adjusted for age (5-year intervals), calendar period (1998-2000, 2001-2004, 2005-2008, 2009-2011) and Charlson comorbidity index score (0, 1, ≥2).
									By Charlson comorbidity index:<br>0: RR= 1.47 *<br>1: RR= 1.41 *<br>≥2: RR= 1.02	By Charlson comorbidity index:<br>95%CI: 1.31-1.64<br>95%CI: 1.18-1.69<br>95%CI: 0.77-1.34	RR adjusted for age (5-year intervals), calendar period (1998-2000, 2001-2004, 2005-2008, 2009-2011).
							Cumulative incidence:	Cumulative incidence:			RR adjusted for age (5-year intervals), calendar period (1998-2000, 2001-2004, 2005-2008, 2009-2011) and Charlson comorbidity index score (0, 1, ≥2). Significant predictors of depression among breast cancer survivors: Age at diagnosis and living alone.
				0-1			0.3%	0.2%	RR= 1.70 *	95%CI: 1.41-2.05	
				1-2			0.2%	0.2%	RR= 1.48 *	95%CI: 1.19-1.83	
				2-3			0.3%	0.2%	RR= 1.64 *	95%CI: 1.31-2.06	
				3-4			0.2%	0.2%	RR= 1.20	95%CI: 0.90-1.60	
				4-5			0.2%	0.2%	RR= 1.40 *	95%CI: 1.04-1.87	
				6-8			0.2%	0.2%	RR= 1.13	95%CI: 0.90-1.41	
				9-14			0.2%	0.1%	RR= 1.09	95%CI: 0.80-1.46	
	Population-based<br>All 35,286 women born in 1920-1981 and living in Denmark, who had breast cancer diagnosed in 1998-2011 and did not use antidepressants in the 3 years before study entry, without history of other cancers or major psychiatric disorder.	All	ND	5 (ND), 0-15	Population based<br><br>1,860,552 women born in 1920-1981 and living in Denmark, without history of cancer or major psychiatric disorder and who did not use antidepressants during the three years prior to study entry.	EHR, first redeemed prescription of antidepressants (group N06A of the ATC classification system)	Incidence rate: 3,772 per 100,000 person-years<br><br>Cumulative incidence: 17.1%	Incidence rate: 1,971 per 100,000 person-years<br><br>Cumulative incidence: 9.4%	RR= 1.82 *	95%CI: 1.77-1.86	RR adjusted for age (5-year intervals), calendar period (1998-2000, 2001-2004, 2005-2008, 2009-2011) and Charlson comorbidity index score (0, 1, ≥2).<br>Predictors of depression among breast cancer survivors: age at diagnosis, living alone, not having higher education, having comorbidities, positive lymph node metastasis.

Suppli et al.,<br>2014 [29]<br><br>Denmark<br><br>(continued)	Population-based<br><br>All 35,286 women born in 1920-1981 and living in Denmark, who had breast cancer diagnosed in 1998-2011 and did not use antidepressants in the 3 years before study entry, without history of other cancers or major psychiatric disorder.	All	ND	5 (ND), 0-15	Population based<br><br>1,860,552 women born in 1920-1981 and living in Denmark, without history of cancer or major psychiatric disorder and who did not use antidepressants during the three years prior to study entry.	EHR, first redeemed prescription of antidepressants (group N06A of the ATC classification system)	Incidence rate: 3,772 per 100,000 person-years<br><br>Cumulative incidence: 17.1%	Incidence rate: 1,971 per 100,000 person-years<br><br>Cumulative incidence: 9.4%	By Charlson comorbidity index score:<br>0: RR= 2.06 *<br>1: RR= 1.49 *<br>≥2: RR= 1.25 *	By Charlson comorbidity index score:<br>95%CI: 2.00-2.12<br>95%CI: 1.40-1.58<br>95%CI: 1.15-1.36	RR adjusted for age (5-year intervals), calendar period (1998-2000, 2001-2004, 2005-2008, 2009-2011).
									By age:<br>30-39: RR= 2.07 *<br>40-49: RR= 2.12 *<br>50-59: RR= 2.12 *<br>60-69: RR= 1.89 *<br>70-79: RR= 1.59 *<br>≥80: RR= 1.29 *	By age:<br>95%CI: 1.77-2.43<br>95%CI: 1.98-2.27<br>95%CI: 2.02-2.23<br>95%CI: 1.80-1.99<br>95%CI: 1.51-1.68<br>95%CI: 1.19-1.40	Includes patients diagnosed with breast cancer at <1yr.<br><br>RR adjusted for age (5-year intervals), calendar period (1998-2000, 2001-2004, 2005-2008, 2009-2011) and Charlson comorbidity index score (0, 1, ≥2).
				5 (ND), 0-15			Cumulative incidence: 17%	Cumulative incidence: 9.4%	RR= 1.82 *	95%CI: 1.77-1.86	
				0-1			6.4%	2.1%	RR= 3.09 *	95%CI: 2.95-3.22	
				1-2			4.2%	2.1%	RR= 2.06 *	95%CI: 1.94-2.18	
				2-3			3.3%	2.1%	RR= 1.60 *	95%CI: 1.49-1.72	
				3-4			3.3%	2.1%	RR= 1.59 *	95%CI: 1.46-1.72	
				4-5			2.7%	2.1%	RR= 1.30 *	95%CI: 1.18-1.44	
				6-8			2.6%	2.1%	RR= 1.23 *	95%CI: 1.15-1.32	
				9-14			2.2%	2.0%	RR= 1.08	95%CI: 0.98-1.19	
Hjerl et al.,<br>2002 [1]<br><br>Denmark<br><br>(continues)	Population-based<br><br>All 60,431 women aged >15 years with first invasive breast cancer registered in the national Cancer Registry in 1970-1993.	All	ND	4 (ND), 0-15<br><br>(Median cohort follow up: 4 years since diagnosis; range: 0 to 15)	Population-based<br><br>Danish female population aged >15 years.	EHR, first ever psychiatric admission with affective disorders, as registered in the Danish Psychiatric Central Registry ICD-8 codes: 296.19-296.99, 298.09, 301.19, 300.49	Cumulative incidence: 0.7%	Cumulative incidence: 0.5%	SIR= 1.49 *	95%CI: 1.35-1.63	
									By calendar period:<br>1970-74: SIR=1.68 *<br>1975-79: SIR=1.60 *<br>1980-84: SIR=1.56 *<br>1985-89: SIR=1.46 *<br>1990-93: SIR=1.25	By calendar period:<br>95%CI: 1.20-2.27<br>95%CI: 1.30-1.94<br>95%CI: 1.28-1.88<br>95%CI: 1.19-1.77<br>95%CI: 0.99-1.55	Standardised incidence ratio estimated considering all follow up time since diagnosis.

Hjerl et al., 2002 [1]<br><br>Denmark<br><br>(continued)	Population-based<br><br>All 60,431 women aged >15 years with first invasive breast cancer registered in the national Cancer Registry in 1970-1993.	All	ND	4 (ND), 0-15<br><br>(Median cohort follow up: 4 years since diagnosis; range: 0 to 15)	Population-based<br><br>Danish female population aged >15 years.	EHR, first ever psychiatric admission with affective disorders, as registered in the Danish Psychiatric Central Registry ICD-8 codes: 296.19-296.99, 298.09, 301.19, 300.49	-	-	By age group:<br>15-29: SIR= 3.24<br>30-34: SIR= 0.67<br>35-39: SIR= 1.96<br>40-44: SIR= 2.92 *<br>45-49: SIR= 1.46 *<br>50-54: SIR= 2.14 *<br>55-59: SIR= 1.39 *<br>60-64: SIR= 1.46 *<br>65-69: SIR= 1.32<br>70-74: SIR= 1.22<br>75-79: SIR= 1.09<br>80-84: SIR= 1.00<br>85-89: SIR= 1.28<br>≥90: SIR= 2.43	By age group:<br>95%CI: 0.19-14.3<br>95%CI: 0.04-2.94<br>95%CI: 0.98-3.44<br>95%CI: 2.06-4.00<br>95%CI: 1.03-2.00<br>95%CI: 1.69-2.67<br>95%CI: 1.05-1.81<br>95%CI: 1.11-1.89<br>95%CI: 0.99-1.73<br>95%CI: 0.90-1.61<br>95%CI: 0.75-1.51<br>95%CI: 0.60-1.53<br>95%CI: 0.59-2.39<br>95%CI: 0.60-6.30	Standardised incidence ratio estimated considering all follow up time since diagnosis.
	Women aged >15 years with first invasive breast cancer registered in the national Cancer Registry in 1970-1993 and living in Copenhagen city area (metropolitan).	All	ND	4 (ND), 0-15	Female population aged >15 years and living in Copenhagen city area.	EHR, first ever psychiatric admission with affective disorders, as registered in the Danish Psychiatric Central Registry ICD-8 codes: 296.19-296.99, 298.09, 301.19, 300.49	-	-	SIR= 1.19	95%CI: 0.95-1.48	Standardised incidence ratio estimated considering all follow up time since diagnosis.
				1.5			-	-	SIR= 0.9	95%CI: 0.5-1.7	Approximate values estimated from the graphics provided in the original study.
				2.5			-	-	SIR= 1.2	95%CI: 0.6-2.0	
				3.5			-	-	SIR= 0.9	95%CI: 0.4-1.9	
				4.5			-	-	SIR= 1.1	95%CI: 0.4-2.3	
				5.5			-	-	SIR= 1.7	95%CI: 0.7-3.1	
				6.5			-	-	SIR= 1.3	95%CI: 0.4-2.8	
				7.5			-	-	SIR= 0.6	95%CI: 0.1-1.9	
				8.5			-	-	SIR= 0.7	95%CI: 0.1-2.2	
				9.5			-	-	SIR= 0.4	95%CI: 0.0-1.9	
				10.5			-	-	SIR= 2.5	95%CI: 0.9-5.4	
				13.0			-	-	SIR= 0.2 *	95%CI: 0.0-0.9	
	Women aged >15 years with first invasive breast cancer registered in the national Cancer Registry in 1970-1993 and living outside Copenhagen city area.	All	ND	4 (ND), 0-15	Female population aged >15 years and living outside Copenhagen city area.	EHR, first ever psychiatric admission with affective disorders, as registered in the Danish Psychiatric Central Registry ICD-8 codes: 296.19-296.99, 298.09, 301.19, 300.49	-	-	SIR= 1.57 *	95%CI: 1.41-1.75	Standardised incidence ratio estimated considering all follow up time since diagnosis.<br><br>Approximate values estimated from the graphics provided in the original study.
				1.5			-	-	SIR= 2.1 *	95%CI: 1.6-2.6	
				2.5			-	-	SIR= 1.3	95%CI: 0.9-1.8	
				3.5			-	-	SIR= 1.5 *	95%CI: 1.1-2.1	
				4.5			-	-	SIR= 1.4	95%CI: 0.9-2.0	
				5.5			-	-	SIR= 1.6 *	95%CI: 1.1-2.4	
				6.5			-	-	SIR= 1.4	95%CI: 0.8-1.9	
				7.5			-	-	SIR= 1.1	95%CI: 0.6-1.8	
				8.5			-	-	SIR= 1.0	95%CI: 0.5-1.8	
				9.5			-	-	SIR= 0.8	95%CI: 0.3-1.6	
				10.5			-	-	SIR= 0.9	95%CI: 0.4-1.8	
				11.5			-	-	SIR= 0.9	95%CI: 0.3-1.9	
				12.5			-	-	SIR= 1.1	95%CI: 0.4-2.3	
				13.5			-	-	SIR= 1.6	95%CI: 0.6-3.1	

Hung et al., 2013 [2]<br><br>Taiwan	Population-based<br><br>26,629 women with no prior mood disorder and cancer, with breast cancer registered in the National Health Insurance Database in 2000-2005.	All	ND	2.7 (ND), ND-7<br><br>(median follow up years for breast cancer survivors: 2.7; for matched cohort: 3.21)	Population-based<br><br>26,629 women randomly selected from 1 million women who did not have breast cancer registered in the National Health Insurance Database; matched for age and Charlson comorbidity score (matching, categories not reported).	EHR, recorded in the Registry for Catastrophic Illness with an ICD-9-CM code for major depressive disorder (296.2X-296.3X, 300.4, 311.X)	Incidence rate: 14.55 per 1,000 person-years<br><br>Cumulative incidence= 4.4%	Incidence rate: 7.51 per 1,000 person-years<br><br>Cumulative incidence= 2.6%	RR=1.94 *	95%CI: 1.76-2.13	Includes patients diagnosed with breast cancer at <1yr.
				2			4%	2%	RR=2.0 * †	95%CI: 1.80-2.22	Approximate cumulative incidence values estimated from the graphics provided in the original study.<br><br>P value for the log-rank test comparing the Kaplan-Meier curves: P<0.001
				4			5%	3%	RR=1.7 * †	95%CI: 1.53-1.82	
				6			6%	4%	RR=1.5 * †	95%CI: 1.39-1.62	
Earle et al., 2007 [30]<br><br>United States	Convenience sample<br><br>463 women who had non-metastatic cancer registered with a private health care insurance company and not receiving active treatment; patients had no evidence of recurrence.	Non-metastatic	ND	ND (ND), ≥5	Convenience sample<br><br>3,108 women without cancer registered with a private health care insure company; matched for age and clinic location (individual matching, categories not reported).	EHR, ICD-9 codes for diagnoses of psychotic depression and dysthymia in an administrative database from a health care plan.	Prevalence: 22.5%	Prevalence: 18.1%	PR=1.24 * †	95%CI: 1.03-1.50<br>P=0.04	Breast cancer survivors had more visits with mental health providers compared to women without cancer.
Kim et al., 2017 [31]<br><br>Korea	Population based<br><br>2,130 women who had mastectomy for breast cancer, randomly selected from the National Health Insurance Database	All	Srg, M: 100%	0	Population based<br><br>8,520 women never diagnosed with cancer randomly selected from the same database as the cases matched for age, income, region, pre-operative depression (individual matching, categories not reported).	EHR, ICD-10 codes for depression	Prevalence:<br>5.5%	Prevalence:<br>2.5%	PR= 2.20 * †	95%CI: 1.76-2.74	-
				1			4.8%	3.1%	PR= 1.55 * †	95%CI: 1.24-1.94	
				2			4.4%	3.0%	PR= 1.47 * †	95%CI: 1.14-1.89	
				3			4.4%	3.1%	PR= 1.42 * †	95%CI: 1.08-1.87	
				4			4.1%	4.0%	PR= 1.03 †	95%CI: 0.76-1.39	
				5			4.4%	3.5%	PR= 1.26 †	95%CI: 0.91-1.75	
				6			4.5%	4.3%	PR= 1.05 †	95%CI: 0.73-1.49	
				7			5.0%	3.9%	PR= 1.28 †	95%CI: 0.86-1.91	
				8			6.0%	3.9%	PR= 1.54 †	95%CI: 0.99-2.39	
				9			5.4%	4.7%	PR= 1.15 †	95%CI: 0.67-1.98	
				10			8.1%	4.5%	PR= 1.80 †	95%CI: 0.93-3.47	

Yang et al., 2017 [4]<br><br>Sweden	Population based<br><br>All 4,402 women diagnosed with an in situ breast cancer at the age of 20-80 years between 2001-2009	0	ND	4.7 (4.4), 0-10<br><br>(median (IQR) duration of follow up: 4.7 (4.4))	Population based<br><br>452,507 women randomly selected from the respondents to the 1990 census	EHR, ICD-10 diagnostic codes for depression (F32-F33) at in patient or outpatient hospital visits	Cumulative incidence: 1.3%	Cumulative incidence: 1.2%	SIR= 1.03	95%CI: 0.80-1.34	Standardised incidence ratios were standardised by calendar period (1-year categories), age (5-year categories), and region of residence (North, Stockholm- Gotland, South, Southeast, Uppsala-Orebro, West).
									By age group:<br>20-44: SIR= 1.48<br>45-54: SIR= 0.84<br>55-64: SIR= 1.01<br>65-80: SIR= 1.07	By age group:<br>95%CI: 0.84-2.61<br>95%CI: 0.51-1.36<br>95%CI: 0.61-1.68<br>95%CI: 0.62-1.85	
				0-0.5			0.1%	0.1%	SIR= 0.77	95%CI: 0.29-2.05	
				0.5-1			0.1%	0.1%	SIR= 1.14	95%CI: 0.51-2.54	
				1-2			0.2%	0.2%	SIR= 0.91	95%CI: 0.47-1.74	
				2-5			0.6%	0.5%	SIR= 1.15	95%CI: 0.78-1.70	
				5-10			0.3%	0.3%	SIR= 1.00	95%CI: 0.57-1.76	
				4.7 (4.4), 0-10<br><br>(median (IQR) duration of follow up: 4.7 (4.4))		EHR, being prescribed an antidepressant (group N06A of the ATC classification system)	Cumulative incidence: 3.6%	Cumulative incidence: 2.3%	SIR= 1.58 *	95%CI: 1.36-1.85	Standardised incidence ratios were standardised by calendar period (1-year categories), age (5-year categories), and region of residence (North, Stockholm- Gotland, South, Southeast, Uppsala-Orebro, West).
									By age group:<br>20-44: SIR= 1.36<br>45-54: SIR= 1.93 *<br>55-64: SIR= 1.54 *<br>65-80: SIR= 1.40 *	By age group:<br>95%CI: 0.84-2.23<br>95%CI: 1.48-2.53<br>95%CI: 1.15-2.07<br>95%CI: 1.05-1.87	
				0-0.5			-	-	SIR= 2.09	95%CI: 1.57-2.79	
				0.5-1			-	-	SIR= 1.49	95%CI: 1.04-2.13	
				1-2			-	-	SIR= 1.70	95%CI: 1.30-2.22	
				2-4.5			-	-	SIR= 1.12	95%CI: 0.79-1.59	
	Population based<br><br>All 40,849 women diagnosed with an invasive breast cancer at the age of 20-80 years between 2001-2009	I-IV	ND	4.5 (4.5), 0-10<br><br>(median (IQR) duration of follow up: 4.4 (4.5))	Population based<br><br>452,507 women randomly selected from the respondents to the 1990 census	EHR, ICD-10 diagnostic codes for depression (F32-F33) at in patient or outpatient hospital visits	Cumulative incidence: 1.9%	Cumulative incidence: 1.2%	SIR= 1.57 *	95%CI: 1.46-1.69	SIR standardised by calendar period, age, and region.<br><br>Predictors of depression among breast cancer survivors: having comorbidities and positive lymph nodes.
									By age group:<br>20-44: SIR= 1.69<br>45-54: SIR= 1.70<br>55-64: SIR= 1.56<br>65-80: SIR= 1.38	By age group:<br>95%CI: 1.42-2.01<br>95%CI: 1.50-1.93<br>95%CI: 1.36-1.79<br>95%CI: 1.19-1.59	
				0-0.5			0.2%	0.1%	SIR= 1.83 *	95%CI: 1.48-2.26	
				0.5-1			0.3%	0.1%	SIR= 2.48 *	95%CI: 2.07-2.97	
				1-2			0.4%	0.2%	SIR= 2.04 *	95%CI: 1.76-2.36	
				2-5			0.6%	0.5%	SIR= 1.29 *	95%CI: 1.14-1.46	
				5-10			0.3%	0.3%	SIR= 1.18	95%CI: 0.99-1.41	
				4.5 (4.5), 0-10<br><br>(median (IQR) duration of follow up: 4.4 (4.5))		EHR, being prescribed an antidepressant (group N06A of the ATC classification system)	Cumulative incidence: 9.2%	Cumulative incidence: 2.2%	SIR= 1.95 *	95%CI: 1.86-2.04	SIR standardised by calendar period, age, and region.
									By age group:<br>20-44: SIR= 2.43 *<br>45-54: SIR= 2.23 *<br>55-64: SIR= 2.00 *<br>65-80: SIR= 1.64 *	By age group:<br>95%CI: 2.14-2.76<br>95%CI: 2.02-2.45<br>95%CI: 1.83-2.18<br>95%CI: 1.51-1.77	
				0-0.5			-	-	SIR= 2.14 *	95%CI: 1.95-2.36	
				0.5-1			-	-	SIR= 2.62 *	95%CI: 2.40-2.87	
				1-2			-	-	SIR= 1.92 *	95%CI: 1.76-2.09	
				2-4.5			-	-	SIR= 1.34 *	95%CI: 1.20-1.49	

Khan et al., 2010 [3]<br><br>United Kingdom	Population-based<br><br>16,938 women aged ≥30 with breast cancer registered in the UK General Practice Research Database.	All	ND	ND (ND), ≥5	Population-based<br><br>67,649 women who did not have breast or colorectal cancer at beginning of follow; individual matching for age (± 1 year) and primary care practice (small area).	EHR, primary care consultations for depression recorded with Read codes	Prevalence: 9.6%	Prevalence: 8.9%	OR= 1.06	95%CI: 1.00-1.14	Odds ratio adjusted for Charlson comorbidity score, previous history of depression and death.
						EHR, ≥1 prescription of antidepressants	Prevalence: 23.7%	Prevalence: 20.2%	OR= 1.16 *	95%CI: 1.11-1.22	Odds ratio adjusted for Charlson comorbidity score, number of consultations, and death.
**Cohort studies involving scales**											
Aerts et al., 2014 [32]<br><br>ND	Convenience sample<br><br>66 women who had breast-conserving surgery for early breast cancer and no recurrence during follow up.	'Early-stage' (100%)	Srg, C: 100%<br>CT: 24.7%<br>RT: 76.5%<br>HT: 70.3%	~ 1<br><br>(follow up at 1 year)	Convenience sample<br><br>149 women with no history of cancer recruited in: a gynaecology outpatient clinic, an organisation for elderly women and online; matched for age (method not reported).	BDI	BDI mean score (SD)<br><br>7.71 (8.00)	BDI mean score (SD)<br><br>5.28 (5.34)	-	P=0.02 *	Higher CES-D scores indicate more depressive symptoms.<br><br>Women who had advanced stage or had had relapse were excluded at baseline, as were those who had recurrence or a second cancer during follow up.
	Convenience sample<br><br>48 women who had mastectomy for early breast cancer at one university hospital and no recurrence during follow up.	'Early-stage' (100%)	Srg, M: 100%<br>CT: 44.1%<br>RT: 45.6%<br>HT: 54.4%	~ 1<br><br>(follow up at 1 year)			BDI mean score (SD)<br><br>8.85 (6.79)	BDI mean score (SD)<br><br>5.28 (5.34)	-	P<0.01 *	
Ancoli-Israel et al., 2014 [33]<br><br>United States	Convenience sample<br><br>44 women who had been diagnosed with breast cancer 1 year before, and scheduled to receive ≥4 cycles of CT, with no psychological impairments and not receiving RT at recruitment.	I (27.9%)<br>II (39.7%)<br>III (30.9%)<br>Unknown (1.5%)	Srg, C: 45.6%<br>Srg, M: 49.7%<br>CT: 100%	~ 1<br><br>(follow up at 1 year after CT)	Convenience sample<br><br>35 cancer-free friends of the women who had breast cancer with no psychological impairments at the time of recruitment; individual matching for age (±5 years), ethnicity and education (categories of ethnicity and education not reported).	CES-D	CES-D mean score (SD)<br><br>10.0 (ND)	CES-D mean score (SD)<br><br>4.8 (ND)	-	P=0.04 *	Higher CES-D scores indicate more depressive symptoms.<br><br>Mean scores adjusted for age and body mass index.

Kesler et al., 2013 [34]<br><br>United States	Convenience sample<br><br>44 women who had breast cancer recruited via support groups and advertisements; patients excluded if they had had disease recurrence or relapse.	I-IIIA	Srg, ND: 100%<br>CT: 100%	4.8 (3.4),<br>1-12	Convenience sample<br><br>38 healthy female controls recruited through advertisements	CAD	CAD mean score (SD):<br><br>48.8 (8.2)	CAD mean score (SD):<br><br>48.0 (7.2)	-	P=0.08	-
Bailey et al., 2010 [35]<br><br>United States	Convenience sample<br><br>515 patients with first primary breast cancer, aged >40 years, with no cognitive impairment or prior history of breast cancer, post active treatment and who spoke English.	0 (34.4%)<br>I (51.4%)<br>IIA (14.2%)	Srg, ND: 100%	~ 1<br><br>(follow up at 12 months after surgery)	Convenience sample<br><br>496 women who had a normal/benign mammogram, aged >40 years, with no cognitive impairment or prior history of breast cancer, and who spoke English; frequency matched for age (40-50, 50-69, ≥70 years).	CES-D<br><br>Cut-off score for case: ≥16	Prevalence:<br>47.4%	Prevalence:<br>52.6%	PR= 0.9 †	95%CI: 0.80-1.02	Women with more advanced disease at diagnosis (stage IIA) had significantly more depression compared to those diagnosed at earlier stages.
Hermelink et al., 2017 [36]<br><br>Germany	Convenience sample<br><br>56 women aged 18-65 years, newly diagnosed with breast cancer, with no previous history of neurological or psychotic disorders and no previous systemic treatment for cancer	0 (7%)<br>I (42%)<br>II (41.4%)<br>III (%9.6)	CT: 100%<br>HT: 73.9%<br>vs.<br>CT: 0%<br>HT: 80.7%	~ 1<br><br>(follow up at 1 year after diagnosis)	Convenience sample<br><br>150 women aged 18-65 years, who never had cancer, and attended the same institution as cases for breast imagining and did not require further tests.	PHQ-D	PHQ-D mean score<br><br>CT: 4.7 (4.5)<br>No CT: 4.2 (4.5)	PHQ-D mean score<br><br>2.7 (3.0)	-	P=0.03 *<br><br>(for differences between the three groups)	Higher PHQ-D mean scores indicate more depressive symptoms.
Lee et al., 2011 [37]<br><br>Korea	Convenience sample<br><br>206 patients aged ≥18 years who had been diagnosed with breast cancer 1 year before	I-IIA (71.2%)<br>IIB-III (25.0%)	Srg, C: 82.5%<br>Srg, M: 16.4%<br>CT: 86.7%<br>RT: 82.5%<br>HT: 82.2%	~ 1<br><br>(follow up at 1 year after diagnosis)	Population-based<br><br>Nationally representative sample of 496 adult women.	SDS	SDS mean score<br><br>38.1 (0.94)	SDS mean score<br><br>38.8 (0.37)		P=0.514	Mean scores adjusted for age, menopausal status, comorbidity, marital status, educational level, religious practice, job status, monthly income, body mass index, smoking status, drinking status, regular exercise, propensity score, and subscales of social support.
							Prevalence:<br>49.3%	Prevalence:<br>46.6%	PR=1.06 †	95%CI: 0.89-1.25<br>P=0.516	Cut-off score for case: ≥50

Cross-sectional studies involving scales											
Bizetti Pelai et al., 2012 [38]	Convenience sample<br><br>89 women who had surgery for breast cancer at <10 years	ND (ND)	Srg, BCS: 37%<br>Srg, M: 50-63%<br>RT: 2-11%<br>CT: 24-30%<br>CT+RT: 54-60%	3.7 (ND), ≤10	Convenience sample<br><br>43 women without breast cancer, or neurological or orthopaedic impairments of the upper limbs	BDI	Prevalence: 41.6%	Prevalence: 28.0%	PR=1.49 †	95%CI: 0.97-2.28	Cut-off score for case: ≥10
Castellon et al., 2004 [15]<br><br>United States	Convenience sample<br><br>53 women who had breast cancer at or before the age of 50, with no evidence of disease or recurrence, and no history of psychiatric disorder.	0-II (100%)	CT: 34%<br>CT+HT: 34%	ND (ND), 2-5	Convenience sample<br><br>19 Healthy women recruited via fliers, newsletter articles and advertisements, or amongst the acquaintances of the hospital staff.	BDI	BDI mean score (SD):<br><br>No CT:<br>7.0 (4.5)<br><br>CT:<br>6.3 (5.1)	BDI mean score (SD):<br><br>7.8 (7.9)	-	P=0.63	-
Weitzner et al., 1997 [16]<br><br>United States	Convenience sample<br><br>60 women with age <70 years, education ≥6th grade, no history of psychiatric diagnoses, >5 years disease-free, selected from those returning to the hospital for long-term follow up of cancer.	I (15%)<br>II (63%)<br>III (22%)	Srg, M: 100%	ND (ND), ≥5	Convenience sample<br><br>93 employees or volunteer workers at the same hospital with no personal or family history of breast cancer, age <70 years, education ≥6th grade, and no psychiatric history.	BDI<br><br>Scale applied as part of a psychiatric interview	BDI mean score (SD):<br><br>7 (ND)	BDI mean score (SD):<br><br>5 (ND)	-	P<0.003 *	Adjusted for years of age and years of education.<br><br>Among breast cancer survivors, lower BDI scores, indicating less depression, were associated with better quality of life for all domains (P<0.02), except in the family one.
							Prevalence: 29%	Prevalence: 15%	PR= 1.93 * †	95%CI: 1.03-3.61	Cut-off score for case: >12 (mild to moderate depression)
Garcia-Torres et al., 2013 [14]<br><br>Spain	Convenience sample<br><br>22 breast cancer survivors free of relapse identified by staff of the local association against cancer.	ND	Srg, M: 100%<br>CT: 72.7%	8.2 (5.6), 1-21	Convenience sample<br><br>22 women with no history of cancer who volunteered with the same association against cancer.	BDI-II	BDI-II mean score (SD):<br>13.13 (7.83)	BDI-II mean score (SD):<br>8.18 (7.78)	-	P=0.02 *	Correlation between anxiety and depression: r = 0.46, p<0.05;<br><br>Cut-off score for case: >14 (slight to severe depression)
							Cognitive-affective component:<br>5.86 (4.06)	Cognitive-affective component:<br>3.72 (3.88)	-	P=0.03 *	
							Motivational-somatic component:<br>6.81 (5.07)	Motivational-somatic component:<br>3.81 (2.92)		P=0.02 *	
							Prevalence: 40%	Prevalence: 18%	PR= 2.22 †	95%CI: 0.79-6.21	

Nguyen et al., 2013 [39]<br><br>United States	Convenience sample<br><br>57 women survivors of breast cancer, aged over 65 years, without recurrence, recruited from a cancer registry	I-IIIA (100%)	RT: 53%<br>CT: 47%	>10	Convenience sample<br><br>30 healthy female adults, selected in the community for a previous study.	BDI-II	BDI-II mean score (SD):<br>4.86 (4.07)	BDI-II mean score (SD):<br>4.03 (3.38)	-	P=0.39	-
Cohen et al., 2011 [5]<br><br>Israel	Convenience sample<br><br>56 married Israeli Arab breast cancer survivors, post treatment and free of disease recruited from one hospital.	I-III (ND%)	Srg, C: 48.2%<br>Srg, M: 51.8%<br>Srg, R: 12.5%<br>CT: 85.7%<br>RT: 85.7%<br>HT: 58.9%	4.8 (4.2), 1-17	Convenience sample<br><br>66 married and healthy Arab women living in Israel, approached in community settings; individual matching for age and education (matching categories not reported).	BSI-18	BSI-18 mean score (SD):<br>2.0 (1.1)	BSI-18 mean score (SD):<br>1.8 (0.8)	-	P>0.05	Higher levels of depression associated with higher levels of anxiety and somatization, and lower levels of support in both groups (P<0.05).<br><br>Higher levels of depression associated with lower body image in breast cancer survivors (P=0.05).
Broeckel et al., 2002 [40]<br><br>United States	Convenience sample<br><br>58 breast cancer survivors who had a spouse or partner, free of recurrence for >5 years, with no known neurological disorder, and no history of other cancer.	I (26%)<br>II (62%)<br>III (10%)<br>Unknown (2%)	Srg, C: 50%<br>Srg, M: 47%<br>CT: 100%<br>RT: 71%<br>HT: 48%	7.7 (2.3), 5.2-15.2	Convenience sample<br><br>61 women with no history of cancer who had a spouse or partner, recruited among the friends of the women who had breast cancer; individual matching for age (± 6 years).	CES-D	CES-D mean score (SD):<br>8.01 (6.34)	CES-D mean score (SD):<br>4.75 (4.12)	-	P≤0.05 *	Higher CES-D score indicates more depressive symptoms.<br><br>Correlation between depression scores and problems in sexual function: r = 0.27, P≤0.05
Claus et al., 2006 [41]<br><br>United States	Population-based<br><br>All 795 women diagnosed with DCIS in 1994-1998, with no history of invasive breast cancer; reinterviewed on average 6.2 years after first interview.	0 (100%)	Srg, C: 35.5%<br>Srg, M: 14.0%	5.8 (1.0), ND	Population based<br><br>702 women selected by random-digit-dialling methods, with no history of DCIS or invasive breast cancer; frequency matched for age (± 5 years) and geography. Reinterviewed on average at 6.0 (0.6) years after first interview.	CES-D	CES-D mean score (95%CI):<br>8.3 (7.7-8.9)	CES-D mean score (95%CI):<br>7.2 (6.6-7.8)	-	P<0.05 *	Higher CES-D score indicates more depressive symptoms.<br><br>Mean scores adjusted for age at diagnosis/interview, race (white/non-white), education (college degree/no college) menopausal status, comorbid conditions (myocardial infarction, stroke, cancer), marital status (married/living as married vs. not), time since diagnosis and case/control status.
			Srg, C: 100%	5.7 (1.1)			CES-D mean score (SD):<br>8.1 (7.2-9.0)	CES-D mean score (SD):<br>7.2 (6.6-7.8)	-	P>0.05	
			Srg, C: 100%<br>RT: 100%	5.7 (1.1)			CES-D mean score (SD):<br>8.7 (7.9-9.5)	CES-D mean score (SD):<br>7.2 (6.6-7.8)	-	P<0.05 *	
			Srg, M: 100%	6.0 (0.9)			CES-D mean score (SD):<br>7.4 (5.8-8.9)	CES-D mean score (SD):<br>7.2 (6.6-7.8)	-	P>0.05	

Conroy et al., 2013 [18]	Convenience sample<br><br>24 breast cancer survivors with history of non-metastatic disease and CT treated.	I (29%)<br>IIa (33%)<br>IIb (25%)<br>IIIa (8%)<br>IIIb (4%)	CT: 100%<br>RT: 79%	6.4 (2.1), 3.2-10.2	Convenience sample<br><br>23 healthy women matched for age and education (matching method not reported).	CES-D	CES-D mean score (SD):<br><br>7.5 (5.8)	CES-D mean score (SD):<br><br>8.7 (6.9)	-	P>0.05	Higher CES-D score indicates more depressive symptoms.
Koppelmans et al., 2012 [42]<br><br>The Netherlands	Convenience sample<br><br>196 women who had been treated for breast cancer between 1976 and 1995, were aged between 50 and 80 years in 2008, did not have recurrence or a second primary cancer and never used adjuvant hormone therapy.	I-III (100%)	HT: 0%<br>CT: 100%	21 (4.4), ND	Convenience sample<br><br>All 1,509 women without a history of cancer who were between 50 and 80 years of age at the time of the assessments, selected from a larger population-based cohort.	CES-D	CES-D mean score (SD):<br><br>4.7 (8.0)	CES-D mean score (SD):<br><br>6.7 (8.4)	-	P<0.05 *	Mean score adjusted for age (format of the variable not reported).<br><br>Higher CES-D score indicates more depressive symptoms.
McDonald et al., 2010 [19]<br><br>ND	Convenience sample<br><br>29 female breast cancer patients without neurobehavioral risk factors including neurologic, medical, or psychiatric conditions, except history of depression or anxiety	0 (14%)<br>I (35%)<br>II (48%)<br>IIIA (3%)	CT: 59%<br>RT: 69%	~1.5 (0.15)	Convenience sample<br><br>18 healthy controls 'demographically matched' (matching method not reported).	CES-D	CES-D mean score (SD):<br><br>CT:<br>6.8 (6.2)<br><br>No CT:<br>7.5 (10.4)	CES-D mean score (SD):<br><br>4.7 (8.9)	-	P>0.05	Higher CES-D score indicates more depressive symptoms.
							Prevalence of depression:<br>13.8%	Prevalence of depression:<br>5.6%	PR= 2.46 †	95%CI: 0.30 - 20.20	Cut-off for case: CES-D score ≥16
Otte et al., 2010 [43]<br><br>United States	Convenience sample<br><br>246 breast cancer survivors free of cancer at recruitment, with no history of other cancers and able to speak, read and write English	I (ND)<br>II (ND)<br>III (ND)	Srg, C: 42%<br>Srg, M: 59%<br>CT: 89%<br>RT: ND<br>HT: 33%	5.6 (2.0), 2-10	Convenience sample<br><br>246 women in general good health with no history of breast cancer recruited by acquaintance referral, self-referral or from corporative group; individual matching for age (±5 years).	CES-D	CES-D mean score (SD):<br><br>11.53 (9.60)	CES-D mean score (SD):<br><br>9.00 (9.20)	-	P<0.01 *	Higher CES-D score indicates more depressive symptoms.<br><br>Depressive scores were correlated with sleep-wake disturbances (p<0.05).

Root et al., 2015 [17]	Convenience sample<br><br>113 women aged <70 years who had breast cancer, were post-menopausal at diagnosis, receiving HT at recruitment, with no recurrence, no neurological or psychiatric diagnoses and who did not report sleep disturbances.	I (58%)<br>II (0%)<br>III (33%)<br>IV (8%)	Srg, C: 75%<br>Srg, M: 32%<br>CT: 52%<br>RT: 78%<br>HT: 52%	4.2 (1.2)	Convenience sample<br><br>37 health women with no history of cancer or cancer treatment, post-menopausal, with no neurological or psychiatric diagnoses, matched for age and education (matching method not reported).	CES-D	CES-D mean score (SD):<br><br>8.6 (8.2)	CES-D mean score (SD):<br><br>7.8 (6.5)	-	P=0.59	Higher CES-D score indicates more depressive symptoms.
Von Ah et al., 2009 [44]<br><br>United States	Convenience sample<br><br>52 women aged ≥40 years, who had breast cancer and had completed primary treatment ≥1 year ago, no cancer relapse, no metastatic disease or other cancer, and no history of psychiatric illnesses, recruited from cancer support groups, advertisements in churches and community centres, or by referral of enrolled participants.	I-II (50%)<br>III (ND)	Srg, C: 66%<br>Srg, M: 33%<br>CT: 55.8%<br>RT: 80.8%<br>HT: 79%	4.6 (2.8), 1.2-15.8	Convenience sample<br><br>52 women aged ≥40 years, with no history of cancer, no history of psychiatric illnesses, recruited from cancer support groups, advertisements in churches and community centres, or by referral of enrolled participants; individual matching for age (±5 years) and education (±3 years).	CES-D	CES-D mean score (SD):<br><br>10.8 (8.1)	CES-D mean score (SD):<br><br>9.5 (8.2)	-	P=0.415	Higher CES-D score indicates more depressive symptoms.
Von Ah et al., 2012 [45]<br><br>United States	Convenience sample<br><br>62 non-Hispanic African American women diagnosed with non-metastatic breast cancer and able to read and write English, recruited by medical record review and by self-referral.	I-IIB (85.7%)<br>IIIB (14.3%)	Srg, C: 0%<br>Srg, M: 60.3%<br>CT & RT: 54.6%<br>HT: ND	5.0 (2.7), 2-10	Convenience sample<br><br>78 African American women with no history of breast cancer, recruited through community advertisements and events.	CES-D	CES-D mean score (SD):<br><br>12.2 (11.7)	CES-D mean score (SD):<br><br>11.6 (11.0)	-	P=0.757	Higher CES-D score indicates more depressive symptoms.<br><br>Mean scores adjusted for age, income, years of education and body mass index.

Frazzetto et al., 2012 [46]<br><br>Italy	Convenience sample<br><br>32 women aged 66-75 years, with breast cancer recurrence ≥10 years after initial diagnosis, recruited in one hospital.	ND	ND	ND (ND), ≥10	Convenience sample<br><br>35 women in 'good health' previously recruited in a hospital for a study on health-related quality of life	GDS	Prevalence: 33.3%	Prevalence: 20.0%	PR= 1.67 †	95%CI: 0.73-3.80	Cut-off score for case: 10-19<br>(mild depression)
							Prevalence: 50.0%	Prevalence: 8.6%	PR= 5.81 * †	95%CI: 1.87-18.08	Cut-off score for case: 20-30<br>(severe depression)
							Prevalence: 83.3%	Prevalence: 28.6%	PR= 2.91 * †	95%CI: 1.69-5.03	Cut-off score for case: ≥10<br>(mild to severe depression)
Calvio et al., 2010 [7]<br><br>United States	Convenience sample<br><br>122 breast cancer survivors ≥1 year post treatment, working full-time for ≥1 year, with computer and internet, recruited via advertisements and flyers.	I (36.9%)<br>II (44.3%)<br>III (17.2%)	Srg, ND: 96.7%<br>CT: 82.8%<br>RT: 73.0%<br>HT: 45.9%<br>IT: 13.1%	3.1 (2.4), 1-10	Convenience sample<br><br>113 women without a previous cancer diagnosis, working full-time for ≥1 year, with computer and Internet, recruited via advertisements and flyers.	HADS	HADS mean score (SD):<br><br>4.6 (3.3)	HADS mean score (SD):<br><br>3.2 (2.7)	-	P<0.001 *	Higher scores indicate more depressive symptoms.<br><br>Mean scores adjusted for marital status (cohabitating with partner vs. single/not cohabitating), race (Caucasian vs. non-Caucasian), ethnicity (Hispanic vs. non-Hispanic), age (<40, 41-50, 51-65), income (0-39,000; 40-59,000; 60-79,000; 80-89,000; 80-99,000; ≥100,000), and menopausal status (currently going through, premenopausal, postmenopausal).
Boehmer et al., 2015 [6]<br><br>ND	Convenience sample<br><br>85 lesbian or bisexual breast cancer survivors post-active treatment recruited via advertisements, flyers, etc. (3.5% of whom had had cancer recurrence)	I-III (100%)	ND	4.5 (ND), 1-10	Convenience sample<br><br>85 lesbian or bisexual women with no history of cancer, no prophylactic mastectomy or oophorectomy, and not using hormone therapy, recruited via flyers, advertisements, etc.; individual matching for age (± 3 years) and partner status (partnered vs. unpartnered).	Antidepressants intake (self-reported)	Prevalence: 34.1%	Prevalence: 21.2%	PR=1.61 †	95%CI: 0.97-2.67	Depression was more common in women taking any psychopharmacological medication, compared to those who did not (OR=2.29, 95%CI: 1.02 to 5.15), and less common in women with higher levels of physical activity (OR= 0.31, 95%CI: 0.11-0.84).
						HADS score ≥8	Prevalence: 15.3%	Prevalence: 12.9%	PR=1.19 †	95%CI: 0.56-2.50	

Dahl et al., 2011 [8]<br><br>Norway	Convenience sample<br><br>337 tumor free breast cancer survivors treated with radiotherapy during 1998 and 2002 in one hospital.	II (ND)<br>III (ND)	Srg, C: 24%<br>Srg, M: 76%<br>CT: 82%<br>RT: 100%<br>HT: 81%	3.9 (ND), 2.6-6.9	Convenience sample<br><br>1,685 women randomly selected from a population-based sample with no history of cancer and had complete data for questionnaires; individual matching for age (± 5 years).	HADS	HADS mean score (SD):<br>3.1 (3.3)	HADS mean score (SD):<br>3.7 (3.1)	-	P<0.001 *	Mean scores adjusted for level of education, on disability pension and menopausal status.<br>Higher scores of HADS for depression were associated in univariate analysis with more insomnia symptoms in breast cancer survivors and in controls (P<0.05).
Miao et al., 2016 [9]	Convenience sample<br><br>23 patients with breast cancer who had been treated with chemotherapy at a local hospital	I-III (100%)	CT: 100%	3 (0.3),	Convenience sample<br><br>26 age matched healthy controls selected amongst patients relatives and local universities (matching method not reported).	HRS-D	Mean score (SD)<br><br>5.04 (1.19)	Mean score (SD)<br><br>4.88 (1.23)	-	P=0.650	Higher score indicates more anxiety symptoms
Rubino et al., 2007 [10]<br><br>Italy	Convenience sample<br><br>33 consecutive patients who had had breast-reconstruction after mastectomy, in 2001-2002.	ND	Srg, M: 100%<br>Srg, R: 100%	ND (ND), >1	Convenience sample<br><br>33 women, randomly selected amongst university staff.	HRS-D ‡<br><br>Score ≥8	Prevalence:<br>45.4%	Prevalence:<br>12.1%	PR=3.76 * †	95%CI: 1.39-10.14	A P-value of 0.02 was reported in the article, for the chi-square test of differences in depression between groups.
Boele et al., 2015 [11]<br><br>The Netherlands	Convenience sample<br><br>Post-menopausal breast cancer survivors with no psychiatric history, who did not receive CT, selected from medical records. 20 exposed to HT, 43 in the Srg+RT group.	ND	Srg, ND: 95%<br>CT: 0%<br>RT: 65%<br>HT: 100% / 0%	Exposure to HT: 3.2 (1.9), 1.5-7;<br><br>Unexposed to HT: 2.8 (0.3), 2.3-3.3.	Convenience sample<br><br>44 friends or family members of the women who had had breast cancer, with no history of breast cancer; matched for age and education (method of matching not reported).	HSCL-25	HSCL-25 mean score (SD):<br><br>HT:<br>12.89 (8.40)<br><br>No HT: 15.46 (15.82)	HSCL-25 mean score (SD):<br><br>11.92 (10.97)	-	P=0.43	Higher HSCL-25 score indicates more depressive symptoms.<br><br>P-value adjusted for age and premorbid IQ.

Kreukels et al., 2008 [12]<br><br>The Netherlands	Convenience sample<br><br>63 women who had non-metastatic breast cancer, with no history of psychiatric diseases.	I-III (100%)	CT: 100%<br>HT: 40%	~ 1	Convenience sample<br><br>60 friends or family of the patients with the same age who never had cancer; matched for age (matching method not reported).	HSCL-25	HSCL-25 mean score (SD):<br><br>17.1 (13.6)	HSCL-25 mean score (SD):<br><br>9.6 (9.2)	-	P<0.001 *	Higher HSCL-25 score indicates more depressive symptoms.
Min et al., 2010 [47]<br><br>Korea	Convenience sample<br><br>52 women who had breast cancer treated with mastectomy and followed up immediate reconstruction with latissimus dorsi myocutaneous flap, recruited in one cancer center (3% had disease recurrence).	0 (15.4%)<br>I (40.4%)<br>II (30.7%)<br>III (13.5%)<br>IV (0%)	Srg, M: 100%<br>Srg, R: 100%	3.1 (1.3), ND	Convenience sample<br><br>104 'healthy female volunteers' matched for age (matching method not reported).	SDS	SDS mean score (SD):<br><br>48.5 (11.6)	SDS mean score (SD):<br><br>39.9 (9.1)	-	P<0.001 *	Mean SDS scores in breast cancer survivors were significantly higher in women who had neo adjuvant chemotherapy compared to those who did not.
Amir et al., 2002 [13]<br><br>Israel	Convenience sample<br><br>39 women free of cancer symptoms for ≥3 years and not under active treatment, identified through two hospitals.	I (46%)<br>II (46%)<br>III (8%)	Srg, C: 20%<br>Srg, M: 80%<br>CT: 66%<br>RT: 41%<br>HT: 46%	6.5 (ND), ≥5	Convenience sample<br><br>39 women who did not experience any life-threatening disease; matched for age and education (matching method not reported).	SCL-90	SCL-90 mean score (SD):<br><br>0.99 (1.07)	SCL-90 mean score (SD):<br><br>0.66 (0.55)	-	P<0.001 *	Higher SCL-90 scores indicate more depressive symptoms.<br><br>Women who had breast cancer and reported PTSD symptoms had more depressive symptoms than those who did not: 2.13 (1.22) vs. 0.75 (0.75), P<0.01.

ATC = Anatomic Therapeutic Chemical classification system; BC = breast cancer; BDI = Beck Depression Inventory [48]; BDI-II = Beck Depression Inventory-II [49]; BSI-18 = Brief Symptom Inventory-18 [22]; CAD = Clinical Assessment of Depression [50]; CES-D = The Center for Epidemiologic Studies, Depression Scale [51]; CT = chemotherapy; EHR = electronic health records; GDS = Geriatric Depression Scale [52]; HADS = Hospital Anxiety and Depression Scale [23]; HRS-D = Hamilton Rating Scale for Depression [53]; HSCL-25 = Hopkins Symptom Checklist-25 [25]; HT = hormone therapy; ICD-8 = The International Classification of Diseases, Eight Revision; ICD-9-CM = The International Classification of Diseases, Ninth Revision, Clinical Modification; ICD-10 = The International Statistical Classification of Diseases and Related Health Problems, Tenth Revision; ND = not defined; OR = odds ratio; PHQ-D = Patient Health Questionnaire - Depression [54]; PR = prevalence ratio; RR = relative risk; RT = radiotherapy; SCL-90 = Depression subscale of Symptoms Checlist-90 [27]; SD = standard deviation; SDS = Zung's self-rating depression scale [55]; SIR = standardised incidence ratio; Srg, C = Breast conserving surgery; Srg, ND = Surgery, not further specified; Srg, M = Mastectomy; Srg, R = Breast reconstructive surgery; yrs = years.
* There was some statistical evidence (P<0.05) for a different prevalence, risk or severity of anxiety between breast cancer survivors and women who did not have cancer.
† Prevalence ratio calculated by the authors of the present study.

Supplementary Table 5. Neurocognitive dysfunction: main characteristics and results of the studies that evaluated the cognitive dysfunction or its domains in breast cancer survivors (>1 year) and women who did not have cancer.

First author, year of publication<br><br>Country	Breast cancer survivors: Type of population and main characteristics	Breast cancer survivors: Stage at diagnosis (%)	Breast cancer survivors: Breast cancer treatments (%)	Breast cancer survivors: Time since diagnosis/ treatment in years: mean/median (SD), range	Comparison group: Type of population and main characteristics	Outcome assessment	Quantitative measure of the outcome: Breast cancer survivors	Quantitative measure of the outcome: Comparison group	Relative risk estimate (RR, OR, SIR, PR)	P-value or 95% confidence interval	Notes
**Cohort studies involving neurocognitive assessment batteries**											
Ahles et al., 2010 [56]<br><br>United States	Convenience sample<br><br>46 women, aged 18-70 years, newly diagnosed with breast cancer, without history of neurologic disorders or axis I psychiatric disorders, consecutively recruited from one centre.	0 (16.7%)<br>I (47.0%)<br>II (28.0%)<br>IIIA (8.3%)	CT: 100%	~1.5<br><br>(follow up at 18 months after treatment)	Convenience sample<br><br>39 women without cancer recruited through community advertisements; frequency matched for age and education (categories of matching not reported).	Change in the standardised scores for processing speed since baseline assessment prior to CT.<br><br>Processing speed: Digit Symbol-Coding (WAIS-III), Trail Making Test (D-KEFS), Color-Word Interference Test (D-KEFS), and Grooved Pegboard.<br><br>Verbal ability: Vocabulary [WASI, Verbal Fluency Test (D-KEFS)].<br><br>Verbal memory: CVLT-II, Logical Memory I and II (WMS-III).<br><br>Visual memory: Faces I and II (WMS-III).<br><br>Working memory: PASAT.<br><br>Sorting: Sorting Test (D-KEFS).<br><br>Distractibility: CPT.<br><br>Reaction time: CPT.	Mean score (SD)<br><br>Processing speed<br>-0.01 (0.45)<br>Verbal ability<br>0.17 (0.87)<br>Verbal memory<br>0.68 (0.80)<br>Visual memory<br>1.04 (0.69)<br>Working memory<br>0.69 (0.65)<br>Sorting<br>0.52 (0.91)<br>Distractibility<br>0.20 (0.45)<br>Reaction time<br>-0.57 (1.14)<br>Block design<br>0.11 (0.84)	Mean score (SD)<br><br>Processing speed<br>0.25 (0.52)<br>Verbal ability<br>0.17 (0.71)<br>Verbal memory<br>0.69 (0.69)<br>Visual memory<br>1.05 (0.80)<br>Working memory<br>0.64 (0.92)<br>Sorting<br>0.55 (0.73)<br>Distractibility<br>0.16 (0.81)<br>Reaction time<br>0.16 (0.88)<br>Block design<br>0.18 (0.76)	-	-	Domain scores adjusted for age, education, and baseline score.<br><br>The linear mixed-methods model indicated that older patients who received chemotherapy had lower post-treatment processing speed performance (z-score difference,-0.16 per 10 years increase in age; 95%CI: -0.29 to -0.04) compared with healthy controls.
	Convenience sample<br><br>64 women, aged 18-70 years, newly diagnosed with breast cancer, without history of neurologic disorders or axis I psychiatric disorders, consecutively recruited from one centre.	0 (16.7%)<br>I (47.0%)<br>II (28.0%)<br>IIIA (8.3%)	CT: 0%	~1.5<br><br>(follow up at 18 months after treatment)	Convenience sample<br><br>39 women without cancer recruited through community advertisements; frequency matched for age and education (categories of matching not reported).		Mean score (SD)<br><br>Processing speed<br>-0.09 (0.65)<br>Verbal ability<br>-0.04 (0.73)<br>Verbal memory<br>0.38 (0.93)<br>Visual memory<br>1.02 (0.71)<br>Working memory<br>0.44 (0.95)<br>Sorting<br>0.21 (0.86)<br>Distractibility<br>-0.02 (1.05)<br>Reaction time<br>-0.28 (0.95)<br>Block design<br>-0.07 (0.82)	Mean score (SD)<br><br>Processing speed<br>0.25 (0.52)<br>Verbal ability<br>0.17 (0.71)<br>Verbal memory<br>0.69 (0.69)<br>Visual memory<br>1.05 (0.80)<br>Working memory<br>0.64 (0.92)<br>Sorting<br>0.55 (0.73)<br>Distractibility<br>0.16 (0.81)<br>Reaction time<br>0.16 (0.88)<br>Block design<br>0.18 (0.76)	-	-	Domain scores adjusted for age, education, and baseline score.<br><br>The linear mixed-methods model indicated that older patients not exposed to chemotherapy had lower post-treatment Processing Speed performance (z-score difference, -0.11; 95%CI, -0.21 to -0.001).

Collins et al., 2014 [57]<br><br>Canada	Convenience sample<br><br>60 women, aged 18-65 years, with at least the 8th grade of education, newly diagnosed with non-metastatic breast cancer, scheduled to receive CT, recruited in one hospital; patients who had disease progression during follow up were excluded.	I-III (100%)	CT: 100%	~ 1<br><br>(follow up at 12 months after CT)	Convenience sample<br><br>60 women recruited through hospital advertisements and peer nomination, with at least the 8th grade of education; matched on age, education and first language (categories of matching not reported).	Processing Speed:<br>Digit-Symbol Coding & Symbol Search (WAIS-III); TMT-A; TMT-B; Processing speed & Reaction time indices (CNS-VS).<br>Working Memory:<br>Digit Span & Letter-Number-Sequencing (WAIS-III); PASAT; ACTT; COWA; Flexibility & working memory indices (CNS-VS).<br>Visual Memory<br>Visual memory index (CNS-VS).<br>Verbal Memory<br>HVLT-; verbal memory index (CNS-VS).	Prevalence: 22%	Prevalence: 6%	PR= 3.67* †	95%CI: 1.21-11.12	Cut off for case:<br>A standardised-regression based score of ≥ -2.0 on 3 or more of the 19 cognitive measures
Fan et al., 2005 [58]<br><br>Canada	Convenience sample<br><br>91 women with breast cancer without relapse, with no psychiatric history and no use of psychotropic medications other than benzodiazepines for nausea, sleep, or anxiety.	ND	CT: 100%<br>RT: 65%<br>HT: 67%	~ 1<br><br>(follow up at 1 year after CT)	Convenience sample<br><br>102 healthy women, acquaintances or relatives of the patients; individual matching for age (± 5 years).	HSCS, mild dysfunction	Prevalence: 30.8%	Prevalence: 19.3%	PR= 1.60 †	95%CI: 0.93-2.73	-
						HSCS, moderate to severe dysfunction	Prevalence: 4.4%	Prevalence: 3.6%	PR= 1.22 †	95%CI: 0.28-5.31	
						TMT-A	Median score: 44.0	Median score: 45.0	-	P= 0.25	
						TMT-B	Median score: 49.0	Median score: 54.0	-	P= 0.0005 *	
				~ 2<br><br>(follow up at 2 year after CT)		HSCS, mild dysfunction	Prevalence: 21.3%	Prevalence: 11.1%	PR= 1.92 †	95%CI: 0.91-4.04	
						HSCS, moderate to severe dysfunction	Prevalence: 3.8%	Prevalence: 0.0%	PR= 3.88 †	95%CI: 0.33-28.77	
						TMT-A	Median score: 47.0	Median score: 49.0	-	P= 0.61	
						TMT-B	Median score: 50.0	Median score: 53.0	-	P= 0.048 *	
Hermelink et al., 2017 [36]<br><br>Germany	Convenience sample<br><br>56 women with breast cancer, aged 18-65 years, with no history of neurological disorders and no previous systemic treatment.	0 (7%)<br>I (42%)<br>II (41.4%)<br>III (%9.6)	CT: 100%<br>HT: 73.9%<br>vs.<br>CT: 0%<br>HT: 80.7%	~ 1<br><br>(follow up at 1 year after diagnosis)	Convenience sample<br><br>150 women aged 18-65 years, who never had cancer, and attended the same institution as cases for breast imagining and did not require further tests.	Attention<br>TAP; TMT-A,<br><br>Memory<br>Digit span (WSM-R); VLMT.<br><br>Executive function<br>TMT-B; lexical and semantic search (RWT).	Composite z-score:<br>No CT: 0.04 (0.45); CT: -0.10 (0.42)	Composite z-score:<br>0.10 (0.38)	-	P= 0.01 *	Composite score of overall performance calculated as the mean across all age- and education-adjusted cognitive indices (age and education categories in the models not reported). Cognitive change scores were further adjusted for cognitive scores at baseline.
							Composite score, change in the first year of diagnosis:<br>No CT: -0.01 (0.38)<br>CT: -0.07 (0.37)	Composite score, change in the first year of diagnosis<br>0.11 (0.35)	-	P=0.02 *	
							Prevalence: 17.7%	Prevalence: 5.3%	RR= 2.43	95% CI: 0.89 - 6.65	≥5 scores below 1.5 SD and/or ≥4 scores below 2 SD.

Jenkins et al., 2006 [59]<br><br>United Kingdom	Convenience sample<br><br>128 women diagnosed with early breast cancer across the UK, with no disease progression	'early breast cancer'	Srg, M: 26%<br>CT: 66.4%	~ 1<br><br>(follow up at 12 months after CT)	Convenience sample<br><br>49 healthy women who were friends or family of the patients, or from the local women's support group	Verbal memory<br>Logical memory (WMS); Immediate & delayed recall (AVLT).<br>Visual memory<br>Complex figure task.<br>Executive function<br>The Stroop task<br>Working memory<br>Spatial span, letter/number sequencing & digit span (WMS-III)<br>Processing speed<br>Letter cancellation task.	Prevalence of decline on ≥2 measures as measured by the reliable change index:<br><br>16.8%	Prevalence of decline on ≥2 measures as measured by the reliable change index:<br><br>10.6%	PR= 1.58 †	95%CI: 0.64-3.90	Reliable change index corrected for practice effects.
Phillips et al., 2012 [60]<br><br>United States	Convenience sample<br><br>129 women diagnosed with breast cancer and scheduled to receive CT or RT; patients with recurrence were excluded	0 (10%)<br>I (53%)<br>II (37%)	Srg, M: 91.5%<br>Srg, C: 8.5%<br>HT: 62%	3<br><br>(follow up at 26 months after RT)	Convenience sample<br><br>184 women with no history of cancer, individual matching for age (±5 years) and ZIP code.	Attention<br>Trial 1 Color Trails Test; Digit & Spatial Span (WAIS-III).<br><br>Executive functioning<br>Digit Symbol Coding (WAIS-III); Trial 2 Color Trails Test; COWAT.<br><br>Nonverbal memory<br>Visual Reproduction test (WMS-III).<br><br>Processing speed<br>Ruff 2 & 7 Test.<br><br>Verbal memory<br>CVLT.	Score Means (SE)<br>CT group:<br>Attention<br>53.55 (0.72)<br>Executive functioning<br>51.87 (0.81)<br>Nonverbal memory<br>56.24 (0.95)<br>Processing speed<br>49.90 (0.84)<br>Verbal memory<br>50.67 (1.11)<br>RT group:<br>Attention<br>51.59 (0.68)<br>Executive functioning<br>52.30 (0.77)<br>Nonverbal memory<br>54.97 (0.90)<br>Processing speed<br>49.03 (0.80)<br>Verbal memory<br>50.75 (1.05)	Score Means (SE)<br><br>Attention<br>51.78 (0.41)<br>Executive functioning<br>54.63 (0.46)<br>Nonverbal memory<br>55.90 (0.54)<br>Processing speed<br>51.38 (0.48)<br>Verbal memory<br>51.26 (0.63)	-	P<0.05 *	Score means are adjusted for age, T1 National Adult Reading Test scores, and time from T1 to T2 assessments.<br><br>Significant group x time interaction detected for processing speed (P=0.009).
Schagen et al., 2006 [61]<br><br>The Netherlands	Convenience sample<br><br>57 women who had breast cancer treated with RT but not CT, and no relapse	I (100%)	RT: 100%<br>CT: 0%<br>HT: 0%	~1	Convenience sample<br><br>60 healthy women, friends of the participants in the study	24 test indices, covering the following domains: focused-sustained attention, working-verbal-visual memory, processing speed, executive function, and verbal/motor function	Prevalence: 22.8%	Prevalence: 6.7%	OR= 2.1	95%CI: 0.5-8.4	Odds ratio adjusted for age and IQ.<br><br>Cognitive impairment defined as scoring 2 SD below the mean of the control group for ≥3 of the 24 tests.

Cross-sectional studies involving neurocognitive assessment batteries											
Boele et al., 2015 [11]<br><br>The Netherlands	Convenience sample<br><br>Post-menopausal breast cancer survivors with no psychiatric history, who did not receive CT, selected from medical records.	ND	Srg, ND: 95%<br>CT: 0%<br>RT: 65%<br>HT: 100% / 0%	Exposure to HT: 3.2 (1.9), 1.5-7;<br><br>Unexposed to HT: 2.8 (0.3), 2.3-3.3.	Convenience sample<br><br>44 friends or family members of the women who had had breast cancer, with no history of breast cancer; matched for age and education (method of matching not reported).	Verbal memory<br>AVLT; Visual association test.<br><br>Visual memory<br>WMS.<br><br>Working memory<br>Letter-number sequencing (WAIS-III)<br><br>Executive functioning<br>Stroop; TMT-B.<br><br>Processing speed<br>Stroop; TMT-A<br><br>Reaction speed<br>Fepsy reaction times<br><br>Fluency<br>Category fluency, letter fluency<br><br>Motor functioning<br>Fepsy tapping	Domain z-scores by treatment group:<br><br>Verbal memory<br>HT: -0.49 (0.66)<br>Srg+RT: -0.01 (0.63)<br><br>Visual memory<br>HT: 0.136 (0.80)<br>Srg+RT: -0.25 (1.09)<br><br>Working memory<br>HT: -0.144 (0.82)<br>Srg+RT: 0.08 (1.06)<br><br>Executive functioning<br>HT: -0.10 (0.92)<br>Srg+RT: 0.07 (0.93)<br><br>Processing speed<br>HT: -0.06 (0.65)<br>Srg+RT: -0.01 (0.82)<br><br>Reaction speed<br>HT: 0.24 (0.79)<br>Srg+RT: -0.12 (1.07)<br><br>Fluency<br>HT: -0.41 (0.78)<br>Srg+RT: -0.31 (0.70)<br><br>Motor functioning<br>HT: 0.29 (0.70)<br>Srg+RT: 0.14 (0.84)	Domain z-scores:<br><br>Verbal memory<br>-0.001 (0.81)<br><br>Visual memory<br>0.000 (0.95)<br><br>Working memory<br>0.001 (1.00)<br><br>Executive functioning<br>0.000 (0.88)<br><br>Processing speed<br>0.000 (0.79)<br><br>Reaction speed<br>0.000 (0.91)<br><br>Fluency<br>0.000 (0.88)<br><br>Motor functioning<br>0.000 (0.96)	-	Verbal memory<br>P=0.009 *<br><br>Visual memory<br>P=0.339<br><br>Working memory<br>P=0.965<br><br>Executive functioning<br>P=0.444<br><br>Processing speed<br>P=0.554<br><br>Reaction speed<br>P=0.529<br><br>Fluency<br>P=0.012 *<br><br>Motor functioning<br>P=0.667	P-value for the three-group comparison.<br><br>Z-scores corrected for age and estimated premorbid IQ.
Brezden et al., 2000 [62]<br><br>Canada	Convenience sample<br><br>40 women who had completed CT for breast cancer, at least the 8th grade of education, with no history of cognitive dysfunction or psychiatric illnesses and with no clinical evidence of recurrence or metastases.	I-II (ND)	CT (100%)	2 (ND), >1	Convenience sample<br><br>36 healthy female relatives of the patients or hospital personnel who volunteered for the study.	HSCS	Median score: 34.5	Median score: 26.0	-	P>0.05	When adjusted for age, menopausal status, and level of education (categories not reported), the difference was significant (P=0.046).
							Prevalence of moderate and severe cognitive impairment: 50%	Prevalence of moderate and severe cognitive impairment: 11%	PR= 4.5 * †	95%CI: 1.71-12.11	-

Calvio et al., 2010 [7]<br><br>United States	Convenience sample<br><br>122 breast cancer survivors ≥1 year post treatment, working full-time for ≥1 year, with computer and internet, recruited via advertisements and flyers.	I (36.9%)<br>II (44.3%)<br>III (17.2%)	Srg, ND: 96.7%<br>CT: 82.8%<br>RT: 73.0%<br>HT: 45.9%<br>IT: 13.1%	3.1 (2.4), 1-10	Convenience sample<br>113 women without a previous cancer diagnosis, working full-time for ≥1 year, with computer and Internet, recruited via advertisements and flyers.	CNS-VS battery<br><br>Composite memory<br>Verbal memory<br>Visual memory<br>Executive function<br>Attention	Composite memory: 101.7 (18.1)<br><br>Verbal memory: 99.8 (16.6)<br><br>Visual memory: 102.8 (17.1)<br><br>Executive function: 98.6 (9.2)<br><br>Attention: 83.8 (10.3)	Composite memory: 97.1 (19.8)<br><br>Verbal memory: 96.0 (20.0)<br><br>Visual memory: 99.3 (17.1)<br><br>Executive function: 94.5 (16.4)<br><br>Attention: 80.2 (17.7)	-	Executive function: P<0.001 *<br><br>Attention: P<0.05 *<br><br>All other domains P>0.05	Lower scores indicate poorer functioning.<br><br>Mean scores adjusted for marital status (cohabitating with partner vs. single/not cohabitating), race (Caucasian vs. non-Caucasian), ethnicity (Hispanic vs. non-Hispanic), age (<40, 41-50, 51-65), income (0-39,000; 40-59,000; 60-79,000; 80-89,000; 80-99,000; ≥100,000), and menopausal status (currently going through, premenopausal, postmenopausal).
Castellon et al., 2004 [15]<br><br>United States	Convenience sample<br><br>53 women who had breast cancer at or before the age of 50, with no evidence of disease or recurrence, and no history of psychiatric disorder.	0-II (100%)	CT: 34%<br>CT+HT: 34%	2-5	Convenience sample<br><br>19 Healthy women recruited via fliers, newsletter articles and advertisements, or amongst the acquaintances of the hospital staff.	Verbal Fluency<br>COWA.<br><br>Verbal Learning<br>CVLT.<br><br>Verbal Memory<br>Logical memory (WMS-R).<br><br>Visual Memory<br>Visual Reproduction (WMS-R); RCFT.<br><br>Visuospatial Function<br>Block Design (WAIS-III); Copy Trial (RCFT).<br><br>Psychomotor Speed<br>Digit Symbol (WAIS-III); TMT-A; TMT-B.<br><br>Reaction Time<br>CCAP<br><br>Executive Attention<br>PASAT; Stroop Test.	z-scores, no CT nor HT:<br>Fluency: -0.36<br>Verbal Learning: 0.54<br>Verbal memory: 0.21<br>Visual memory: 0.45<br>Visuospatial: 0.42<br>Reaction time: -0.20<br>Psychomotor speed: 0.22<br>Executive attention: -0.01<br><br>z-scores, CT (with or without HT):<br>Fluency: -0.64<br>Verbal learning: 0.03<br>Verbal memory: -0.35<br>Visual memory: -0.39<br>Visuospatial: -0.51<br>Reaction time: -0.49<br>Psychomotor speed: 0.03<br>Executive attention: -0.41	Ref the mean scores of the healthy women used to calculate the z-scores.	-	Verbal Fluency: P=0.007 *<br><br>All other domains: p>0.05	-

Conroy et al., 2013 [18]<br><br>United States	Convenience sample<br><br>24 breast cancer survivors with history of non-metastatic disease and chemotherapy treated.	I (29%)<br>IIa (33%)<br>IIb (25%)<br>IIIa (8%)<br>IIIb (4%)	CT: 100%<br>RT: 79%	6.4 (2.1), 3.2-10.2	Convenience sample<br><br>23 healthy women; matched for age and education (matching method not reported).	Learning<br>AVLT; BLT.<br><br>Memory<br>AVLT; BLT.<br><br>Attention<br>Digit span (WAIS-III); PASAT.<br><br>Language<br>WRAT-4; Word Reading test; Vocabulary (WASI).<br><br>Visuospatial<br>Block Design (WASI)<br><br>Executive<br>Digit span; COWA; Color-Word Test, Sorting Test, & Trail Making Test (D-KEFS).<br><br>Psychomotor<br>Symbol Digit, and Grooved Pegboard.	Age-adjusted domain z-scores:<br><br>Learning: -0.2 (0.7)<br>Memory: -0.3 (0.6)<br>Attention: 0.4 (0.6)<br>Language: 0.3 (0.8)<br>Visuospatial: -0.5 (1.0)<br>Executive: -0.04 (0.7)<br>Psychomotor: -0.1 (0.4)<br>Average: -0.1 (0.5)	Age-adjusted domain z-scores:<br><br>Learning: 0.1 (0.7)<br>Memory: 0.2 (0.7)<br>Attention: 0.03 (0.5)<br>Language: -0.03 (0.9)<br>Visuospatial: 0.1 (0.9)<br><br>Executive: 0.04 (0.6)<br>Psychomotor: 0.04 (0.4)<br>Average: 0.1 (0.4)	-	Memory: P≤0.05 *<br><br>All other domains: P>0.05	-
Ernst et al., 2002 [63]<br><br>United States	Convenience sample<br>16 women aged 65-80 years, recruited via advertisements.	'localised breast cancer'	HT: 100%<br>Srg, ND: 100%<br>CT: 0%	4.4 (1.7), 2-10	Convenience sample<br>33 women with no history of breast cancer; matched for age (matching method not reported).	Digit symbol substitution test	Nr of correct substitutions (SD):<br>7.5 (3.1)	Nr of correct substitutions (SD):<br>7.2 (2.1)	-	P>0.05	-
						TMT-A	Time required (SD):<br>44.2 (12.2)	Time required (SD):<br>36.9 (10.4)	-	P>0.05	-
Inagaki et al., 2006 [64]<br><br>Japan	Convenience sample<br>105 women who had breast cancer aged 18-55 years, with no history of neurological or psychiatric disorders other than affective or anxiety; tumor free at recruitment.	0-I (27.5%)	Srg, C: 49%<br>CT: 100%<br>HT: 39%<br>RT: 48%	1	Convenience sample<br><br>55 healthy subjects who lived in the same area as the patients recruited via advertisements in the local newspaper; matched for region (matching method not reported).	WMS-R	Mean domain score (SD):<br>Attention<br>99.4 (12.5)<br>Verbal memory<br>96.9 (13.0)<br>Visual memory<br>101.9 (12.1)<br>Delayed recall<br>100.3 (10.4)	Mean domain score (SD):<br>Attention<br>99.6 (13.0)<br>Verbal memory<br>99.2 (14.4)<br>Visual memory<br>101.4 (10.3)<br>Delayed recall<br>100.7 (12.6)	-	For all domains: P>0.05	-

Kesler et al., 2013 [34]<br><br>United States	Convenience sample<br><br>44 women who had breast cancer recruited via support groups and advertisements; patients excluded if they had had disease recurrence or relapse	I-IIIA	Srg, ND: 100%<br>CT: 100%	4.8 (3.4), 1-12	Convenience sample<br><br>38 healthy female controls recruited through advertisements	MMQ; HVLT-R; WAIS	Mean scores (SD):<br>HVLT-R total recall: 49.3 (8.0)<br>HVLT-R delayed recall: 49.8 (6.4)<br>MMQ: 42.2 (11.2)<br>WAIS-IQ: 112 (11)	Mean score (SD):<br>HVLT-R total recall: 57.1 (9.6)<br>HVLT-R delayed recall: 56.0 (8.1)<br>MMQ: 59.3 (7.4)<br>WAIS-IQ: 115 (13)	-	P=0.03 *<br>P=0.02 *<br>P<0.001 *<br>P=0.29	-
Koppelmans et al., 2012 [42]<br><br>The Netherlands	Convenience sample<br><br>196 women who had been treated for breast cancer between 1976 and 1995, were aged 50-80 years in 2008, did not have recurrence or a second primary cancer and never used adjuvant hormone therapy.	I-III (100%)	HT: 0%<br>CT: 100%	21 (4.4), ND	Convenience sample<br><br>All 1,509 women without a history of cancer who were 50-80 years of age at the time of the assessments, selected from a larger population-based cohort.	Learning and memory (15-WLT)	Trial 1: 5.5 (2.2)<br>Trial 2: 8.6 (2.4)<br>Trial 3: 10.3 (2.6)<br>Total: 24.3 (6.2)<br>Delayed recall: 8.0 (2.9)<br>Recognition: 13.8 (1.8)	Trial 1: 5.9 (2.4)<br>Trial 2: 9.0 (2.7)<br>Trial 3: 10.6 (2.9)<br>Total: 25.5 (6.9)<br>Delayed recall: 8.7 (3.2)<br>Recognition: 13.8 (2.0)	-	P=0.008 *<br>P=0.02 *<br>P=0.17<br>P=0.02 *<br>P=0.002 *<br>P=0.76	Adjusted for age and education (categories of the variables used in the models not reported).
						Processing speed (LDST)	Total correct: 31.8 (6.7)	Total correct: 32.5 (7.5)	-	P=0.14	
						Stroop color-word test	Word card: 16.8 (3.3)<br>Color card: 23.3 (4.4)<br>Color-word card: 45.8 (12.6)	Word card: 16.5 (3.7)<br>Color card: 22.2 (4.9)<br>Color-word card: 43.5 (14.0)	-	P=0.14<br>P=0.001 *<br>P=0.02 *	
						Verbal fluency (WTF)	Total: 24.1 (6.1)<br>15sec: 13.8 (4.8)	Total: 24.2 (6.8)<br>15sec: 13.8 (5.4)	-	P=0.89<br>P=0.95	
						Visuospatial (DOT)	Total correct: 28.9 (9.2)	Total correct: 28.9 (9.7)	-	P=0.99	
						Motor speed (PPB)	Both hands: 11.1 (1.6)<br>Dominant hand: 13.8 (1.9)<br>Nondominant hand: 12.9 (1.8)	Both hands: 11.2 (1.8)<br>Dominant hand: 13.8 (2.1)<br>Nondominant hand: 13.4 (2.0)	-	P=0.56<br>P=0.81<br>P=0.001 *	
Kreukels et al., 2008 [12]<br><br>The Netherlands	Convenience sample<br><br>63 women who had been treated with CT for non-metastatic breast cancer, with no history of psychiatric diseases	I-III (100%)	CT: 100%<br>HT: 40%	~ 1	Convenience sample<br><br>60 Female friends or relatives of the patients with the same approximate age who never had cancer; matched for age (matching method not reported).	TMT-A; Digit Symbol (WAIS); Stroop Color Word Test; Eriksen Task, Working-Memory Updating, CVLT, Visual Reproduction of the WMS, AFM Task, TMT-B, Word Fluency, Fepsy Finger Tapping.	Prevalence of cognitive impairment: 33.3%	Prevalence of cognitive impairment: 10%	RR= 5.51 *	95%CI: 1.86-16.28	Cognitive impairment defined as 2 standard deviations below the mean of the healthy control group on ≥ 3 tests.<br><br>RR adjusted for age and premorbid IQ.

Lejbak et al., 2010 [65]<br><br>Canada	Convenience sample<br><br>28 post menopausal women with oestrogen positive breast cancer, aged 40 and 80 years, recruited from the local cancer registry and oncology centre	I (100%)	HT: 100%<br>Srg, ND: 83%<br>RT: 67%	3 (1), 2-5	Convenience sample<br><br>37 age-equivalent controls recruited through mailed invitations	Immediate verbal memory: List Learning, Story Memory.<br>Delayed verbal memory List Recall, Story Recall.<br>Complex visuomotor attention: Coding<br>Letter fluency: COWA<br>Object location memory task<br>Speeded manual dexterity: Grooved Pegboard<br>Complex working memory Verbal n-Back	Mean score (SD):<br>List Learning 29.0 (5.1)<br>List Recall 7.1 (2.2)<br>Story Memory 17.1 (3.6)<br>Story Recall 9.0 (2.3)<br>Coding 42.9 (9.5)<br>Letter Fluency 40.0 (10.8)<br>Object-Location 47.5 (21.1)<br>Grooved Pegboard 80.9 (17.1)<br>Verbal n-Back 119.9 (9.7)	Mean score (SD)<br>List Learning 30.3 (3.8)<br>List Recall 6.8 (2.2)<br>Story Memory 18.4 (3.3)<br>Story Recall 9.7 (2.1)<br>Coding 49.3 (9.2)<br>Letter Fluency 44.3 (11.2)<br>Object-Location 44.4 (20.0)<br>Grooved Pegboard 67.76 (12.7)<br>Verbal n-Back 123.0 (9.5)	-	P=0.24<br>P=0.58<br>P=0.15<br>P=0.23<br>P=0.01 *<br>P=0.03 *<br>P=0.55<br>P<0.01 *<br>P=0.23	Higher scores indicate better performance.
Miao et al., 2016 [9]<br><br>China	Convenience sample<br><br>23 patients with breast cancer who had been treated with chemotherapy at a local hospital	I-III (100%)	CT: 100%	3 (0.3),	Convenience sample<br><br>26 age matched healthy controls selected amongst patients relatives and local universities; matched for age (matching method not reported).	Stroop interference test; MoCA	Mean score (SD)<br>Stroop: 35.04 (8.96)<br>MoCA: 26.00 (1.34)	Mean score (SD)<br>Stroop: 30.17 (6.49)<br>MoCA: 26.58 (1.74)	-	P=0.04 *<br>P>0.05	Higher score in the Stroop interference test indicates worse performance.
Myers et al., 2015 [66]<br><br>United States	Convenience sample<br><br>156 breast cancer patients recruited across 24 states using newsletters and flyers	I (26%)<br>II (47%)<br>III (14%)<br>IV (5%)	CT: 100%<br>RT: 71.2%<br>HT: 49.4%	1-2	Convenience sample<br><br>46 healthy controls recruited using flyers	FACT-COG,<br>Perceived cognitive impairments (PCI)<br>Perceived cognitive abilities (PCA)	Mean score (SD):<br>PCI: 48.6 (17.2)<br>PCA: 17.6 (7.2)	Mean score (SD):<br>PCI: 61.1 (9.4)<br>PCA: 19.1 (8.8)	-	P<0.05 *<br>P>0.05	Higher scores indicate higher cognitive function.
				2-5			PCI: 41.7 (18.3)<br>PCA: 15.9 (6.8)	PCI: 61.1 (9.4)<br>PCA: 19.1 (8.8)	-	P<0.05 *<br>P<0.05 *	
				>5			PCI: 50.4 (18.2)<br>PCA: 19.0 (6.9)	PCI: 61.1 (9.4)<br>PCA: 19.1 (8.8)	-	P<0.05 *<br>P<0.05 *	

Nguyen et al., 2013 [39]<br><br>United States	Convenience sample<br><br>57 women survivors of breast cancer, aged over 65 years, without recurrence, recruited from the cancer registry	I-IIIA (100%)	RT: 53%<br>CT: 100%	>10	Convenience sample<br><br>30 healthy female adults, selected in the community for a previous study	Intelligence and mental status<br>WASI; Wide Range Achievement Test-III reading subtest;<br>Folstein mini mental state examination.<br><br>Attention and working memory<br>Digit Span, Letter-Number Sequencing, and Arithmetic subtests (WAIS-III)<br><br>Psychomotor speed<br>TMT-A.<br><br>Language<br>COWA; Boston Naming Test<br><br>Visuospatial<br>RCFT-Copy Condition; Benton Facial; Recognition Test.<br><br>Memory<br>AVLT; RCFT-Delay Condition.<br>Benton Visual Retention Test-Revised.<br>Executive functioning<br>Wisconsin Card Sorting Test. TMT-B.	WASI<br>Vocabulary:<br>64.5 (7.8)<br>Block design:<br>33.9 (12.3)<br>Similarities:<br>36.8 (4.8)<br>Matrix design:<br>20.6 (6.5)<br>Wide Range Achievement Test-III Reading: 48.1 (4.7)<br>Digit span: 15.5 (3.4)<br>Letter–Number Seq 9.1 (2.1)<br>Arithmetic total 12.4 (2.8)<br>TMT<br>A time: 37.8 (8.9)<br>B-time: 97.0 (35.5)<br>COWA: 39.4 (15.1)<br>Boston Naming Test 57.0 (2.5)<br>Rey–Osterrieth Complex Figure<br>Copy: 33.3 (2.0)<br>Delay: 15.9 (5.1)<br>Benton Faces total 44.4 (3.4)<br>Benton Visual Retention Test total 5.3 (2.2)<br>Rey Auditory-Verbal Learning Test<br>Total: 48.6 (8.3)<br>Delay: 10.2 (2.6)<br>IED: 3.2 (4.2)<br>Wisconsin:<br>Perseverative: 12.5 (6.9)<br>Errors: 11.0 (5.7)<br>Categories: 2.9 (1.6)	WASI<br>Vocabulary:<br>63.7 (6.9)<br>Block design:<br>34.8 (11.9)<br>Similarities:<br>36.6 (3.0)<br>Matrix design:<br>21.8 (6.8)<br>Wide Range Achievement Test-III Reading: 50.2 (5.0)<br>Digit span: 16.9 (4.4)<br>Letter–Number Seq 11.0 (2.0)<br>Arithmetic total 13.7 (3.2)<br>TMT<br>A time: 29.3 (8.7)<br>B-time: 72.4 (26.6)<br>COWA: 38.8 (11.1)<br>Boston Naming Test 56.1 (3.0)<br>Rey–Osterrieth Complex Figure<br>Copy: 32.0 (2.9)<br>Delay: 15.6 (5.6)<br>Benton Faces total 45.5 (3.8)<br>Benton Visual Retention Test total 4.4 (2.8)<br>Rey Auditory-Verbal Learning Test<br>Total: 49.4 (8.7);<br>Delay: 10.6 (2.3).<br>IED: 1.3 (1.0)<br>Wisconsin:<br>Perseverative: 16.6 (12.2)<br>Errors: 14.9 (11.0)<br>Categories: 5.0 (1.9)	-	P<0.05 *:<br>Letter–Number Seq; Trail making test A and B; Boston naming test; Rey–Osterrieth Complex Figure; Benton Visual Retention; Rey Auditory-Verbal Learning; IED; Wisconsin Card Sorting categories.<br><br>P>0.05<br>All other tests.	-

Root et al., 2015 [17]<br><br>United States	Convenience sample<br><br>113 women aged <70 years who had breast cancer, post-menopausal at diagnosis, with no recurrence, no neurological or psychiatric diagnoses.	I (58%)<br>II (0%)<br>III (33%)<br>IV (8%)	Srg, C: 75%<br>Srg, M: 32%<br>CT: 52%<br>RT: 78%<br>HT: 52%	4.2 (1.2)	Convenience sample<br><br>37 health women with no history of cancer or cancer treatment, post-menopausal, with no neurological or psychiatric diagnoses; matched for age and education (method of matching not reported).	FACT-COG	Mean score (SD)<br>Memory: 20.4 (5.9)<br>Verbal 18.5 (4.8)<br>Concentration 12.4 (3.2)<br>Mental acuity 12.0 (3.4)<br>QoL impact 13.7 (3.0)<br>PCI: 56.5 (12.7)<br>PCA: 19.5 (6.3)	Mean score (SD):<br>Memory: 23.5 (3.2)<br>Verbal: 19.2 (3.6)<br>Concentration: 13.6 (2.4)<br>Mental acuity: 13.4 (2.0)<br>QoL impact: 14.3 (2.4)<br>PCI: 59.4 (8.3)<br>PCA: 22.7 (4.5)	-	P=0.003 *<br>P=0.42<br>P=0.04 *<br>P=0.02 *<br>P=0.27<br>P=0.20<br>P=0.005 *	-
Silverman et al., 2007 [67]<br><br>United States	Convenience sample<br><br>24 women who had breast cancer and were right handed.	ND	CT+HT: 52%<br>CT: 24%	ND (ND), 5-10	Convenience sample<br><br>10 healthy controls who had undergone PET studies before, free of cognitive impairments.	RCFT- recall test	Mean (SD):<br><br>20.6 (4.8)	Mean (SD):<br><br>23.8 (6.3)	-	P>0.05	Lower scores represent worse functioning.
Von Ah et al., 2009 [44]<br><br>United States	Convenience sample<br><br>52 women aged ≥40 years, who had breast cancer, recruited from cancer support groups, advertisements in the community centres, or by referral of enrolled participants.	I-II (50%)<br>III (ND)	Srg, C: 66%<br>Srg, M: 33%<br>CT: 55.8%<br>RT: 80.8%<br>HT: 79%	4.6 (2.8), 1.2-15.8	Convenience sample<br><br>52 women aged ≥40 years, with no history of cancer, no history of psychiatric illnesses, recruited from advertisements in churches and community centres, or by referral of enrolled participants; individual matching for age (±5 years) and education (±3 years).	Memory:<br>AVLT	Sum recall: 48.5 (7.2)<br>Delayed recall: 9.6 (2.8)	Sum recall: 52.4 (8.1)<br>Delayed recall: 10.9 (2.8)	-	P=0.01 *	-
						Attention:<br>Digit span (WAIS-III)	17.8 (4.0)	17.7 (4.1)	-	P=0.89	-
						Attention:<br>Symbol digit modalities test	53.6 (8.2)	54.1 (10.4)	-	P=0.79	-
						Executive function:<br>COWA	38.2 (10.9)	42.2 (12.4)	-	P=0.08	-
						Subjective memory function: Squire SRS	92.9 (17.9)	102.9 (22.6)	-	P=0.01 *	-

ACTT = Auditory Consonant Trigrams Test [68]; AFM = Additive factors method task [69]; AVLT = Rey Auditory Verbal Learning Test [70]; BC = breast cancer; BLT = Brown Learning Test [71]; CCAP - California Computerized Assessment Package [72]; CNS-VS = CNS vital signs battery [73, 74]; COWA = Controlled Oral Word Association [75]; CPT = Continuous Performance test [76]; CT = chemotherapy; CVLT = California Verbal Learning Test [77]; D-KEFS = Delis-Kaplan Executive Function System [78]; DOT = Design organization test [79]; EORTC-QLQ-CF = the European Organization for Research and Treatment of Cancer [80]; FACT-COG = Functional Assessment of Cancer Therapy for Cognition [81]; HSCS = High Sensitivity Cognitive Screen [82]; HT = hormone therapy; HVLT-R = Hopkins verbal learning test revised [83]; IT = immunotherapy; LDST = Letter Digit Substitution Test [84]; MoCA = Montreal Cognitive Assessment Test [85]; Multifactorial Memory Questionnaire Ability Scale [86]; ND = not defined; OR = odds ratio; PASAT = Paced Auditory Serial Addition Test [87]; PCA = Perceived cognitive abilities; PCI = Perceived cognitive impairments; PPB = Purdue Pegboard test [88]; PR = prevalence ratio; RCFT = Rey-Osterrieth Complex Figure Test, Copy Condition [89-91]; RT = radiotherapy; RWT = Regensburg word fluency test [92]; SD = standard deviation; Srg, C = Breast conserving surgery; Srg, ND = Surgery, not further specified; Srg, M = Mastectomy; Srg, R = Breast reconstructive surgery; SRS = Squire self-report scale [93]; TAP = Test of Attentional Performance [94]; TMT-A = Trail Making Test-A [95]; TMT-B = Trail Making Test-B [95]; WAIS-III = Wechsler Adult Intelligence Scale-III [96]; WASI = Wechsler Abbreviated Scale of Intelligence [97]; 15-WLT = 15-Word Learning Test [98]; WMS-R = Wechsler Memory Scale-Revised [99]; WRAT = Wide Range Achievement Test [100]; WTF = Word Fluency Test [101]; yrs = years; 95%CI = 95% confidence interval.

* There was some statistical evidence ($P<0.05$) for a different prevalence, risk or severity of anxiety between breast cancer survivors and women who did not have cancer.
† Prevalence ratio calculated by the authors of the present study.

Supplementary Table 6. Sexual dysfunction: main characteristics and results of the studies that provided data on the frequency and/or severity of sexual dysfunction in breast cancer survivors (>1 year) and women who did not have cancer.

First author, year of publication<br><br>Country	Breast cancer survivors: Type of population and main characteristics	Breast cancer survivors: Stage at diagnosis (%)	Breast cancer survivors: Breast cancer treatments: %	Breast cancer survivors: Time since diagnosis/ treatment in years: mean/ median (SD), range	Comparison group: Type of population and main characteristics	Outcome assessment	Prevalence / cumulative incidence of the outcome: Breast cancer survivors	Prevalence / cumulative incidence of the outcome: Comparison group	Relative risk estimate (RR, OR, SIR, PR)	P-value or 95% confidence interval	Notes
Cross-sectional studies											
Boehmer et al., 2014 [102]<br><br>United States	Convenience sample<br><br>85 lesbian or bisexual breast cancer survivors, with no metastatic breast cancer or secondary cancers, recruited via advertisements, flyers, and other promotional materials distributed online and in print media (<5% had cancer recurrence).	0 (16.5%)<br>I (28.2%)<br>II (37.7%)<br>III (8.2%)<br>Unknown (9.4%)	Srg C: 41.2%<br>Srg, M: 40.0%<br>CT: 61.2%<br>RT: 58.8%<br>HT: 45.9%	4.5 (2.3), 1-10	Convenience sample<br>85 lesbian or bisexual women with no history of cancer, not using hormone replacement therapy, recruited via flyers, advertisements, etc.; individual matching for age (± 3 years) and partner status (partnered vs. unpartnered).	Scale: FSFI	Prevalence: 52.5%	Prevalence: 4.3%	All women:<br>OR=1.44<br><br>Sexually active:<br>OR=1.79	All women:<br>95%CI: 0.72-2.90<br><br>Sexually active:<br>95%CI 0.78-4.07	40% of the cases and 31% of the controls were sexually inactive.
						Scale: FSFI<br>Overall score	Mean score (SD):<br>24.0 (7.2)	Mean score (SD):<br>26.0 (5.3)	-	P=0.08	
						Subscales: Desire	4.3 (2.0)	5.7 (2.2)	-	P<0.01 *	
						Arousal	13.2 (5.3)	14.9 (4.5)	-	P=0.07	
						Lubrication	13.5 (6.0)	11.6 (1.1)	-	P=0.03 *	
						Orgasm	11.1 (4.1)	12.6 (3.2)	-	P=0.04 *	
						Satisfaction	11.0 (3.2)	11.8 (3.2)	-	P=0.22	
						Pain	12.8 (3.2)	14.1 (1.8)	-	P=0.03 *	
Safarinejad et al., 2013 [103]<br><br>Iran<br><br>(continues)	Convenience sample<br><br>186 women cancer survivors aged 25-45, with BMI<30 kg/m^2, in a relationship and attempted intercourse weekly, with no breast cancer recurrence, no other cancer, no psychopathology, no relationship disturbances, no diabetes or cardiac, renal, neurological, or liver disease, among others; identified from the cancer registry.	I (62.4%)<br>II (37.6%)	Srg, C: 100%<br>Srg, M: 0%<br>CT: 67.7%<br>RT: 46.2%<br>HT: 79.6%	2.4 (ND), >1	Convenience sample<br>204 women without cancer aged 25-45 in a relationship, who attempted intercourse weekly, in same geographical area of cases, with BMI<30kg/m^2, no psychopathology, no relationship disturbances, no diabetes or cardiac, renal, neurological, or liver disease, among others, recruited from a private clinic; matched for age (matching method not reported).	Scale: FSFI	Prevalence of dysfunction: 52.5%<br>By treatment:<br>RT+CT: 44.6%<br>CT+HT: 46.2%<br>CT+RT+HT: 66.7%	Prevalence: of dysfunction:<br><br>28.7%	PR=1.81 * †<br>By treatment:<br>PR= 1.55 *<br>PR= 1.61 *<br>OR= 8.2 *	95%CI: 1.40-2.34<br>By treatment:<br>95%CI: 1.13-1.98<br>95%CI: 1.32-1.90<br>95%CI: 6.5-14.2	Odds ration adjusted for age, body mass index, occupational status, educational level, smoking history, serum hormonal levels, tumour stage and grading. The categorization of the variables included in the model were not reported.
						FSFI Subscales:<br>Desire	All treatments: 41.9%<br>By treatment:<br>RT+CT: 33%<br>CT+HT: 42%<br>CT+RT+HT: 53%	28.0%	PR=1.50 * †<br>By treatment:<br>OR= 1.8<br>OR= 3.6 *<br>OR= 4.7 *	95%CI: 1.13-1.98<br>By treatment:<br>95%CI: 0.9-2.2<br>95%CI: 2.6-6.8<br>95%CI: 2.8-8.7	
						Arousal	All treatments: 33.9%<br>By treatment:<br>RT+CT: 31%<br>CT+HT: 30%<br>CT+RT+HT: 50%	25.0%	PR=1.36 †<br>By treatment:<br>OR= 1.6<br>OR= 1.5<br>OR= 4.2 *	95%CI: 0.99-1.85<br>By treatment:<br>95%CI: 0.8-2.8<br>95%CI: 0.8-2.8<br>95%CI: 2.6-8.2	
						Orgasm	All treatments: 41.9%<br>By treatment:<br>RT+CT: 33%<br>CT+HT: 41%<br>CT+RT+HT: 56%	29.0%	PR=1.44 * †<br>By treatment:<br>OR= 2.1<br>OR= 3.2 *<br>OR= 5.2 *	95%CI: 1.10-1.90<br>By treatment:<br>95%CI: 0.8-3.4<br>95%CI: 2.4-7.1<br>95%CI: 3.7-10.2	

Safarinejad et al., 2013 [103]<br><br>Iran<br><br>(continued)	Convenience sample<br><br>186 women cancer survivors aged 25-45, with BMI<30 kg/m^2, in a relationship and attempted intercourse weekly, with no breast cancer recurrence, no other cancer, no psychopathology, no relationship disturbances, no diabetes or cardiac, renal, neurological, or liver disease, among others; identified from the cancer registry.	I (62.4%)<br>II (37.6%)	Srg, C: 100%<br>Srg, M: 0%<br>CT: 67.7%<br>RT: 46.2%<br>HT: 79.6%	2.4 (ND), >1	Convenience sample<br><br>204 women without cancer aged 25-45 in a relationship, who attempted intercourse weekly, in same geographical area of cases, with BMI<30kg/m^2, no psychopathology, no relationship disturbances, no diabetes or cardiac, renal, neurological, or liver disease, among others, recruited from a private clinic; matched for age (matching method not reported).	Pain	All treatments: 39.2%<br>By treatment:<br>RT+CT: 31%<br>CT+HT: 36%<br>CT+RT+HT: 59%	Prevalence:<br><br>30.0%	PR=1.31 †<br>By treatment:<br>OR= 1.2<br>OR= 2.2 *<br>OR= 5.6 *	95%CI: 0.99-1.72<br>By treatment:<br>95%CI: 0.96-1.8<br>95%CI: 1.5-3.8<br>95%CI: 3.2-11.4	Odds ratio adjusted for age, body mass index, occupational status, educational level, smoking history, serum hormonal levels, tumour stage and grading. The categorization of the variables included in the model were not reported.
						Lubrication	All treatments: 58.1%<br>By treatment:<br>RT+CT: 56%<br>CT+HT: 55%<br>CT+RT+HT: 61%	31.0%	PR=1.87 * †<br>By treatment:<br>OR= 4.2 *<br>OR= 4.1 *<br>OR= 6.4 *	95%CI: 1.48-2.38<br>By treatment:<br>95%CI: 3.4-8.7<br>95%CI: 3.2-8.4<br>95%CI: 4.6-12.6	
						Satisfaction	All treatments: 53.8%<br>By treatment:<br>RT+CT: 50%<br>CT+HT: 53%<br>CT+RT+HT: 59%	29.0%	PR=1.86 * †<br>By treatment:<br>OR= 3.4 *<br>OR= 3.8 *<br>OR= 5.7 *	95%CI: 1.44-2.39<br>By treatment:<br>95%CI: 1.8-5.8<br>95%CI: 2.2-6.1<br>95%CI: 3.4-11.4	
						Desire	Mean score (95%CI):<br>3.7 (3.1-4.3)<br>By treatment:<br>RT+CT: 4.4 (3.8-4.7)<br>CT+HT: 3.6 (2.9-4.4)<br>CT+RT+HT: 3.1 (2.6-3.6)	Mean score (95%CI):<br><br>4.8 (3.6-5.6)	-	P<0.05 *<br><br>By treatment:<br>P>0.05<br>P<0.05 *<br>P<0.05 *	Women who had RT+CT+HT reported more sexual dysfunction problems than women who had RT+CT for all domains, and more impairments than women who had CT+HT for arousal, lubrication, satisfaction and pain.
						Arousal	4.0 (3.3-4.3)<br>By treatment:<br>RT+CT: 4.4 (3.6-4.6)<br>CT+HT: 4.3 3.6-4.6()<br>CT+RT+HT: 3.3 (2.7-3.7)	4.9 (3.5-5.4)	-	P<0.05 *<br>By treatment:<br>P>0.05<br>P>0.05<br>P<0.05 *	
						Lubrication	2.8 (2.4-3.3)<br>By treatment:<br>RT+CT: 3.1 (2.6-3.6)<br>CT+HT: 3.1 (2.6-3.5)<br>CT+RT+HT: 2.4 (1.9-2.8)	5.1 (3.5-5.8)	-	P<0.05 *<br>By treatment:<br>P<0.05 *<br>P<0.05 *<br>P<0.05 *	
						Orgasm	3.7 (3.1-4.1)<br>By treatment:<br>RT+CT: 4.3 (3.6-4.7)<br>CT+HT: 3.6 (3.1-3.9)<br>CT+RT+HT: 3.2 (2.7-3.6)	4.7 (3.8-5.8)	-	P<0.05 *<br>By treatment:<br>P>0.05<br>P<0.05 *<br>P<0.05 *	
						Satisfaction	3.3 (2.9-3.7)<br>By treatment:<br>RT+CT: 3.4 (3.0-3.9)<br>CT+HT: 3.5 (3.1-4.0)<br>CT+RT+HT: 2.9 (2.5-3.3)	5.1 (3.7-5.7)	-	P<0.05 *<br>By treatment:<br>P<0.05 *<br>P<0.05 *<br>P<0.05 *	
						Pain	4.6 (3.8-4.7)<br>By treatment:<br>RT+CT: 4.9 (4.5-5.0)<br>CT+HT: 4.4 (4.1-4.6)<br>CT+RT+HT: 3.1 (2.7-3.5)	5.1 (3.8-5.5)	-	P>0.05<br>By treatment:<br>P>0.05<br>P<0.05 *<br>P<0.05 *	

Claus et al., 2006 [41]<br><br>United States	Population-based<br><br>All 795 women in Connecticut diagnosed with DCIS in 1994-1998, with history of invasive breast cancer	0 (100%)	Srg, C: 35.5%<br>Srg, M: 14.0%	5.8 (1.0), ND	Population based<br><br>702 women selected by random-digit-dialling methods, with no history of DCIS or invasive breast cancer; frequency matched by age (± 5 years) and geography.	Scale: MOS-SFS, Lack of interest	Prevalence: 27.9%<br>By treatment:<br>Srg, C: 25.6%<br>Srg, C + RT: 31.0%<br>Srg, M: 22.6%	Prevalence: 22.3%	PR= 1.25 * †<br>By treatment:<br>PR= 1.15 †<br>PR= 1.39 * †<br>PR= 1.01 †	95%CI: 1.05-1.49<br>By treatment:<br>95%CI: 0.90-1.46<br>95%CI: 1.14-1.70<br>95%CI: 0.70-1.47	Cut-off for case: “somewhat of a problem” or “very much of a problem”.
						Unable to relax	Prevalence: 19.2%<br>By treatment:<br>Srg, C: 20.1%<br>Srg, C + RT: 18.6%<br>Srg, M: 18.7%	Prevalence:12.8%	PR=1.50 * †<br>By treatment:<br>PR= 1.57 * †<br>PR= 1.45 * †<br>PR= 1.46 †	95%CI: 1.16-1.91<br>By treatment:<br>95%CI: 1.16-2.12<br>95%CI: 1.10-1.93<br>95%CI: 0.95-2.25	
						Difficulty with arousal	Prevalence: 23.0%<br>By treatment:<br>Srg, C: 25.6%<br>Srg, C + RT: 22.3%<br>Srg, M: 18.7%	Prevalence:15.2%	PR=1.51 * †<br>By treatment:<br>PR= 1.68 * †<br>PR= 1.47 * †<br>PR= 1.23 †	95%CI: 1.22-1.88<br>By treatment:<br>95%CI: 1.29-2.19<br>95%CI: 1.14-1.89<br>95%CI: 0.80-1.87	
						Difficulty with orgasm	Prevalence: 20.4%<br>By treatment:<br>Srg, C: 21.3%<br>Srg, C + RT: 20.8%<br>Srg, M: 16.8%	Prevalence:14.8%	PR=1.38 * †<br>By treatment:<br>PR= 1.44 * †<br>PR= 1.41 * †<br>PR= 1.14 †	95%CI: 1.10-1.73<br>By treatment:<br>95%CI: 1.08-1.92<br>95%CI: 1.08-1.83<br>95%CI: 0.72-1.78	
Broeckel et al., 2002 [40]<br><br>United States	Convenience sample<br><br>58 breast cancer survivors who had a spouse or partner, free of recurrence for >5 years, with no known neurological disorder, and no history of other cancer.	I (26%)<br>II (62%)<br>III (10%)<br>Unknown (2%)	Srg, C: 50%<br>Srg, M: 47%<br>CT: 100%<br>RT: 71%<br>HT: 48%	7.7 (2.3), 5.2-15.2	Convenience sample<br><br>61 women with no history of cancer who had a spouse or partner, recruited among the friends of the women who had breast cancer; individual matching for age (± 6 years).	Scale: MOS-SFS<br>Overall	Mean score (SD):<br>1.95 (1.05)	Mean score (SD):<br>1.50 (0.70)	-	P≤0.01 *	Sexual dysfunction positively correlated with vaginal dryness in breast cancer survivors.
						Interest	2.06 (1.16)	1.67 (0.83)	-	P≤0.05 *	
						Enjoyment	1.72 (0.94)	1.38 (0.74)	-	P≤0.01 *	
						Arousal	1.87 (1.08)	1.40 (0.83)	-	P≤0.01 *	
						Orgasm	1.78 (1.01)	1.44 (0.80)	-	P≤0.05 *	
Rubino et al., 2007 [10]<br><br>Italy	Convenience sample<br><br>33 consecutive patients who had had breast-reconstruction after mastectomy, in 2001-2002, in one hospital.	ND	Srg, M: 100%<br>Srg, R: 100%	ND (ND), >1	Convenience sample<br><br>33 healthy women, randomly selected amongst the personnel of the local university.	Psychiatric interview	Prevalence: 18.5%	Prevalence: 9.1%	PR=2.03 †	95%CI: 0.19-21.26	-

Vazquez-Ortiz et al., 2010 [104]<br><br>Spain	Convenience sample<br><br>30 women aged 25-59 years who had mastectomy ≥1 year ago, were free of disease, in a stable heterosexual relationship, able to read and write and with no psychological or psychiatric treatment in the last 10 years, recruited from hospitals.	I (13.3%)<br>II (60.0%)<br>III-A (26.7%)	Srg, M: 100%	ND (ND), 2-5	Convenience sample<br><br>30 women without breast cancer aged 25-59, assistants to talks and workshops about woman's health, who did not have an incapacitating or severe disease.	Scale: SAI-E Arousal	Mean score (SD): 68.5 (23.9)	Mean score (SD): 72.6 (23.7)	-	P=0.690	-
						Scale: SAI-E Satisfaction	Mean score (SD): 72.3 (23.3)	Mean score (SD): 76.9 (23.9)	-	P=0.524	
						Scale: WSQ<br>Sex frequency per month: 0	10.0%	3.3%	PR=3.33 †	95%CI: 0.33-33.27	-
						1-3	20.0%	13.3%	PR=1.50 †	95%CI: 0.47-4.80	
						4-6	33.3%	30.0%	PR=1.11 †	95%CI: 0.53-2.34	
						7-9	13.3%	20.0%	PR=0.67 †	95%CI: 0.21-2.12	
						>9	23.3%	33.3%	PR=0.70 †	95%CI: 0.31-1.59	
						Orgasm frequency during sex<br>Never (0%)	7.1%	3.3%	PR=2.15 †	95%CI: 0.21-22.11	-
						Sometimes (1-29%)	14.3%	10.0%	PR=1.43 †	95%CI: 0.36-5.72	
						Often (30-58%)	17.9%	23.3%	PR=0.77 †	95%CI: 0.28-2.10	
						Most of the time (60-89%)	21.4%	16.7%	PR=1.28 †	95%CI: 0.45-3.67	
						Almost always (90-100%)	39.3%	46.7%	PR=0.84 †	95%CI: 0.47-1.51	

BC = breast cancer; CT = chemotherapy; FSFI = Female Sexual Functioning Index [105];
HT = hormone therapy; MOS-SFS = MOS Sexual Functioning Scale [106]; ND = not defined; PR = prevalence ratio; RT = radiotherapy; SAI-E = Sexual Arousal and Satisfaction Scale - Expanded [107]; SD = standard deviation; Srg, C = Breast conserving surgery; Srg, M = Mastectomy; Srg, R = Breast reconstructive surgery; WSQ = Women's Sexuality Questionnaire [108].
* There was some statistical evidence (P<0.05) for a different prevalence, risk or severity of anxiety between breast cancer survivors and women who did not have cancer.
† Prevalence ratio calculated by the authors of the present study.

Supplementary Table 7. Other outcomes: characteristics and results of the studies that provided data on the frequency and/or severity of bipolar disorders, obsessive-compulsive problems, post-traumatic stress, sleep-wake disturbances, somatization and suicide in breast cancer survivors (>1 year) and women who did not have cancer.

First author, year of publication<br><br>Country	Breast cancer survivors: Type of population and main characteristics	Breast cancer survivors: Stage at diagnosis (%)	Breast cancer survivors: Breast cancer treatments (%)	Breast cancer survivors: Time since diagnosis/ treatment in years: mean/ median (SD), range	Comparison group: Type of population and main characteristics	Outcome assessment	Prevalence / cumulative incidence of the outcome: Breast cancer survivors	Prevalence / cumulative incidence of the outcome: Comparison group	Relative risk estimate (RR, OR, SIR, PR)	P-value or 95% confidence interval	Notes
**Bipolar disorder**											
Hung et al., 2013 [2]<br><br>Taiwan	Population-based<br><br>26,629 women with no prior mood disorder and cancer, with primary breast cancer registered in the National Health Insurance Database in 2000-2005.	All	ND	2.7 (ND), ND-7<br><br>(median follow up years for breast cancer survivors: 2.7; for matched cohort: 3.21)	Population-based<br><br>26,629 women randomly selected from 1 million women with no history of breast cancer in the same database; individual matching for age and Charlson comorbidity score (categories of matching not reported).	EHR, recorded in the Registry for Catastrophic Illness with an ICD-9-CM code for anxiety (ICD-9-CM codes: 296.0X-296.1X, 296.4X-296.8X)	Cumulative incidence:<br>0.3%	Cumulative incidence:<br>0.1%	RR=2.06 *	95%CI: 1.37-3.15	Approximate cumulative incidence values estimated from the graphics provided in the original study.<br><br>P value for the log-rank test comparing the Kaplan-Meier curves: P<0.001
				2			0.3%	0.1%	RR=3.0 * †	95%CI: 2.56-3.39	
				4			0.4%	0.2%	RR=2.0 * †	95%CI: 1.82-2.19	
				6			0.6%	0.3%	RR=2.0 * †	95%CI: 1.86-2.16	
**Obsessive-compulsive problems**											
Amir et al., 2002 [13]<br><br>Israel	Convenience sample<br><br>39 women free of cancer symptoms for ≥3 years and not under active treatment, identified in 2 hospitals.	I (46%)<br>II (46%)<br>III (8%)	Srg, C: 20%<br>Srg, M: 80%<br>CT: 66%<br>RT: 41%<br>HT: 46%	6.5 (ND), ≥5	Convenience sample<br><br>39 women without any life-threatening disease, recruited by unknown methods; matched for age and education; matched for age and education (method of matching not reported).	Scale: SCL-90	SCL-90 mean score (SD):<br><br>0.92 (0.70)	SCL-90 mean score (SD):<br><br>0.68 (0.42)	-	P<0.001 *	Higher SCL-90 scores indicate more obsessive-compulsive symptoms.<br><br>Women who had breast cancer and PTSD symptoms had more obsessive-compulsive problems than those who did not have PTSD symptoms (P<0.01).
**Post-traumatic stress**											
Gurevich et al., 2004 [109]<br><br>Canada	Convenience sample<br><br>66 women with a good working knowledge of English ≥1 year post breast cancer treatments with negative mammography before.	Local (61%)<br>Regional (30.5%)<br>Distant (2%)<br>Unknown (11%)	Srg: 96.6%<br>CT: 48%<br>RT: 71%<br>HT: 48%	6.6 (4.5), ≥1	Convenience sample<br><br>69 'healthy' women undergoing surveillance mammography in the same hospital.	Scale: SASRQ<br>Dissociative<br>Re-experiencing<br>Avoidance<br>Arousal<br>Impairment<br>Total acute stress	SASRQ mean scores (SD):<br>1.07 (1.05)<br>1.23 (1.25)<br>1.34 (1.21)<br>1.96 (1.40)<br>1.29 (1.30)<br>1.37 (1.05)	SASRQ mean scores (SD):<br>0.45 (0.80)<br>0.58 (0.95)<br>0.83 (1.17)<br>1.00 (1.21)<br>0.66 (1.10)<br>0.69 (0.91)	-	P<0.0001 *<br>P<0.001 *<br>P<0.02 *<br>P<0.0001 *<br>P<0.003 *<br>P<0.0001 *	-

Voigt et al., 2016 [110]<br><br>Germany	Convenience sample<br>150 women aged 18-65 years, newly diagnosed with breast cancer at recruitment, with no history of psychotic disorders	0 (7%)<br>I (42%)<br>II (41.4%)<br>IIIc (%9.6)	Srg, M: 26%<br>Srg, C: 74%<br>CT: 58%	~ 1	Convenience sample<br>56 women aged 18-65 years, who never had cancer, who attended the same institution as cases for breast imagining and did not require further tests	SCID, number of PTSD symptoms	Prevalence of PTSD related to BC: 2.0%<br><br>Prevalence of PTSD related to stressors other than BC: 0.7%	Prevalence of PTSD related to stressors other than BC: 0%	PR= 1.51 †	95%CI: 0.17-13.20	Mean number of PTSD symptoms (SD) in breast cancer survivors: 1.7 (2.3); significantly different from the mean number of symptoms in controls (P<0.001).
Yang et al., 2017 [4]<br><br>Sweden	Population based<br><br>All 40,849 women diagnosed with an invasive breast cancer at the age of 20-80 years between 2001-2009	I-IV	ND	4.5 (4.5), 0-10<br><br>(median (IQR) duration of follow up: 4.4 (4.5))	Population based<br><br>452,507 women randomly selected from the respondents to the 1990 census	EHR, ICD-10 diagnostic codes for stress-related disorders (F430-2, F438-9) at in patient or outpatient hospital visits	Cumulative incidence:<br>0.9%	Cumulative incidence:<br>0.5%	SIR= 1.77 *<br><br>By age group:<br>20-44: SIR= 1.68 *<br>45-54: SIR= 1.78 *<br>55-64: SIR= 1.89 *<br>65-80: SIR= 1.64 *	95%CI: 1.60-1.95<br><br>By age group:<br>95%CI: 1.36-2.08<br>95%CI: 1.52-2.09<br>95%CI: 1.56-2.28<br>95%CI: 1.23-2.19	Standardised incidence ratios were standardised by calendar period (1-year categories), age (5-year categories), and region of residence (North, Stockholm-Gotland, South, Southeast, Uppsala-Orebro, West).
				0-0.5			-	-	SIR= 4.22 *	95%CI: 3.44-5.19	
				0.5-1			-	-	SIR= 2.73 *	95%CI: 2.11-3.52	
				1-2			-	-	SIR= 1.72 *	95%CI: 1.36-2.17	
				2-5			-	-	SIR= 1.36 *	95%CI: 1.14-1.63	
				5-10			-	-	SIR= 0.98	95%CI: 0.73-1.32	
	Population based<br><br>All 40,849 women diagnosed with an invasive breast cancer at the age of 20-80 years between 2001-2009	0	ND	4.5 (4.5), 0-10<br><br>(median (IQR) duration of follow up: 4.4 (4.5))	Population based<br><br>452,507 women randomly selected from the respondents to the 1990 census	EHR, ICD-10 diagnostic codes for stress-related disorders (F430-2, F438-9) at in patient or outpatient hospital visits	Cumulative incidence:<br>0.6%	Cumulative incidence:<br>0.5%	SIR= 1.02<br><br>By age group:<br>20-44: SIR= 0.38<br>45-54: SIR= 1.06<br>55-64: SIR= 1.46<br>65-80: SIR= 1.15	95%CI: 0.70-1.50<br><br>By age group:<br>95%CI: 0.09-1.51<br>95%CI: 0.60-1.87<br>95%CI: 0.76-2.81<br>95%CI: 0.37-3.56	
				0-0.5			-	-	SIR= 2.76 *	95%CI: 1.31-5.79	
				0.5-1			-	-	SIR= 0.78	95%CI: 0.20-3.14	
				1-2			-	-	SIR= 1.04	95%CI: 0.43-2.51	
				2-5			-	-	SIR= 0.88	95%CI: 0.46-1.69	
				5-10			-	-	SIR= 0.57	95%CI: 0.18-1.76	
Sleep-wake disturbances											
Ancoli-Israel et al., 2014 [33]<br><br>United States	Convenience sample<br>44 women who had been newly diagnosed with breast cancer 1 year before, and scheduled to receive ≥4 cycles of CT, with no psychological impairments and not receiving RT at recruitment.	I (27.9%)<br>II (39.7%)<br>III (30.9%)<br>Unknown (1.5%)	Srg, C: 45.6%<br>Srg, M: 49.7%<br>CT: 100%	~ 1<br><br>(follow up at 1 year after CT)	Convenience sample<br>35 cancer-free friends of the women who had breast cancer, or 'volunteers', with no psychological impairments at the time of recruitment individual matching for age (±5 years), ethnicity and education (categories of ethnicity and education not reported).	Nocturnal total sleep time	Mean time (SD), hours:<br>7.01 (0.74)	Mean time (SD), hours:<br>7.07 (0.66)	-	P>0.05	Sleep measure by wrist activity, using an actigraph during 72 consecutive hours.
						Daytime total nap time	Mean time (SD), hours:<br>0.49 (0.47)	Mean time (SD), hours:<br>0.36 (0.44)		P=0.63	
						Scale: PSQI	PSQI mean scores (SD):<br>7.4 (ND)	PSQI mean scores (SD):<br>5.0 (ND)	-	P=0.02 *	

El Rafihi-Ferreira et al., 2011 [111]<br><br>Brazil	Convenience sample<br><br>50 women with a previous diagnosis of breast cancer without encephalopathies or severe psychiatric disorders. Patients were all disease free at enrolment.	I-II (100%)	Srg, ND: 40%<br>CT: 66%<br>RT: 54%<br>HT: 77%	3.8 (2.8), 1-10	Convenience sample<br><br>50 women without a previous cancer diagnosis, encephalopathies or severe psychiatric disorders.	Scale: PSQI	Prevalence:40%	Prevalence: 50%	PR=0.8 †	95%CI: 0.52-1.24	Cut-off for case: score >5
						Cannot get to sleep in 30 min	Prevalence: 42%	Prevalence: 38%	PR= 1.1 †	95%CI: 0.68-1.79	
						Wake up in the middle of the night or early morning	40%	22%	PR= 1.82 †	95%CI: 0.98-3.39	Cut-off for case: reported problems three or more times a week.<br><br>Worse sleep quality associated with poorer quality of life for the social domain, and domains of physical and psychological health (P<0.05).<br><br>Women who had had breast cancer and had worse quality of sleep reported higher depressive symptomatology compared to those with good quality of sleep (SDS mean scores 20.8 (7.12) vs. 16.6 (3.76), P<0.05).
						Get up to use the bathroom	52%	26%	PR= 2.0 * †	95%CI: 1.17-3.43	
						Cannot breathe comfortably	8%	8%	PR= 1.0 †	-	
						Cough or snore loudly	16%	16%	PR= 1.0 †	-	
						Feel too cold	4%	6%	PR= 0.67 †	95%CI: 0.12-3.82	
						Feel too hot	36%	14%	PR= 2.57 * †	95%CI: 1.18-5.61	
						Pain	14%	20%	PR= 0.70 †	95%CI: 0.29-1.69	
						Sleep medication	12%	16%	PR= 0.75 †	95%CI: 0.28-2.00	
						Daytime sleepiness	2%	4%	PR= 0.50 †	95%CI: 0.05-5.34	
						<6h of sleep	18%	14%	PR= 1.29 †	95%CI: 0.52-3.18	
Otte et al., 2010 [43]<br><br>United States	Convenience sample<br><br>246 breast cancer survivors free of cancer at recruitment, with no history of other cancers and able to speak, read and write English	I (ND)<br>II (ND)<br>III (ND)	Srg, C: 42%<br>Srg, M: 59%<br>CT: 89%<br>RT: ND<br>HT: 33%	5.6 (2.0), 2-10	Convenience sample<br><br>246 women in general good health with no history of breast cancer recruited by acquaintance referral, self-referral or from corporative group; individual matching for age (±5 years).	Scale: PSQI Overall score	PSQI mean scores (SD):<br>7.31 (3.80)	PSQI mean scores (SD):<br>5.80 (3.45)	-	P<0.01 *	Adjusted for race (minority vs. not minority) and menopausal status (pre or post menopausal).<br><br>Determinants sleep-wake disorders in women who had breast cancer: race other than Caucasian, having hot flashes, poor physical functioning and depression.
						Sleep quality	1.20 (ND)	0.85 (ND)	-	P<0.01 *	
						Sleep latency	1.39 (ND)	1.00 (ND)	-	P<0.01 *	
						Sleep disturbance	1.50 (ND)	1.31 (ND)	-	P<0.01 *	
						Sleep medication	0.65 (ND)	0.61 (ND)	-	P=0.70	
						Sleep efficiency	0.59 (ND)	0.57 (ND)	-	P=0.77	
						Sleep duration	0.98 (ND)	0.84 (ND)	-	P=0.03 *	Adjusted for race.
						Daytime dysfunction	0.96 (ND)	0.70 (ND)	-	P<0.01 *	-
Dahl et al., 2011 [8]<br><br>Norway	Convenience sample<br><br>337 tumor free breast cancer survivors treated with radiotherapy during 1998 and 2002 in one hospital.	II (ND)<br>III (ND)	Srg, C: 24%<br>Srg, M: 76%<br>CT: 82%<br>RT: 100%<br>HT: 81%	3.9 (ND), 2.6-6.9	Convenience sample<br>1,685 women randomly selected from a population-based sample of women with no history of cancer whose questionnaires had complete data; matched individual matching for age (± 5 years).	Prevalence of regular use of hypnotics	Prevalence:<br>15%	Prevalence:<br>4%	PR=3.75 * †	95%CI: 2.65-5.30	Adjusted for level of education, on disability pension and menopausal status.

Von Ah et al., 2012 [45]<br><br>United States	Convenience sample<br><br>62 non-Hispanic African American women diagnosed with non-metastatic breast cancer and able to read and write English, recruited by medical record review and by self-referral.	I-IIB (85.7%)<br>IIIB (14.3%)	Srg, C: 0%<br>Srg, M: 60.3%<br>CT & RT: 54.6%<br>HT: ND	5.0 (2.7), 2-10	Convenience sample<br><br>78 African American women with no history of breast cancer, recruited through community advertisements and events.	Scale: PSQI	PSQI mean scores (SD):<br>9.0 (4.2)	PSQI mean scores (SD):<br>6.1 (4.0)	-	P=<0.001 *	Mean scores adjusted for age, income, years of education and body mass index.
Somatization											
Cohen et al., 2011 [5]<br><br>Israel	Convenience sample<br><br>56 married Israeli Arab breast cancer survivors, post treatment and free of disease recruited from one hospital.	I-III (ND%)	Srg, C: 48.2%<br>Srg, M: 51.8%<br>Srg, R: 12.5%<br>CT: 85.7%<br>RT: 85.7%<br>HT: 58.9%	4.8 (4.2), 1-17	Convenience sample<br><br>66 married and 'healthy' Arab women living in northern Israel, approached in community settings; individual matching for age and education (matching categories not reported).	Scale: BSI-18	BSI-18 mean score (SD):<br><br>2.6 (1.2)	BSI-18 mean score (SD):<br><br>1.8 (0.8)	-	P<0.001 *	More somatic symptoms in breast cancer survivors were associated with lower education, religiosity, depression, anxiety, emotional distress and lower body image (P<0.05).
Amir et al., 2002 [13]<br><br>Israel	Convenience sample<br><br>39 women free of cancer symptoms for ≥3 years and not under active treatment, identified through two hospitals.	I (46%)<br>II (46%)<br>III (8%)	Srg, C: 20%<br>Srg, M: 80%<br>CT: 66%<br>RT: 41%<br>HT: 46%	6.5 (ND), ≥5	Convenience sample<br><br>39 women who did not experience any life-threatening disease, recruited by unknown methods; matched for age and education (method of matching not reported).	SCL-90	SCL-90 mean score:<br><br>0.92 (0.86)	SCL-90 mean score:<br><br>0.51 (0.47)	-	P<0.001 *	Higher SCL-90 scores indicate more somatic symptoms.<br><br>Women who had breast cancer and reported PTSD symptoms had more somatic symptoms than women who did not have PTSD symptoms: 1.61 (1.06) vs. 0.77 (0.60), P<0.01.
Suicide											
Schairer et al., 2006 [112]<br><br>Denmark, Finland, Norway, Sweden, United States<br><br>(continues)	Population based<br><br>723,810 one-year breast cancer survivors diagnosed between 1953 and 2001.	All	ND	8.7 (ND), 1-49<br><br>(mean follow up duration: 7.7 years, range <1 month to 49 years)	Population-based<br><br>General female population in each of the countries	Official mortality databases in each country. ICD-7 codes: E963 and E970 - 979; ICD-8 and ICD-9: E950 - E959; and ICD-10: X60 - X84.	Incidence rate: 1.5 per 10,000 person-years<br><br>Cumulative incidence of suicide by time since diagnosis:<br>5 yrs: 0.05%<br>10 yrs: 0.10%<br>20 yrs: 0.16%<br>30 yrs: 0.20%	Incidence rate: 1.09 per 10,000 person-years	SIR= 1.37 *	95%CI: 1.28-1.47	-
									By country<br>US: SIR= 1.49 *<br>Sweden: SIR= 1.27 *<br>Denmark: SIR= 1.25*<br>Finland: SIR= 1.53 *<br>Norway: SIR= 1.40 *	By country<br>95%CI: 1.32-1.70<br>95%CI: 1.12-1.45<br>95%CI: 1.07-1.46<br>95%CI: 1.28-1.83<br>95%CI: 1.07-1.81	-
									By calendar period<br>1953-59: SIR=1.86*<br>1960-69: SIR=1.72*<br>1970-79: SIR=1.31*<br>1980-89: SIR=1.29*<br>1990-2001: SIR=1.36*	By calendar period<br>95%CI: 1.20-2.78<br>95%CI: 1.42-2.07<br>95%CI: 1.15-1.49<br>95%CI: 1.15-1.46<br>95%CI: 1.18-1.57	-

Schairer et al., 2006 [112]<br><br>Denmark, Finland, Norway, Sweden, United States<br><br>(continued)	Population based<br><br>723,810 one-year breast cancer survivors diagnosed between 1953 and 2001.	All	ND	8.7 (ND), 1-49	Population-based<br><br>General female population in each of the countries	Official mortality databases in each country. ICD-7 codes: E963 and E970 - 979; ICD-8 and ICD-9: E950 - E959; and ICD-10: X60 - X84.	Incidence rate: 1.5 per 10,000 person-years<br><br>Cumulative incidence of suicide by time since diagnosis:<br>5 yrs: 0.05%<br>10 yrs: 0.10%<br>20 yrs: 0.16%<br>30 yrs: 0.20%	Incidence rate: 1.09 per 10,000 person-years	By race<br>White: SIR=1.36 *<br>Black: SIR=2.88 *<br>Other: SIR=1.02	By race<br>95%CI: 1.27-1.46<br>95%CI: 1.44-5.17<br>95%CI: 0.44-2.01	-
									By age<br><40: SIR=1.34 *<br>40-49: SIR=1.42 *<br>50-59: SIR=1.50 *<br>60-69: SIR=1.26 *<br>≥70: SIR=1.24 *	By age<br>95%CI: 1.24-1.62<br>95%CI: 1.32-1.71<br>95%CI: 1.09-1.47<br>95%CI: 1.04-1.48<br>95%CI: 1.24-1.62	-
									By time since diagnosis, years<br>1: SIR=1.51 *<br>2: SIR=1.49 *<br>3: SIR=1.57 *<br>4: SIR=1.31 *<br>5-9: SIR=1.30 *<br>10-14: SIR=1.28 *<br>15-19: SIR=1.25<br>20-24: SIR=1.32<br>≥25: SIR=1.35	By time since diagnosis, years<br>95%CI: 1.25-1.82<br>95%CI: 1.22-1.82<br>95%CI: 1.27-1.93<br>95%CI: 1.02-1.66<br>95%CI: 1.14-1.49<br>95%CI: 1.07-1.54<br>95%CI: 0.95-1.62<br>95%CI: 0.89-1.90<br>95%CI: 0.82-2.12	
									By stage at diagnosis<br>Local: SIR=1.38 *<br>Regional: SIR=1.55*<br>Distant: SIR=2.11 *<br>Unknown: SIR=1.05*	By stage at diagnosis<br>95%CI: 1.24-1.53<br>95%CI: 1.34-1.79<br>95%CI: 1.16-3.55<br>95%CI: 0.73-1.50	Includes only patients from the US, Demark, Finland and Norway.
									By treatment<br>Surgery only<br>SIR= 1.40 *<br>Radiotherapy, no chemotherapy<br>SIR= 1.46 *<br>Chemotherapy, no radiotherapy<br>SIR= 1.12<br>Radiotherapy and chemotherapy<br>SIR= 1.50 *<br>Other/none/unknown<br>SIR= 1.84 *	By treatment<br><br>95%CI: 1.24-1.58<br><br>95%CI: 1.27-1.67<br><br>95%CI: 0.80-1.55<br><br>95%CI: 1.09-2.02<br><br>95%CI: 1.14-2.96	Refers to initial course of treatment only;<br><br>Includes only patients from the US, Demark, Finland and Norway.
									Breast conserving surgery<br>SIR= 1.22<br>Radical mastectomy<br>SIR= 1.30 *	 95%CI: 0.89-1.64<br><br>95%CI: 1.04-1.63	US women only, 1983-2001.

Fang et al., 2012 [113]	Population based<br><br>74,977 women diagnosed with primary breast cancer between 1991 and 2006	All	ND	>1<br><br>(Mean follow up time of the all cancer cohorts was 4.07 years (median 2.65, range 0 to 15.99)	Population based<br><br>Women not diagnosed with cancer during follow up.	ICD-9 codes E950–E959 and ICD-10 codes X60–X84 and Y870	-	-	RR= 1.6 *	95%CI: 1.2-2.1	RR adjusted for age at follow-up (≤49 years, 5-yr groups for 50 to 74 yrs, ≥75 yrs), calendar period at follow-up (5-year groups), civil status (cohabitation or non-cohabitation), socioeconomic status (blue-collar, white-collar, self-employed, or unclassified), and education (≥9 years, <9 years, or missing).

BC = breast cancer; BSI-18 = Brief Symptom Inventory-18 [22]; CT = chemotherapy; EHR = electronic health records; HT = hormone therapy; ICD-9-CM = The International Classification of Diseases, Ninth Revision, Clinical Modification; IRR = incidence rate ratio; ND = not defined; PR = prevalence ratio; PSQI = Pittsburgh Sleep Quality Index [114]; RR = relative risk; RT = radiotherapy; SASRQ = Stanford Acute Stress Reaction Questionnaire [115]; SCL-90 = Somatization subscale of Symptoms Checklist-90 [27]; SD = standard deviation; Srg, C = Breast conserving surgery; Srg, M = Mastectomy.

* There was some statistical evidence (P<0.05) for a different prevalence, risk or severity of anxiety between breast cancer survivors and women who did not have cancer.

† Prevalence ratio calculated by the authors of the present study.

References

1. Hjerl K, Andersen EW, Keiding N*, et al.* Increased incidence of affective disorders, anxiety disorders, and non-natural mortality in women after breast cancer diagnosis: a nation-wide cohort study in Denmark. Acta Psychiatr Scand 2002;105(4):258-64.

2. Hung YP, Liu CJ, Tsai CF*, et al.* Incidence and risk of mood disorders in patients with breast cancers in Taiwan: a nationwide population-based study. Psychooncology 2013;22(10):2227-34.

3. Khan NF, Ward AM, Watson E*, et al.* Consulting and prescribing behaviour for anxiety and depression in long-term survivors of cancer in the UK. Eur J Cancer 2010;46(18):3339-44.

4. Yang H, Brand JS, Fang F*, et al.* Time-dependent risk of depression, anxiety, and stress-related disorders in patients with invasive and in situ breast cancer. Int J Cancer 2017;140(4):841-852.

5. Cohen M, Mabjish AA, Zidan J. Comparison of Arab breast cancer survivors and healthy controls for spousal relationship, body image, and emotional distress. Qual Life Res 2011;20(2):191-8.

6. Boehmer U, Ozonoff A, Potter J. Sexual Minority Women's Health Behaviors and Outcomes After Breast Cancer. LGBT Health 2015;2(3):221-7.

7. Calvio L, Peugeot M, Bruns GL*, et al.* Measures of cognitive function and work in occupationally active breast cancer survivors. J Occup Environ Med 2010;52(2):219-27.

8. Dahl AA, Nesvold IL, Reinertsen KV*, et al.* Arm/shoulder problems and insomnia symptoms in breast cancer survivors: cross-sectional, controlled and longitudinal observations. Sleep Med 2011;12(6):584-90.

9. Miao H, Li J, Hu S*, et al.* Long-term cognitive impairment of breast cancer patients after chemotherapy: A functional MRI study. Eur J Radiol 2016;85(6):1053-7.

10. Rubino C, Figus A, Lorettu L*, et al.* Post-mastectomy reconstruction: a comparative analysis on psychosocial and psychopathological outcomes. J Plast Reconstr Aesthet Surg 2007;60(5):509-18.

11. Boele FW, Schilder CM, de Roode ML*, et al.* Cognitive functioning during long-term tamoxifen treatment in postmenopausal women with breast cancer. Menopause 2015;22(1):17-25.

12. Kreukels BP, van Dam FS, Ridderinkhof KR*, et al.* Persistent neurocognitive problems after adjuvant chemotherapy for breast cancer. Clin Breast Cancer 2008;8(1):80-7.

13. Amir M, Ramati A. Post-traumatic symptoms, emotional distress and quality of life in long-term survivors of breast cancer: a preliminary research. J Anxiety Disord 2002;16(2):195-206.

14. Garcia-Torres F, Alos FJ. Identification of different depressive symptoms after mastectomy. Psychooncology 2013;22(12):2857-9.

15. Castellon SA, Ganz PA, Bower JE*, et al.* Neurocognitive performance in breast cancer survivors exposed to adjuvant chemotherapy and tamoxifen. J Clin Exp Neuropsychol 2004;26(7):955-69.

16. Weitzner MA, Meyers CA, Stuebing KK, *et al.* Relationship between quality of life and mood in long-term survivors of breast cancer treated with mastectomy. Support Care Cancer 1997;5(3):241-8.

17. Root JC, Andreotti C, Tsu L, *et al.* Learning and memory performance in breast cancer survivors 2 to 6 years post-treatment: the role of encoding versus forgetting. J Cancer Surviv 2016;10(3):593-9.

18. Conroy SK, McDonald BC, Smith DJ, *et al.* Alterations in brain structure and function in breast cancer survivors: effect of post-chemotherapy interval and relation to oxidative DNA damage. Breast Cancer Res Treat 2013;137(2):493-502.

19. McDonald BC, Conroy SK, Ahles TA, *et al.* Gray matter reduction associated with systemic chemotherapy for breast cancer: a prospective MRI study. Breast Cancer Res Treat 2010;123(3):819-28.

20. Klein D, Mercier M, Abeilard E, *et al.* Long-term quality of life after breast cancer: a French registry-based controlled study. Breast Cancer Res Treat 2011;129(1):125-34.

21. Saleeba AK, Weitzner MA, Meyers CA. Subclinical psychological distress in long-term survivors of breast cancer: a preliminary communication. Journal of Psychological Oncology 1996;14(1):83-93.

22. Derogatis L. *The Brief Symptom Inventory (BSI) 18: Administration, scoring, and procedures manual*. Minneapolis, MN: NCS Pearson; 2000.

23. Zigmond AS, Snaith RP. The hospital anxiety and depression scale. Acta Psychiatr Scand 1983;67(6):361-70.

24. Hamilton M. The assessment of anxiety states by rating. Br J Med Psychol 1959;32(1):50-5.

25. Frojdh K, Hakansson A, Karlsson I. The Hopkins Symptom Checklist-25 is a sensitive case-finder of clinically important depressive states in elderly people in primary care. Int J Geriatr Psychiatry 2004;19(4):386-90.

26. Miguel-Tobal JJ, Cano-Vindel AR. *Inventario de Situaciones y Respuestas de Ansiedad. Manual*. Madrid: TEA Ediciones; 1988.

27. Derogatis LR. *The SCL-90 manual F: scoring, administration and procedures for the SCL-90*. Baltimore, MD: School of Medicine, Clinical Psychometrics Unit, Johns Hopkins University; 1977.

28. Spielberger C, Gorsuch R, Lushene R. *State-trait anxiety inventory manual*. Palo Alto: Consulting Psychologist Press; 1970.

29. Suppli NP, Johansen C, Christensen J, *et al.* Increased risk for depression after breast cancer: a nationwide population-based cohort study of associated factors in Denmark, 1998-2011. J Clin Oncol 2014;32(34):3831-9.

30. Earle CC, Neville BA, Fletcher R. Mental health service utilization among long-term cancer survivors. J Cancer Surviv 2007;1(2):156-60.

31. Kim MS, Kim SY, Kim JH, *et al.* Depression in breast cancer patients who have undergone mastectomy: A national cohort study. PLoS One 2017;12(4):e0175395.

32. Aerts L, Christiaens MR, Enzlin P, *et al.* Sexual functioning in women after mastectomy versus breast conserving therapy for early-stage breast cancer: a prospective controlled study. Breast 2014;23(5):629-36.

33. Ancoli-Israel S, Liu L, Rissling M*, et al.* Sleep, fatigue, depression, and circadian activity rhythms in women with breast cancer before and after treatment: a 1-year longitudinal study. Support Care Cancer 2014;22(9):2535-45.

34. Kesler S, Janelsins M, Koovakkattu D*, et al.* Reduced hippocampal volume and verbal memory performance associated with interleukin-6 and tumor necrosis factor-alpha levels in chemotherapy-treated breast cancer survivors. Brain Behav Immun 2013;30 Suppl:S109-16.

35. Bailey EH, Perez M, Aft RL*, et al.* Impact of multiple caregiving roles on elevated depressed mood in early-stage breast cancer patients and same-age controls. Breast Cancer Res Treat 2010;121(3):709-18.

36. Hermelink K, Buhner M, Sckopke P*, et al.* Chemotherapy and Post-traumatic Stress in the Causation of Cognitive Dysfunction in Breast Cancer Patients. J Natl Cancer Inst 2017;109(10).

37. Lee MK, Park S, Lee ES*, et al.* Social support and depressive mood 1 year after diagnosis of breast cancer compared with the general female population: a prospective cohort study. Support Care Cancer 2011;19(9):1379-92.

38. Bizetti Pelai E, Ibde Jaquiel Figueira J, Madia Mantovani A*, et al.* Quality of life, depression and pain in women after breast cancer surgery. Ter Man 2012;10(48):161-167.

39. Nguyen CM, Yamada TH, Beglinger LJ*, et al.* Cognitive features 10 or more years after successful breast cancer survival: comparisons across types of cancer interventions. Psychooncology 2013;22(4):862-8.

40. Broeckel JA, Thors CL, Jacobsen PB*, et al.* Sexual functioning in long-term breast cancer survivors treated with adjuvant chemotherapy. Breast Cancer Res Treat 2002;75(3):241-8.

41. Claus EB, Petruzella S, Carter D*, et al.* Quality of life for women diagnosed with breast carcinoma in situ. J Clin Oncol 2006;24(30):4875-81.

42. Koppelmans V, de Ruiter MB, van der Lijn F*, et al.* Global and focal brain volume in long-term breast cancer survivors exposed to adjuvant chemotherapy. Breast Cancer Res Treat 2012;132(3):1099-106.

43. Otte JL, Carpenter JS, Russell KM*, et al.* Prevalence, severity, and correlates of sleep-wake disturbances in long-term breast cancer survivors. J Pain Symptom Manage 2010;39(3):535-47.

44. Von Ah D, Harvison KW, Monahan PO*, et al.* Cognitive function in breast cancer survivors compared to healthy age- and education-matched women. Clin Neuropsychol 2009;23(4):661-74.

45. Von Ah DM, Russell KM, Carpenter J*, et al.* Health-related quality of life of african american breast cancer survivors compared with healthy African American women. Cancer Nurs 2012;35(5):337-46.

46. Frazzetto P, Vacante M, Malaguarnera M*, et al.* Depression in older breast cancer survivors. BMC Surg 2012;12 Suppl 1:S14.

47. Min SY, Kim HY, Jung SY*, et al.* Oncological safety and quality of life associated with mastectomy and immediate breast reconstruction with a latissimus dorsi myocutaneous flap. Breast J 2010;16(4):356-61.

48. Beck AT, Ward CH, Mendelson M*, et al.* An inventory for measuring depression. Arch Gen Psychiatry 1961;4:561-71.

49. Beck AT, Steer RA, Brown G. *Beck Depression Inventory–II: Manual*. San Antonio, Tex: Psychological Corp; 1996.

50. Aghakhani A, Chan EK. Test Reviews: Bracken, B. A., & Howell, K. (2004). Clinical Assessment of Depression. Odessa, FL: Psychological Assessment Resources. Journal of Psychoeducational Assessment 2007;25(4):416-422.

51. Radloff LS. The CES-D scale: A self-report depression scale for research in the general population. Applied Psychol Measurement 1977;1:385–401.

52. Yesavage JA, Brink TL, Rose TL*, et al.* Development and validation of a geriatric depression screening scale: a preliminary report. J Psychiatr Res 1982;17(1):37-49.

53. Hamilton M. Development of a rating scale for primary depressive illness. Br J Soc Clin Psychol 1967;6(4):278-96.

54. Gräfe K, Zipfel S, Herzog W*, et al.* Screening psychischer Störungen mit dem "Gesundheitsfragebogen für Patienten (PHQ-D). Ergebnisse der deutschen Validierungsstudie. Diagnostica 2004;50(4):171-181.

55. Shin H, Kim C, Park Y*, et al.* Validity of Zung's self-rating depression scale: detection of depression in primary care. J Korean Acad Fam Med 2000;21:1317-30.

56. Ahles TA, Saykin AJ, McDonald BC*, et al.* Longitudinal assessment of cognitive changes associated with adjuvant treatment for breast cancer: impact of age and cognitive reserve. J Clin Oncol 2010;28(29):4434-40.

57. Collins B, Mackenzie J, Tasca GA*, et al.* Persistent cognitive changes in breast cancer patients 1 year following completion of chemotherapy. J Int Neuropsychol Soc 2014;20(4):370-9.

58. Fan HG, Houede-Tchen N, Yi QL*, et al.* Fatigue, menopausal symptoms, and cognitive function in women after adjuvant chemotherapy for breast cancer: 1- and 2-year follow-up of a prospective controlled study. J Clin Oncol 2005;23(31):8025-32.

59. Jenkins V, Shilling V, Deutsch G*, et al.* A 3-year prospective study of the effects of adjuvant treatments on cognition in women with early stage breast cancer. Br J Cancer 2006;94(6):828-34.

60. Phillips KM, Jim HS, Small BJ*, et al.* Cognitive functioning after cancer treatment: a 3-year longitudinal comparison of breast cancer survivors treated with chemotherapy or radiation and noncancer controls. Cancer 2012;118(7):1925-32.

61. Schagen SB, Muller MJ, Boogerd W*, et al.* Change in cognitive function after chemotherapy: a prospective longitudinal study in breast cancer patients. J Natl Cancer Inst 2006;98(23):1742-5.

62. Brezden CB, Phillips KA, Abdolell M*, et al.* Cognitive function in breast cancer patients receiving adjuvant chemotherapy. J Clin Oncol 2000;18(14):2695-701.

63. Ernst T, Chang L, Cooray D*, et al.* The effects of tamoxifen and estrogen on brain metabolism in elderly women. J Natl Cancer Inst 2002;94(8):592-7.

64. Inagaki M, Yoshikawa E, Matsuoka Y*, et al.* Smaller regional volumes of brain gray and white matter demonstrated in breast cancer survivors exposed to adjuvant chemotherapy. Cancer 2007;109(1):146-56.

65. Lejbak L, Vrbancic M, Crossley M. Endocrine therapy is associated with low performance on some estrogen-sensitive cognitive tasks in postmenopausal women with breast cancer. J Clin Exp Neuropsychol 2010;32(8):836-46.

66. Myers JS, Wick JA, Klemp J. Potential factors associated with perceived cognitive impairment in breast cancer survivors. Support Care Cancer 2015;23(11):3219-28.

67. Silverman DH, Dy CJ, Castellon SA*, et al.* Altered frontocortical, cerebellar, and basal ganglia activity in adjuvant-treated breast cancer survivors 5-10 years after chemotherapy. Breast Cancer Res Treat 2007;103(3):303-11.

68. Brown J. Some tests of the decay theory of immediate memory. Quarterly Journal of Experimental Psychology 1958;10:12-21.

69. Sanders AF. Towards a model of stress and human performance. Acta Psychol (Amst) 1983;53(1):61-97.

70. Rey A. L'Examen clinique en psychologie. In. Paris: Presses Universitaires de France; 1964.

71. Brown FC, Roth RM, Saykin AJ*, et al.* A new measure of visual location learning and memory: development and psychometric properties for the Brown Location Test (BLT). Clin Neuropsychol 2007;21(5):811-25.

72. Miller EN, Satz P, Visscher B. Computerized and conventional neuropsychological assessment of HIV-1-infected homosexual men. Neurology 1991;41(10):1608-16.

73. Gualtieri CT, Johnson LG. Reliability and validity of a computerized neurocognitive test battery, CNS Vital Signs. Arch Clin Neuropsychol 2006;21(7):623-43.

74. Gualtieri CT, Johnson LG. A computerized test battery sensitive to mild and severe brain injury. Medscape J Med 2008;10(4):90.

75. Lezak MD. *Neuropsychological assessment*. 3rd ed. New York: Oxford University Press; 1995.

76. Gordon M, McClure F, Aylward G. The Gordon Diagnostic System Instruction Manual and Interpretive Guide. In. Dewitt, NY: Gordon Systems; 1986.

77. Delis D, Kramer J, Kaplan E*, et al.* California Verbal Learning Test: Adult Version. In. San Antonio, TX; 1987.

78. Delis D, Kaplan E, Kramer J. Delis-Kaplan Executive function System. In. San Antonio, TX: The psychological corporation; 1999.

79. Killgore WD, Glahn DC, Casasanto DJ. Development and Validation of the Design Organization Test (DOT): a rapid screening instrument for assessing visuospatial ability. J Clin Exp Neuropsychol 2005;27(4):449-59.

80. Aaronson NK, Ahmedzai S, Bergman B*, et al.* The European Organization for Research and Treatment of Cancer QLQ-C30: a quality-of-life instrument for use in international clinical trials in oncology. J Natl Cancer Inst 1993;85(5):365-76.

81. Wagner LI, Sweet JJ, Butt Z*, et al.* Measuring patient self-reported cognitive function: Development of the Functional Assessment of Cancer Therapy–Cognitive Function Instrument. The Journal of Supportive Oncology 2009;7(6):W32-W39.

82. Faust D, Fogel BS. The development and initial validation of a sensitive bedside cognitive screening test. J Nerv Ment Dis 1989;177(1):25-31.

83. Brandt J, Benedict R. Hopkins Verbal Learning Test-revised professional manual. In: Lutz, (ed). FL: Psychological assessment resources; 2001.

84. Van der Elst W, Dekker S, Hurks P, *et al.* The letter digit substitution test: demographic influences and regression-based normative data for school-aged children. Arch Clin Neuropsychol 2012;27(4):433-9.

85. Nasreddine ZS, Phillips NA, Bedirian V, *et al.* The Montreal Cognitive Assessment, MoCA: a brief screening tool for mild cognitive impairment. J Am Geriatr Soc 2005;53(4):695-9.

86. Troyer AK, Rich JB. Psychometric properties of a new metamemory questionnaire for older adults. J Gerontol B Psychol Sci Soc Sci 2002;57(1):P19-27.

87. Fischer J, Jak A, Kniker J. Multiple Sclerosis Functional Composite Administration and Scoring Manual. In. New York: National Multiple Sclerosis Society; 2001.

88. Tiffin J, Asher EJ. The Purdue pegboard; norms and studies of reliability and validity. J Appl Psychol 1948;32(3):234-47.

89. Meyers J, Meyers K. Rey-Complex Figure Test under four different administration procedures. The Clinical Neuropsychologist 1995;9:63-67.

90. Osterrieth PA. Le Test de Copie d'une figure complex: contribution a l'etude de la perception et de la memoire (The Complex Figure Copy Test). Archives de Psychologie 1944;30:286-356.

91. Rey A. L'examen psychologique dans les cas d'encephalopathie traumatique (The psychological examination in cases of traumatic brain injury). Archives de Psychologie 1942;28:286-340.

92. Aschenbrenner S, Tucha O, Lange K. RWT. Regensburger Wortflussigkeits-Test. In. Gottingen, Germany; 2000.

93. Squire LR, Zouzounis JA. Self-ratings of memory dysfunction: different findings in depression and amnesia. J Clin Exp Neuropsychol 1988;10(6):727-38.

94. Zimmermann P, Fimm B. Test of Attentional Performance, version 2.2. In. Herzogenrath, Germany; 2009.

95. Army Individual Test Battery: Manual of directions and scoring. In: War Department AGsO, (ed). Washington, DC; 1944.

96. Wechsler D. Wechsler Adult Intelligence Scale-3rd edition (WAIS-III). In. San Antonio, TX: Psychological Corportation; 1997.

97. The Psychological Corporation: Weschler Abbreviated Scale of Intelligence Scale. In. San Antonio, TX; 1999.

98. Kalverboer A, Deelman B. 1986. In; De 15-woordentest A en B. Groningen, the Netherlands, Academisch Ziekenhuis.

99. Wechsler D. Wechsler Memory Scale-Revised (WMS-R). In. San Antonio, TX: Psychological Corportation; 1987.

100. Wilkinson G, Robertson G. WRAT4 Wide Range Achievement Test Professional Manual. In. Psychological Assessment Resources, Inc; Futz, FL; 2006.

101. Van der Elst W, Van Boxtel MP, Van Breukelen GJ, *et al.* Normative data for the Animal, Profession and Letter M Naming verbal fluency tests for Dutch speaking participants and the effects of age, education, and sex. J Int Neuropsychol Soc 2006;12(1):80-9.

102. Boehmer U, Ozonoff A, Timm A*, et al.* After breast cancer: sexual functioning of sexual minority survivors. J Sex Res 2014;51(6):681-9.

103. Safarinejad MR, Shafiei N, Safarinejad S. Quality of life and sexual functioning in young women with early-stage breast cancer 1 year after lumpectomy. Psychooncology 2013;22(6):1242-8.

104. Vazquez-Ortiz J, Antequera R, Picabia AB. Ajuste Sexual e imagen corporal en mujeres mastectomizadas por cancer de mama. Psicooncologia 2010;7(2-3):433-451.

105. Rosen R, Brown C, Heiman J*, et al.* The Female Sexual Function Index (FSFI): a multidimensional self-report instrument for the assessment of female sexual function. J Sex Marital Ther 2000;26(2):191-208.

106. Sherbourne CD. Social functioning: sexual problems measures. In: Stewart AL, Ware JE, (eds). *Measuring Functioning and Well-Being*. Durham, NC: Duke University Press; 1992, 194-204.

107. Aluja A, Torrobuia R, Gallart D. Validacion espanola del autoinforme de ansiedad y excitacion sexual ampliado (SAI-E). Rev Psiquiatr Fac Med Barc 1990;27(6):252-68.

108. Chambless DL, DeMarco D. Women's Sexuality Questionnaire. In: 3rd, (ed). *Handbook of Sexuality-Related Measures*. New York, US: Routledge; 2011, 263-267.

109. Gurevich M, Devins GM, Wilson C*, et al.* Stress response syndromes in women undergoing mammography: a comparison of women with and without a history of breast cancer. Psychosom Med 2004;66(1):104-12.

110. Voigt V, Neufeld F, Kaste J*, et al.* Clinically assessed posttraumatic stress in patients with breast cancer during the first year after diagnosis in the prospective, longitudinal, controlled COGNICARES study. Psychooncology 2017;26(1):74-80.

111. El Rafihi-Ferreira R, Nogueira Pires ML, Zoega Soares MR. Sleep, quality of life and depression in women in breast cancer post-treatment. Psicologia: Reflexao e Critica 2011;25(3):506-513.

112. Schairer C, Brown LM, Chen BE*, et al.* Suicide after breast cancer: an international population-based study of 723,810 women. J Natl Cancer Inst 2006;98(19):1416-9.

113. Fang F, Fall K, Mittleman MA*, et al.* Suicide and cardiovascular death after a cancer diagnosis. N Engl J Med 2012;366(14):1310-8.

114. Buysse DJ, Reynolds CF, 3rd, Monk TH*, et al.* The Pittsburgh Sleep Quality Index: a new instrument for psychiatric practice and research. Psychiatry Res 1989;28(2):193-213.

115. Cardena E, Koopman C, Classen C*, et al.* Review of the Stanford Acute Stress Reaction Questionnaire. In: Stamm B, (ed). *Measurement of stress, trauma and adaptation*. Lutherville, MD: Sidran Press; 1996.
